# Patient-Derived Tumor Organoids for Guidance of Personalized Drug Therapies in Recurrent Glioblastoma

**DOI:** 10.3390/ijms23126572

**Published:** 2022-06-12

**Authors:** Miriam Ratliff, Hichul Kim, Hao Qi, Minsung Kim, Bosung Ku, Daniel Dominguez Azorin, David Hausmann, Rajiv K. Khajuria, Areeba Patel, Elena Maier, Loic Cousin, Arnaud Ogier, Felix Sahm, Nima Etminan, Lukas Bunse, Frank Winkler, Victoria El-Khoury, Michael Platten, Yong-Jun Kwon

**Affiliations:** 1 Department of Neurosurgery, Mannheim Center for Translational Neurosciences (MCTN), Medical Faculty Mannheim, University of Heidelberg, 68167 Mannheim, Germany; rajiv.khajuria@dkfz-heidelberg.de (R.K.K.); elena.maier@umm.de (E.M.); nima.etminan@umm.de (N.E.); 2 Clinical Cooperation Unit Neurooncology, German Cancer Consortium (DKTK), German Cancer Research Center (DKFZ), 69120 Heidelberg, Germany; d.dominguezazorin@dkfz-heidelberg.de (D.D.A.); d.hausmann@dkfz-heidelberg.de (D.H.); frank.winkler@med.uni-heidelberg.de (F.W.); 3 Personalized Therapy Discovery, Department of Cancer Research, Luxembourg Institute of Health, 3555 Dudelange, Luxembourg; hichul.kim@1stbio.com (H.K.); victoria.elkhoury@lih.lu (V.E.-K.); 4 Early Discovery and Technology Development, Ksilink, 67000 Strasbourg, France; loic.cousin@ksilink.com (L.C.); arnaud.ogier@ksilink.com (A.O.); 5 Clinical Cooperation Unit Neuroimmunology and Brain Tumor Immunology, German Cancer Consortium (DKTK), German Cancer Research Center (DKFZ), 69120 Heidelberg, Germany; qihao19930915@gmail.com (H.Q.); lukas.bunse@umm.de (L.B.); michael.platten@umm.de (M.P.); 6 Department of Biomedical Science, Seoul National University College of Medicine, Seoul 110799, Korea; minsung960523@snu.ac.kr; 7 Central R&D Center, Medical & Bio Decision (MBD), Suwon 16229, Korea; goos4684@gmail.com; 8 Neurology Clinic and National Center for Tumor Diseases, University Hospital Heidelberg, 69120 Heidelberg, Germany; 9 Department of Neuropathology, University Hospital Heidelberg and CCU Neuropathology, German Consortium for Translational Cancer Research (DKTK), German Cancer Research Center (DKFZ), 69120 Heidelberg, Germany; a.patel@dkfz-heidelberg.de (A.P.); felix.sahm@med.uni-heidelberg.de (F.S.); 10 Mannheim Center for Translational Neurosciences (MCTN), Department of Neurology, Medical Faculty Mannheim, University of Heidelberg, 68167 Mannheim, Germany; 11 Luxembourg Center of Neuropathology (LCNP), Department of Cancer Research, Luxembourg Institute of Health, 3555 Dudelange, Luxembourg; 12 DKFZ Hector Cancer Institute, University Medical Center Mannheim, 68167 Mannheim, Germany

**Keywords:** glioblastoma, patient-derived organoids, personalized oncology, drug profiling, precision medicine, tumor microtube (TM), tumor cell network, intercellular calcium waves (ICW)

## Abstract

An obstacle to effective uniform treatment of glioblastoma, especially at recurrence, is genetic and cellular intertumoral heterogeneity. Hence, personalized strategies are necessary, as are means to stratify potential targeted therapies in a clinically relevant timeframe. Functional profiling of drug candidates against patient-derived glioblastoma organoids (PD-GBO) holds promise as an empirical method to preclinically discover potentially effective treatments of individual tumors. Here, we describe our establishment of a PD-GBO-based functional profiling platform and the results of its application to four patient tumors. We show that our PD-GBO model system preserves key features of individual patient glioblastomas in vivo. As proof of concept, we tested a panel of 41 FDA-approved drugs and were able to identify potential treatment options for three out of four patients; the turnaround from tumor resection to discovery of treatment option was 13, 14, and 15 days, respectively. These results demonstrate that this approach is a complement and, potentially, an alternative to current molecular profiling efforts in the pursuit of effective personalized treatment discovery in a clinically relevant time period. Furthermore, these results warrant the use of PD-GBO platforms for preclinical identification of new drugs against defined morphological glioblastoma features.

## 1. Introduction

Glioblastomas are the most common primary malignant brain tumors in adults [1]. They are characterized by their aggressive and invasive nature and exhibit high inter- and intratumoral heterogeneity. For newly diagnosed glioblastoma patients, surgery followed by radiotherapy and temozolomide chemotherapy represents the current standard of care. However, for patients with a tumor recurrence, there is a lack of therapeutic options that satisfactorily improve patient outcome [2]. The median overall survival after disease progression remains poor (~8 months) [3]. This poor prognosis after regrowth is in part explained by the fact that current treatment regimens do not consider the molecular and cellular heterogeneity between tumors of different patients. This heterogeneity is likely even more complex in recurrent glioma. Thus, novel approaches are urgently needed for personalized treatment. Modern approaches largely rely solely on molecular profiling of tumors, which is often insufficient to accurately predict therapy response. Therefore, complementing this approach with phenotypical functional profiling to assess treatment responses should improve guidance for personalized drug therapies.

To enable large-scale screens and the timely identification of an evidence-based, personalized drug treatment, suitable preclinical glioblastoma models are needed. A good model should reliably recapitulate the actual patient tumor with regard to heterogeneity, cell–cell communication, and interaction of glioma cells with their tumor microenvironment (TME) [4,5,6,7,8,9]. Patient-derived glioblastoma organoids (PD-GBOs) have emerged as more accurate and potentially feasible preclinical tumor models. PD-GBOs are small, viable spheroidal structures that are established from resected tumor tissue. They retain many molecular, structural, and functional aspects of the patient tumors from which they are derived, including genomic architecture, gene expression profiles, and cellular diversity [5,10,11].

From both a clinical and a preclinical point of view, minimization of the time that PD-GBOs are cultured is desirable. First, there is a risk of clonal drifting over prolonged culture times that could result in a loss of molecular and cellular diversity present in the patient’s tumor in vivo [12]. Second, the cellular components of the TME are progressively lost over time in culture. The TME is critical for preserving the recapitulation of tumor progression and accurate projection of responses to diverse therapies. Third, a PD-GBO-based drug screening platform should reveal a potential personalized therapy approach within 4 weeks of initial culture to be practically relevant in a clinical setting.

Guided by these considerations, we established a PD-GBO-based functional profiling platform and, as proof of concept, applied it to four patient tumors. Here, we report our evaluation of this platform and its use to screen a panel of FDA-approved drug candidates (see graphical abstract for an overview of this process). Three of the four screens produced actionable results within a period of about two full weeks.

## 2. Results

### 2.1. PD-GBOs Can Be Generated from Resected Tumor Tissue

**Figure 1:**
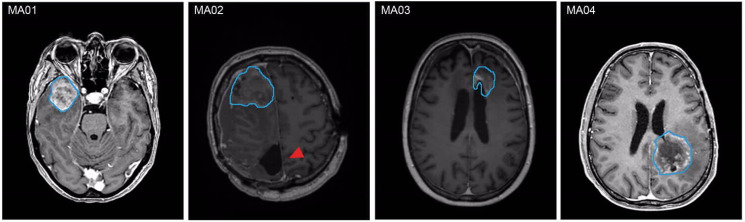
MRI characteristics of the patients included in this study. MRIs show contrast-enhanced, suspected recurrent high-grade glioma prior to re-resection. Outlined in blue is the resection cavity after the second surgical treatment with partial or complete resection of the contrast-enhanced tumor. In the MRI of patient MA02, the resection cavity from initial surgery is marked with a red arrowhead; in this case, the plane of the resection cavity is within the same axial plane as the recurrent contrast enhancement.

Resected tumor tissue from four high-grade glioma patients harboring tumors in different anatomical regions was used for generation of tumor organoids (Figure 1; Table 1). MA01–MA03 were previously diagnosed as glioblastoma WHO grade 4, *IDH* wildtype; MA04 was diagnosed as astrocytoma WHO grade 4, *IDH* mutant (Table 1). All patients received adjuvant chemotherapy and radiation following initial resection. We performed mutation analysis using next-generation sequencing of 130 genes known to be relevant to glioblastoma (panel version NPHD2015A). The molecular tissue analysis did not reveal a specific target for any of the patients (data not shown).

### 2.2. PD-GBOs Recapitulate Actual Glioblastoma Properties

**Figure 2:**
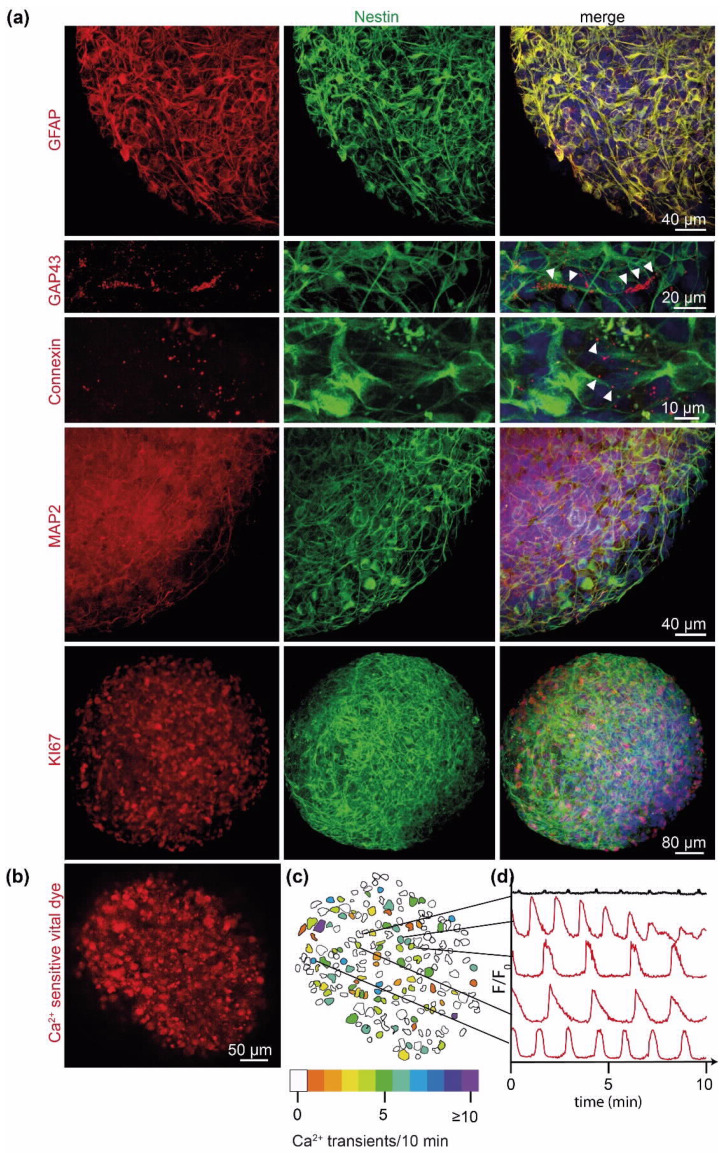
Patient-derived glioblastoma organoids (PD-GBOs) preserve features of glioblastoma tumor tissue. (**a**) Exemplary immunofluorescence staining proteins common to glioblastoma (see text for references): GFAP, GAP43, Connexin 43, MAP2, and Ki67 (all shown in red), nestin (green), and DNA stained with Hoechst3342 (blue). All images were acquired by confocal microscopy of PD-GBOs from patient MA03. Arrowheads mark GAP43 and Connexin 43 found alongside TMs, as observed in human glioblastoma tumor tissue [14]. TM connections facilitate communication in multicellular networks [13,14,15,16,17,18]. (**b**–**d**) PD-GBO cells transmit calcium transients (image and data from Supplemental Movie S1). (**b**) Optical section from a representative 14 day old PD-GBO after incubation with fluorescent mitochondrial dye (Rhod-2 AM) to visualize calcium transients. (**c**) Tracing of cell bodies from (**b**) and color-coding to identify and discriminate participating cells on the basis of frequency of calcium transients within a representative 10 min time period. (**d**) Chart of calcium transients (ΔF/F0) of individual, but TM-connected tumor cells identified in (**c**). Note the absence of calcium transients in a nonparticipating cell (top black line). Calcium transients travel along TMs of glioblastoma tumor cells, leading to synchronous calcium peaks in TM-connected cells [14,15,18,19].

We next evaluated whether our generated PD-GBOs actually resemble glioblastomas in vivo. We observed that the tumor cells of PD-GBOs maintained their proliferative capacity in culture and found that expression of specific markers known to be expressed in glioblastoma tumors in vivo was retained (Figure 2a). The established PD-GBOs shared known morphological features of glioblastoma including the presence of GFAP and nestin-labeled tumor microtubes (TMs), extensions that connect individual glioblastoma tumor cells to each other. TMs facilitate multicellular communication and also promote primary and acquired resistance to diverse therapies [13,14,15,16,17]. Thus, their presence within a model to assess drug responses is of particular importance. TMs within PD-GBOs expressed Gap43, with immunoreactivity detected alongside their membranes. Connexin 43 was expressed at the contact sites of TMs. The presence and localization of these TM markers within these organoids perfectly overlapped with their described expression in vivo [13,14,15,16,17] (Figure 2a). We also performed timelapse recordings of calcium transients in PD-GBOs and observed dynamic global calcium signaling that was partially synchronized among neighboring cell clusters (Figure 2b–d, Supplemental Movie S1). This signaling activity validated the functional relevance of the organoid tumor microtubes. Overall, these results suggest that these PD-GBOs strongly resemble their glioblastoma in vivo counterpart. 

### 2.3. Personalized 3D Drug Screens Using PD-GBOs

**Figure 3:**
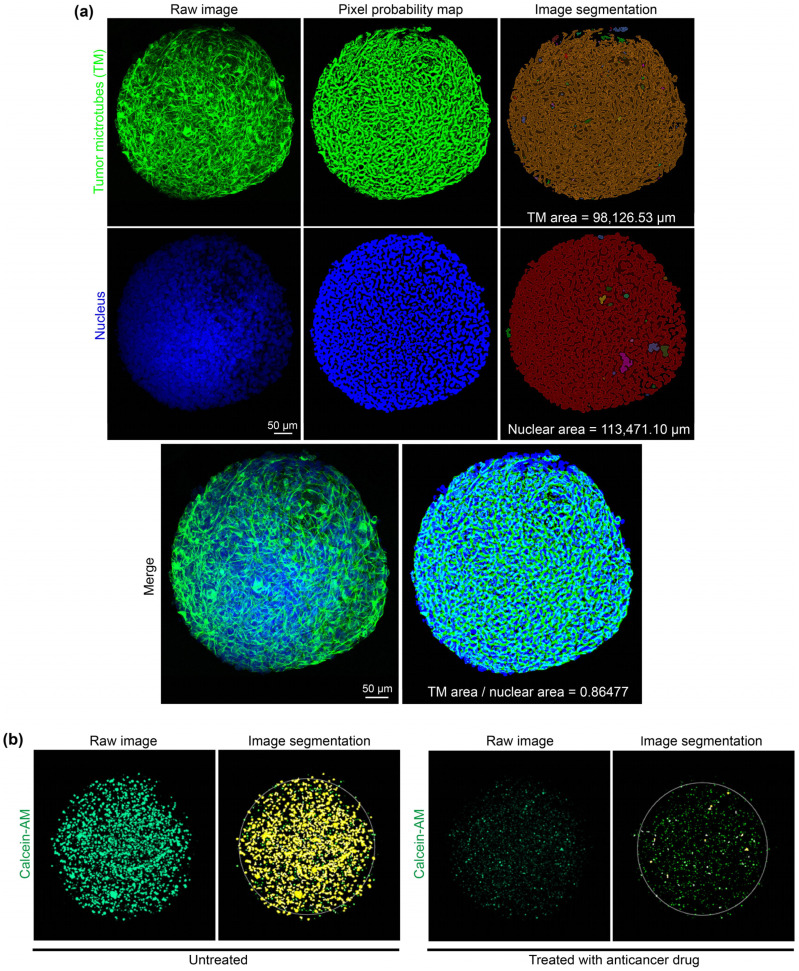
Automated computational analysis of TM network density and cell viability in PD-GBOs. (**a**) Quantification of the TM-connected tumor network used nestin (top left image, green), which is highly expressed in network-integrated glioma cells. The areas of the nuclei (blue Hoechst stain, middle-left images) and tumor network (nestin) were converted to pixels (top- and middle-center image) and then segmented (top- and middle-right images). The quotient of nestin area by nuclear area provides an index for tumor network density. (**b**) Quantification of cell viability used the cell viability dye, calcein-AM (left image). Shown are representative images of PD-GBOs from patient MA01. The untreated control PD-GBO (left images) has significantly more viable cells compared to the PD-GBO after afatinib treatment (30 μM; right images).

Next, we used organoids derived from four recurrent high-grade glioma patient tumors in a phenotypic screen of a panel of 41 FDA-approved drugs. We implemented deep-learning segmentation using AIVIA Software 9.8.1 (DRVISION Technologies) to quantify morphological characteristics of interest for phenotypic drug screening in PD-GBOs. Segmentation harnessed automated the quantification of tumor network complexity and cell viability (Figure 3). Further details on the analysis of functional drug testing are provided in the Supplementary Methods.

**Figure 4:**
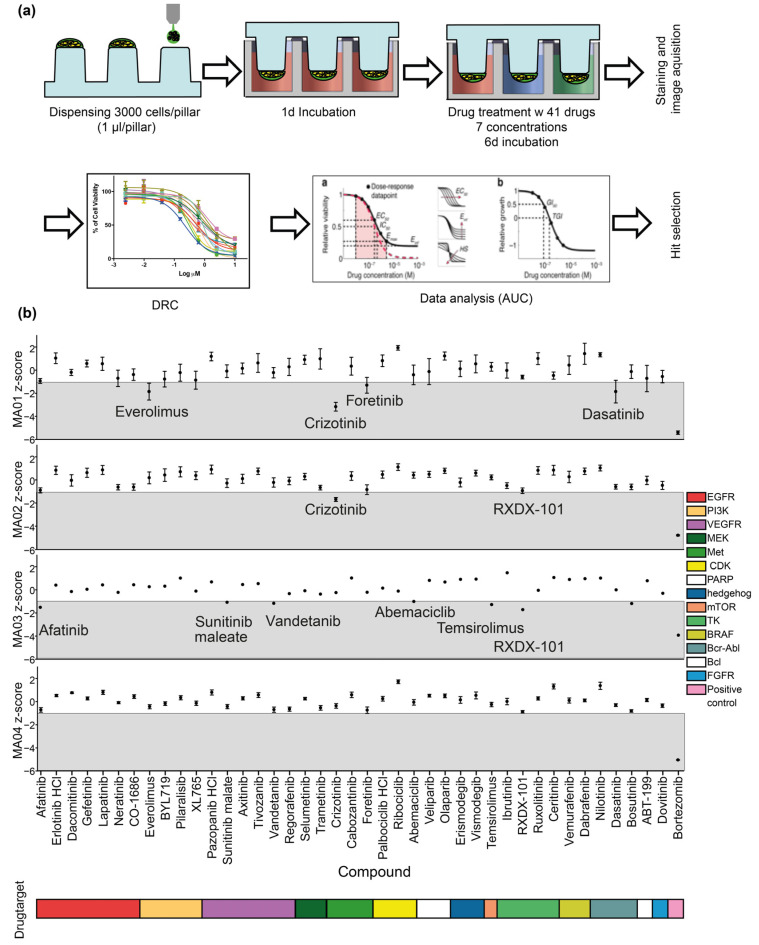
Personalized drug screening. (**a**) Schematic workflow of PD-GBO culture and personalized drug screening. Following a few days of acute spheroid culture, patient-derived tumor cells were printed to form PD-GBOs with alginate as an adhesion matrix on a 384-pillar array using an ASFA spotter. Forty-one FDA-approved drugs were tested in fourfold and seven-point serial dilutions for 6 days. The imaging of calcein-AM-labeled PD-GBOs was semiautomated. We quantified the viable cell population within the PD-GBOs during image analysis, and then used our proprietary software that implements the DRC tool to select candidate drugs (see Supplementary Methods). Thus, about 2 weeks after tumor resection, actionable information was produced for potential clinical application. (**b**) Screening results of PD-GBOs with 41 FDA-approved drugs. Scatter plot of area-under-the-curve ratios by serial diluted (fourfold, seven-point) compound treatments and identification of potential drug candidates in three out of four patients (MA01–MA03) with recurrent glioblastoma.

The resected human glioblastoma samples were dissociated on the same day and cultured for 1–2 weeks until acute tumor spheroids formed. We used a single cell suspension of those acute tumor spheroids to print PD-GBOs on the cell pillar plates. Twenty-four hours after seeding of the PD-GBOs, we initiated drug testing. PD-GBOs were exposed to the drugs for 6 days. Results from our functional screening were reported 14–19 days after resection (Table 1). On our platform, all PD-GBO samples were responsive to high concentrations of bortezomib based on cell viability, which was used as a positive control and served as a verification of the experimental setup (Figure 4a). The area under the curve (AUC) was calculated for each drug–response curve (DRC) (see Supplementary Methods, Supplementary Figure S1, and Supplementary Table S1). The z-score indicates how much the experimental AUC differs statistically from each drug response. In our experience a z-score <−0.5 predicts a partial or complete response in cases where the identified drug was applied clinically [20]. In MA01 PD-GBOs, four drugs (everolimus, crizotinib, foretinib, and dasatinib), which target different molecules, produced a z-score <−1, suggesting that these were promising candidates for a personalized therapy regimen. In MA02 PD-GBOs, only crizotinib produced a reasonable therapeutic efficacy (z-score <−1). Afatinib and RXDX-101 were identified as potent drugs in MA03 PD-GBOs. In MA04 PD-GBOs, with the exception of the positive control bortezomib, none of the 41 drugs tested generated a z-score <−1 (Figure 4b). In sum, we used our platform to identify potent drug candidates for three out of four glioblastoma patients within 15 days of tumor resection, a time period that is clinically relevant for personalized therapy approaches.

### 2.4. Transcriptome Analysis of Parental Tumor Tissue

**Figure 5:**
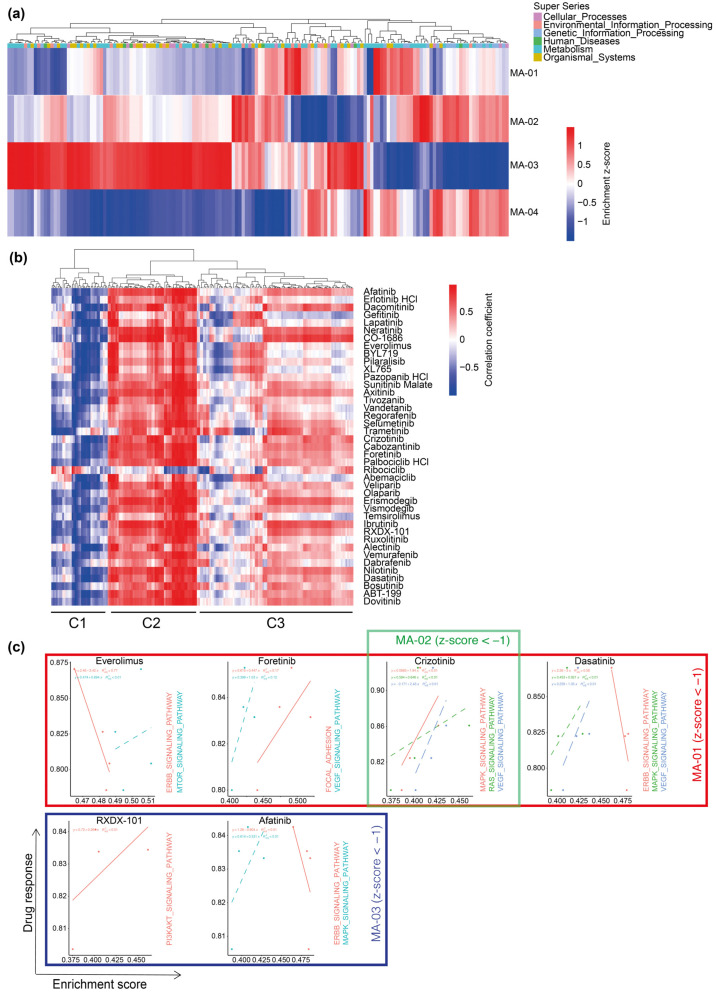
Correlation of pathway enrichment in the parental tumor and drug response of corresponding PD-GBOs. (**a**) Heatmap illustrating inter-sample z-score of 193 KEGG pathways enrichment levels from four high-grade glioma tissues. KEGG pathway super series groups are color-coded. Raw values are provided in Supplementary Table S2. (**b**) Pearson correlation coefficient of drug response score and specific interindividual KEGG pathway enrichment levels visualized as a heatmap. On the basis of the correlation coefficient with drug response, pathways are divided into three clusters (C1, C2, and C3). For individual pathways within clusters, see Supplementary Table S3. (**c**) Linear regression of PD-GBO drug response score and enrichment of annotated biologically related pathways (KEGG drug database).

We then assessed how our phenotypic response screen compared to molecular profiling in the identification of potential drugs for personalized therapies. We carried out RNA-sequencing of the resected tumor tissue (Supplementary Figure S2a) and investigated whether an enrichment of distinct Kyoto Encyclopedia of Genes and Genomes (KEGG) pathways was predictive for the response of PD-GBOs to our panel of 41 drugs (Supplementary Figure S2b). Interindividual comparisons revealed differential enrichment of pathways (Figure 5a, Supplementary Table S2), a result that reflects interindividual tumor heterogeneity and highlights the need for new personalized therapy approaches. We then sought, in an unbiased analysis, the correlation of drug response and specific interindividual KEGG pathway enrichments (Supplementary Table S3). In cluster C1, response to all FDA-approved drugs except for ribociclib was negatively correlated with KEGG pathway enrichment scores, whereas, in C2, they were positively correlated with KEGG pathway enrichment scores (Figure 5b). Interestingly, C3 defines a heterogeneous cluster with divergent correlations of pathway enrichment and drug response. In our PD-GBO cohort, a differential response was observed for everolimus, foretinib, crizotinib, dasatinib, RXDX-101, and afatinib. In a subsequent targeted analysis, we retrieved from the manually curated KEGG drug database specific drug-target pathways for the seven abovementioned drugs. Interestingly, ERBB pathway signaling was negatively correlated with response to treatment with the respective drugs everolimus, dasatinib, and afatinib (Figure 5c). However, MAPK pathway signaling was found to be positively correlated with response to treatment with the respective drugs crizotinib, dasatinib, and afatinib.

## 3. Discussion

In the present study, we generated patient-derived glioblastoma organoids (PD-GBOs) and subjected them to drug screening. We used an innovative, personalized functional drug-profiling technology that enables high-throughput automated drug screening of PD-GBOs. The methodology is partially automated, easy to use, and amenable to quantitative analysis [21,22]. The 3D cell structures are formed and cultured within their extracellular matrix in hanging droplets and fixed on pillars. There is no manual handling of the cell matrix spots once they are printed, thus avoiding the loss of cells or the damage of the PD-GBOs during the washing or medium replacement steps. The fully automated cell-printing process takes only 1.5 min for a 384-pillar plate. Changing medium simply requires moving the lid of the 384-pillar plate containing the cell matrix hanging structures to another 384-well plate containing the desired medium (Figure 4a).

PD-GBOs preserve structural and physiological characteristics of the parental tumor. Tumor cells within the PD-GBO form a morphological interconnecting tumor network proficient for nestin, Gap43, Connexin 43, and the astrocytic marker GFAP (Figure 4b) [13,14,16,17]. We detected spontaneous calcium transients in PD-GBOs as previously observed in vivo [14]. The intercellular calcium waves can be quantified and used as an additional sophisticated readout for future drug screens (Figure 2b). Overall, we conclude that PD-GBOs are phenotypically stable, miniature replicates of the parental resected tumor tissue. The tumor cell network renders its members resistant to cytotoxic and radiation therapies, likely due to cellular homeostasis within the connected cells [14,16]. We, therefore, emphasize the importance of a model system having a morphological and functional tumor network and hypothesize that phenotypic and functional drug profiling is superior to the more common biochemical drug response analysis.

The power of personalized functional drug profiling is that is can quickly pinpoint tumor responses for individual patients. These hits would otherwise be almost impossible to identify within a clinically relevant timeframe. In our hands, we were able to deliver promising drug treatment recommendations for three of four cases within 15 days of tumor tissue resection. Conversely, for one case, 19 days were needed to confirm no tumor response for any drug in our panel.

Among the four tumors tested in the drug screen, MA01 and MA02 had similar characteristics. Both tumors were *IDH* wildtype and showed distant tumor progression relative to the original resection cavity. Interestingly, only MA01 and MA02 shared a common hit drug, crizotinib. This raises the question of whether the shared genotype or phenotype of these tumors is specifically targeted by crizotinib. Of course, more tumor samples need to be analyzed to address this hypothesis.

The phosphatidylinositol 3-kinase (PI3K) signaling pathway is commonly dysregulated in cancer, including glioblastoma [23]. Its activation enhances tumor cell proliferation and migration, as well as promotes angiogenesis and cell survival. The mammalian target of rapamycin (mTOR) is a downstream component of the PI3K pathway and a potential therapeutic target. Some examples of successful treatments of glioblastoma patients with mTOR inhibitors have been reported, such as in the case of a child having a TSC2-mutant glioblastoma who achieved complete remission under everolimus therapy [24]. Nevertheless, despite the rationale behind targeting the PI3K/mTOR pathway, the general clinical results of mTOR inhibitors in the treatment of glioblastoma patients have been rather disappointing [25]. It is believed that inefficacy of mTOR inhibitors in clinic may stem from compensatory mechanisms activated upon mTOR inhibition [26]. If true, this would support rationally selected combinatorial strategies when using these drugs. This is, for example, what could be envisaged for patient MA01, whose tumor organoid was particularly sensitive to the mTOR inhibitor, everolimus, as well as to crizotinib, foretinib, and dasatinib, drugs that do not target mTOR.

The activation of the hepatocyte growth factor/mesenchymal–epithelial transition (MET) pathway is implicated in the progression of glioblastoma, as it activates downstream signaling events including Ras/Raf/MAPK, STAT3, and PI3K/AKT/mTOR. As a consequence, MET inhibitors demonstrate clinical efficacy when initially administered to glioblastoma patients; however, unfortunately, the acquired resistance to these agents frequently ensues and hampers their prolonged use [27]. Crizotinib is a multitarget tyrosine kinase inhibitor, acting as an MET inhibitor, anaplastic lymphoma kinase (ALK) inhibitor, and ROS1 inhibitor. It is an FDA-approved drug for the treatment of patients with metastatic non-small-cell lung cancer (NSCLC) whose tumors are ALK-positive or ROS1-positive. Bypass mechanisms to crizotinib and another MET inhibitor, onartuzumab, were recently discovered in glioblastoma. Cruickshanks et al. found that targeting mTOR, FGFR1, EGFR, STAT3, and COX-2 in combination with MET inhibitors re-sensitizes tumor cells to inhibition of MET [28]. Therefore, because patient MA01 tumor organoids were responsive to both crizotinib and the mTOR inhibitor, everolimus, the treatment of the patient with a combination of these drugs might have been clinically effective. While a tumor organoid of patient MA02 was also responsive to crizotinib, we did not identify a drug targeting compensatory pathways, as crizotinib was the sole drug identified for MA02. Perhaps expansion of the drug panel would have identified additional candidates to serve this purpose.

Foretinib was identified in our screen using patient MA01 tumor organoids and is an MET/VEGFR inhibitor. It is an investigational drug currently tested in clinical trials for solid cancers. The ability of foretinib to inhibit the TAM family members of receptor tyrosine kinases (Tyro3, Axl, and MerTK) hindered the survival, proliferation, and migration of glioblastoma cell lines in vitro and reduced glioblastoma tumor growth in a subcutaneous mouse model [29]. These findings support its translational therapeutic potential in glioblastoma.

Dasatinib is a potent inhibitor of the members of the Src kinase family (SKF). Among other targets, it inhibits BCR-Abl (breakpoint cluster region–Abelson murine leukemia) and is, therefore, used in the treatment of chronic myelogenous leukemia (CML) that is associated with the Philadelphia chromosome [30]. Considering the evidence of an overactivation of Src kinases in glioblastoma, there is a rationale for targeting Src in this malignancy [31]. Unfortunately, dasatinib showed a lack of efficacy against target-selected patients with recurrent glioblastoma in a phase II trial [32]. In line with this trial, its combination with standard radiotherapy and temozolomide in newly diagnosed glioblastoma patients (phase I/II trial N0877) or with bevacizumab in patients with recurrent glioblastoma (phase II trial N0872) did not improve clinical outcome [33,34]. One potential factor for the failure of dasatinib in its clinic application is that it is a substrate of ATP-binding cassette transporters P-glycoprotein (ABC) and breast cancer resistance protein (BCRP/ABCG2), gatekeepers of the blood–brain barrier [35,36,37]. Accordingly, it is not a valid option in a clinical setting for patient MA01, despite its identification in our screen.

EGFR is overexpressed in nearly 60% of glioblastoma tumors, often the consequence of gene amplification (in 40% of these tumors) or the common EGFRvIII deletion (in 25% of these tumors), and it is considered a poor prognostic factor. The reversible EGFR tyrosine kinase inhibitors gefitinib and erlotinib are commonly used in lung cancer treatment, but they failed to demonstrate an overall clinical benefit in recurrent glioblastoma [38,39]. Nevertheless, a subset of patients with a complex EGFR genotype responded remarkably to combined treatment with the irreversible EGFR inhibitor afatinib and temozolomide [3,40]. Interestingly, afatinib was identified as a promising drug in this study for patient MA03 tumor organoids, but not gefitinib and erlotinib. Patient MA03 is, thus, reminiscent of the aforementioned subset of complex EGFR genotype cases and would warrant a combined treatment of afatinib and temozolomide.

Unfortunately, the drug screen did not identify a promising drug option for patient MA04. However, at the same time, it gave an indication of the drugs to be avoided because their administration may only expose the patient to unnecessary side-effects and mental hardships.

The results of our study of personalized functional profiling in recurrent glioblastoma reported here have caveats. The 3D tumor organoid model we used does not account for the blood–brain barrier, one of the major obstacles in brain cancer chemotherapy and a frequent reason for treatment failure. Moreover, despite being generated from a patient tumor, each PD-GBO does not fully model all aspects of the glioblastoma microenvironment in vivo, which includes blood vessels and infiltrating immune cells. While this 3D glioblastoma model is more complex compared to previous 2D models, it may have to be further refined in order to yield results that are more accurate and, thus, improve patient outcome.

We proved the concept of screening a drug panel with PD-GBOs from a limited cohort of patients with recurrent glioblastoma in a clinically relevant timeframe correlating PD-GBOs specific drug responses with the patients’ mutational profile. There is evidence that mutational analysis alone, without functional testing, is insufficient to predict treatment responses [11]. It is yet unclear if personalized functional drug testing is a complement or even an alternative to gene expression analysis in the guidance of targeted treatment decisions.

This pilot study is a promising step further toward an early go/no go decision-making model system for patients diagnosed with recurrent glioblastomas. We plan to further evaluate our PD-GBO model to determine whether PD-GBOs are accurate mimics of the clinical parental glioblastoma and if treatment options based upon them optimize individual patient outcomes.

## 4. Materials and Methods

### 4.1. Drug Library Preparation

The US Food and Drug Administration (FDA)-approved investigational drug library (41 compounds, 10 mM in DMSO; bortezomib as a positive control) and the drug library used for comparative response analysis (Supplemental Figure S3 and Supplemental Table S4) were purchased from Selleckchem Inc. (Houston, TX, USA), except for LNT1, which was purchased from Tocris. The compounds used in the screen on PD-GBOs are known anticancer molecular agents including inhibitors of epidermal growth factor (EGFR), fibroblast growth factor (FGFR), vascular endothelial growth factor (VEGFR), mechanistic target of rapamycin (mTOR), and phosphoinositide 3-kinase (PI3K). The compounds of the library were transferred to a 384-well assay plate (Greiner Bio-One) that was designed as a fourfold and seven-point serial dilution series (maximum 30 µM in 40 µL of culture medium) using the Echo 550 liquid handler (Labcyte, San Jose, CA, USA). Prepared drug assay plates were sealed and stored at −80 °C until further use. We used 80 different drugs, with each concentration tested twice for the comparative response analysis on printed U87 spheroids. A total of 13 drugs were used for both comparative response analysis and PD-GBO screening.

### 4.2. Glioblastoma Resection and Tissue Processing

This study was conducted as approved by the institutional review board (Ethics committee at the Mannheim Medical Faculty of the University of Heidelberg; 2018-598N-MA), and written informed consent was obtained from all participants. Patients were previously diagnosed with high-grade glioma according to the current World Health Organization (WHO) criteria [41]. On the basis of follow-up MRIs, tumor recurrence was suspected in the patients included in this study (Figure 1).

Resections were performed in 2019 and 2020 according to the standards of our hospital. The surgically resected tumor specimens were partitioned into three samples used for (I) histological and molecular diagnostics (Table 1), (II) generation of patient-derived glioblastoma organoids (PD-GBOs) and subsequent drug testing, and (III) cancer panel sequencing and transcriptome analysis [42]. We documented the weight of the resected solid tumor tissue, location of the resected tumor tissue in relation to the previous resection cavity, and the highest proliferation index (Ki67) found within the tissue sent for histological analysis (Table 1).

### 4.3. Establishment of Acute Glioblastoma Spheroids

The tumor tissue was enzymatically dissociated into a single-cell suspension as described previously [20]. In brief, tumor tissue was washed with cold PBS to remove debris and blood, and then minced into small pieces (~5 mm^3^) using micro dissecting scissors. Minced tissues were incubated at 37 °C for 40 min in DMEM medium (Gibco, Waltham, MA, USA) supplemented with collagenase I (200 units/mL; Gibco), DNase (100 µg/mL; Roche Molecular Biochemical), and Dispase (500 µg/mL; Gibco). After digested tissue was washed several times to remove enzymes, dissociated glioblastoma cells were cultured in ultralow-attachment culture dishes (Corning) with neurobasal-A medium supplemented with N2 (10 µg/mL; Gibco), B27 (10 µg/mL; Gibco), L-glutamine (4 µg/mL; Gibco), human recombinant basic fibroblast growth factor (25 ng/mL bFGF; R&D Systems, Minneapolis, RN, USA), and epidermal growth factor (25 ng/mL EGF; R&D Systems). Cells were cultured for 1–2 weeks for acute spheroid formation. The medium was changed every 3 days.

### 4.4. Cell Printing and Drug Screening on the 384-Pillar Array

Acute spheroids were dissociated into single cells using StemPro Accutase (Gibco). The cell suspension was mixed with 1% alginate (ratio of 1:1) and printed on a 384-pillar plate (3000 cells per pillar, in a volume of 1 µL) using the ASFA Spotter ST (Medical & Bio Device, Suwon, Korea). The pillar plate was left undisturbed for 1 min 50 s to allow the alginate to set, combined with the 384-well plate containing culture medium, and incubated for 40 min at 37 °C and 5% CO_2_ in a humidified incubator. The cell pillar plate was transferred to a new 384-well plate containing fresh medium, and the cells were cultured for 1 day at 37 °C and 5% CO_2_. Then, the pillar plate was combined with the drug assay plate (in duplicate), and the cells were incubated with the compounds at different concentrations for 6 days (Figure 4a). PD-GBO samples treated with vehicle only (DMSO) served as the negative control. As the positive control for the induction of cell death, treatment with the protease inhibitor bortezomib was used at dosages (fourfold serial dilutions with a maximal dose of 30 µM) significantly above clinically relevant concentrations.

We used the U87 glioblastoma cell line for comparative response analysis utilizing the biochemical ATP-based assay (CellTiter-Glow, Promega, Madison, WI, USA) and the phenotypical calcein-AM assay. Spheroid printing and drug screening were performed as described above. The drugs used are listed in Supplementary Table S4.

### 4.5. Immunofluorescence, Histology, and Immunohistochemistry

To further analyze the cellular composition of the generated PD-GBOs, immunofluorescence staining was performed by washing PD-GBOs with phosphate-buffered saline (PBS) and fixing them with 4% (*w*/*v*) paraformaldehyde in PBS for 30 min at room temperature. The following primary antibodies were used: anti-nestin (1:400, ab6320, Abcam, Cambridge, UK, RRID:AB_308832), anti-Connexin 43 (1:2000, C6219, Sigma-Aldrich, St. Louis, MO, USA, RRID:AB_476857), anti-GAP43 (1:200, #8945, Cell Signaling Technology, Danvers, MA, USA, RRID:AB_10860076), anti-GFAP (1:500, Z0334, Agilent-Technologies, Santa Clara, CA, USA, RRID:AB_10013382), anti-Ki67 (1:500, ab15580, Abcam, RRID:AB_443209), and anti-MAP2 (1:50, #4542, Cell Signaling Technology, RRID:AB_10693782). Donkey anti-mouse IgG Alexa Fluor 488 (A-21202, Thermo Fisher Scientific, Waltham, MA, USA, RRID:AB_141607), goat anti-mouse IgG Alexa Fluor 488 conjugate (A-11029, Thermo Fisher Scientific, RRID:AB_138404), donkey anti-goat IgG Alexa Fluor 633 (A-21082, Thermo Fisher Scientific, RRID:AB_141493), and donkey anti-rabbit IgG Alexa Fluor 546 (A-0040, Thermo Fisher Scientific, RRID:AB_2534016) secondary antibodies were used. Nuclei were stained with DAPI (1:5000, Hoechst) or DRAQ5 (5 mM, BioStatus).

Images were acquired using a confocal Zeiss LSM 710 and a Yokogawa Cell Voyager CV7000 microscope.

### 4.6. Live-Cell Image Acquisition and Analysis

Drug response, when assessing cell viability, was measured with calcein-AM live-cell staining dye (4 mM stock, Invitrogen, Waltham, MA, USA). Briefly, the pillar cells were incubated with calcein-AM dye diluted in MBD-STA500 staining buffer (ratio of 1:8000) (Medical & Bio Device, Suwon, Korea) for 40 min, and then washed in MBD-STA500 buffer without calcein-AM for 15 min at room temperature.

We validated the suitability of calcein-AM as a phenotypical cell viability readout and compared it with a biochemical ATP-based assay. The ATP-based drug response assay was performed according to the manufacturer protocol [43].

The fluorescence imaging of pillar live cells was performed using a Cell Voyager CV7000 (Yokogawa). For quantification of cell viability after exposure to anticancer drugs, all PD-GBOs were 3D-scanned at 4× magnification. Then, 16 bit images were acquired, resulting in one 2560 × 2160 pixel-image per well. Images were analyzed as maximum intensity projections (MIPs). For each MIP, the total surface area *S* representing the vital cells/PD-GBO above the background was calculated. The image analysis was performed on the basis of vital cells per area within the PD-GBO.

To extend the application of this phenotypic drug screening model, we assessed if tumor network complexity could be analyzed and quantified. We, therefore, stained the PD-GBOs with the live-cell membrane probe MemBrite (10 nM, Biotium, Fremont, CA, USA, 30093-T) at 37 °C. Laser scanning microscopy at 37 °C and 5% CO_2_ in a heat-controlled chamber using confocal microscopy (Zeiss LSM 710) was performed. We used semiautomatic quantification of tumor cell numbers and tumor microtubes applying the pixel classification and segmentation workflows of the AIVIA Software 9.8.1 (DRVision Technologies LLC, Bellevue, WA, USA).

For live-cell calcium signaling within cells, the PD-GBOs were loaded with a small molecule calcium indicator, Rhod-2 AM (1 uM, Invitrogen, R-1244), at 37 °C for 30 min. Calcium imaging was performed at 37 °C and 5% CO_2_ in a heat-controlled chamber using confocal microscopy (Zeiss LSM 710, Jena, Germany) and was documented as time-lapse images (scanning speed: 1.52 s per frame). We acquired ImageJ single-cell mean intensity traces over time. These traces were smoothed using the Gaussian filter in MATLAB (MathWorks Inc., Portola Valley, CA, USA), and peaks were detected using the peak finder function.

### 4.7. Analysis of Functional Drug Testing

Each compound was used at seven different molar concentrations: 3 × 10^−5^, 7.5 × 10^−6^, 1.875 × 10^−6^, 4.69 × 10^−7^, 1.17 × 10^−7^, 2.93 × 10^−8^, and 7.32 × 10^−9^. Concentrations presumed to be of clinical relevance were chosen [44].

We established software (DRC Tool) that was used to normalize the data and compute all the dose–response curve (DRC) relative values, including the area under the curve (AUC) (see Supplementary Methods for more details).

### 4.8. RNA-Sequencing Data Processing

RNA-seq was performed on the resected parental glioblastoma tissues. Hg19/GRCh37 was used for alignment.

Raw FastQ data trimming and quality control were performed using TrimGalore (version 0.6.5; https://www.bioinformatics.babraham.ac.uk/projects/trim-galore/ accessed on 30 March 2022). Trimming was conducted using Cutadapt, while FastQC was used for quality control. The alignment of trimmed gene data was performed using Subread package (version 1.6.0; https://sourceforge.net/projects/subread/files/ accessed on 30 March 2022). The reference genome GRCh 37 was downloaded from Broad Institute. ReadCounts were accomplished using the featureCounts function in Subread package (version 1.6.0) (Supplementary Figure S2a).

### 4.9. Pathway Enrichment Analysis

Gene sets of 193 KEGG pathways were downloaded as a gmt file for further analysis. Single-sample gene set enrichment analysis was applied to calculate pathway enrichment levels of each individual patient using GSVA (version 1.38.2). We calculated z-scores of enrichment scores for the four samples to reveal the differences in pathway enrichment patterns among them. For hierarchical clustering and visualization, R package heatmap was used (version 1.0.12; https://www.rdocumentation.org/packages/pheatmap/versions/1.0.12/topics/pheatmap/ accessed on 30 March 2022). For each individual drug, we calculated the linear regression for both biological-related pathways (downloaded from KEGG drug database) and mutation-related pathways.

### 4.10. Correlation Analysis

We calculated Pearson correlation scores between pathway enrichment levels and drug response scores, and then we hierarchically clustered pathway enrichment scores.

## Figures and Tables

**Table 1:** Tumor characteristics.

Patient ID	Weight of Tumor Tissue (g) ^a^	Pattern of Tumor Progression ^b^	Diagnosis	Ki67	Time Until Lab Report (Days)
MA01	9.15	Distant	GB WHO grade 4 MGMT methylated, *IDH* wt	40%	14
MA02	3.42	Distant	GB WHO grade 4 MGMT methylated, *IDH* wt	0%	13
MA03	8.97	Local	GB WHO grade 4 MGMT methylated, *IDH* wt	2%	15
MA04	16.16	Distant	*IDH* mut astrocytoma grade 4	70%	19

^a^ Solid tumor tissue was weighed before processing. ^b^ The patterns of recurrence were categorized as follows: local, ≤20 mm from the resection cavity; distant, >20 mm from the resection cavity; multifocal, multiple foci of tumor progression. Abbreviations: GB, glioblastoma; *IDH*, isocitrate dehydrogenase; MGMT, O^6^-methylguanine-DNA methyltransferase; mut, mutated; WHO, World Health Organization; wt, wildtype.

## Data Availability

The data presented in this study are available on request from the corresponding author. The data are not publicly available doe to national law.

## Supplementary Materials

The following are available online at https://www.mdpi.com/article/10.3390/ijms23126572/s1.
