# Supplementary material for: Patient-Derived Tumor Organoids for Guidance of Personalized Drug Therapies in Recurrent Glioblastoma
Source: Int J Mol Sci. 2022 Jun 12;23(12):6572. doi: 10.3390/ijms23126572 (PMC9223608; doi:10.3390/ijms23126572)
Supplement: Supplementary file 1 [file ijms-23-06572-s001.zip › Supplementary materials.pdf]

Supplementary materials for

Patient-derived tumor organoids for guidance of personalized drug therapies in recurrent glioblastoma

Miriam Ratliff [1,2,*], Hichul Kim [3,4], Hao Qi [5], Minsung Kim [6], Bosung Ku [7], Daniel Dominguez Azorin [2,8], David Hausmann [2,8], Rajiv K. Khajuria [1,2,8], Areeba Patel [9], Elena Maier [1], Loic Cousin [4], Arnaud Ogier [4], Felix Sahm [9], Nima Etminan [1], Lukas Bunse [5,10], Frank Winkler [2,8], Victoria El-Khoury [3,11], Michael Platten [5,10,12] and Yong-Jun Kwon [3,4,*]

* Correspondence: miriam.ratliff@umm.de; yjkwonipk@gmail.com

This file includes:

Supplementary methods on the analysis of functional drug testing

Figure S1. Cell viability curves of identified drug candidates for patients MA01-MA04.

Figure S2. Expression analysis and intra-sample pathway enrichment in glioblastoma tumor tissue.

Figure S3. Correlation of AUCs derived from dose response curves of two independent assays.

Other supplementary materials for this manuscript include the following:

Movie S1. Life-cell calcium imaging shows partially synchronized tumor cell communication via a functional tumor cell network.

Table S1. Mean area-under-the-curve values for all tested drugs in the individual patients MA01-MA04 and their corresponding mean z-score. The color-coding of the drugs is analogous to the colors used to group drugs in Figure 4. Abbreviation: SD, standard deviation.

Table S2. Raw values of inter-individual enrichment of KEGG pathways from RNA-seq of glioblastoma tumor tissue.

Table S3. Pearson correlation coefficients of PD-GBO drug response score and specific inter-individual KEGG pathway enrichment levels from RNA-seq of glioblastoma tumor tissue.

Table S4. AUCs from a comparative response analysis of calcein-AM- and ATP-based measurements.

Supplementary methods on the analysis of functional drug testing:

All characteristics (cell surface, cell number, average fluorescence intensity) were normalized plate by plate, using the average values of the corresponding DMSO vehicle control samples. We defined the average value of the vehicle control using:

$$\mu_{DMSO} = \sum_{i=1}^{N} y_{i,DMSO}$$

Where i represented the different vehicle control samples of each plate. For each compound, the descriptor value y_i was normalized for each concentration i as follows:

$$y_{norm,i} = \frac{y_i}{\mu_{DMSO}}$$

Extreme values were considered as outliers and were removed from the average. The compound efficiency was quantified as the AUC. The AUC was computed using the sum of the trapeze surface between each concentration as:

$$AUC = \sum_{i=0}^{N-1} (x_{i+1} - x_i) \times \frac{1}{2}\left(\frac{1}{P}\sum_{j=1}^{P} y_{norm,i+1,j} + \frac{1}{P}\sum_{j=4}^{P} y_{norm,i,j}\right)$$

Where x_i was the concentration i and $y_{norm,i,j}$ the correspondent normed descriptor value for the experiment number j over the P replicate experiments. As we had P experiments, we could compute the statistical error of the AUC value. For each compound, the variance on the AUC was defined as the following:

$$V(AUC) = \left(\frac{\partial AUC}{\partial y_i}\right)^T \Sigma \left(\frac{\partial AUC}{\partial y_i}\right)$$

Where Σ was the variance-covariance matrix and i the index of the concentration. As samples were independent, Σ is diagonal.

$$\Delta AUC = \sqrt{\sum_{i=1}^{N-1} \left(\frac{\partial AUC}{\partial y_i}\right)^2 \sigma_i^2} = \sqrt{\sum_{i=1}^{N-1} \left(\frac{x_{i+1} - x_{i-1}}{2}\right)^2 \sigma_i^2}$$

Assumed:

$$\mu_M = \frac{1}{N}\sum_{i} y_{norm,i}$$

$$\sigma_i^2 = \frac{1}{N}\sum_{i}^{N} \left(y_{norm,i} - \mu_M\right)^2$$

Finally, a 95% confidence interval was provided using the z-distribution in the following

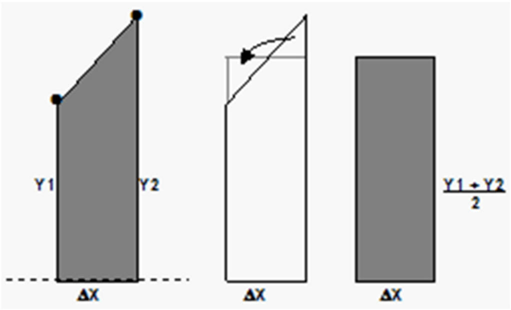

way:

$$\Delta AUC = 1.96 \times \sqrt{\sum_i \left(\frac{x_{i+1} - x_{i-1}}{2}\right)^2 \sigma_i^2}$$

In order to compare the different AUC values of each compound k, we used a z-score predefined as:

$$\mu_{AUC} = \frac{1}{N}\sum_k AUC_k$$

$$\sigma_{AUC} = \sqrt{\frac{1}{N}\sum_k (AUC_k - \mu_{AUC})^2}$$

$$Z_{AUC,i} = \frac{AUC_i - \mu_{AUC}}{\sigma_{AUC}}$$

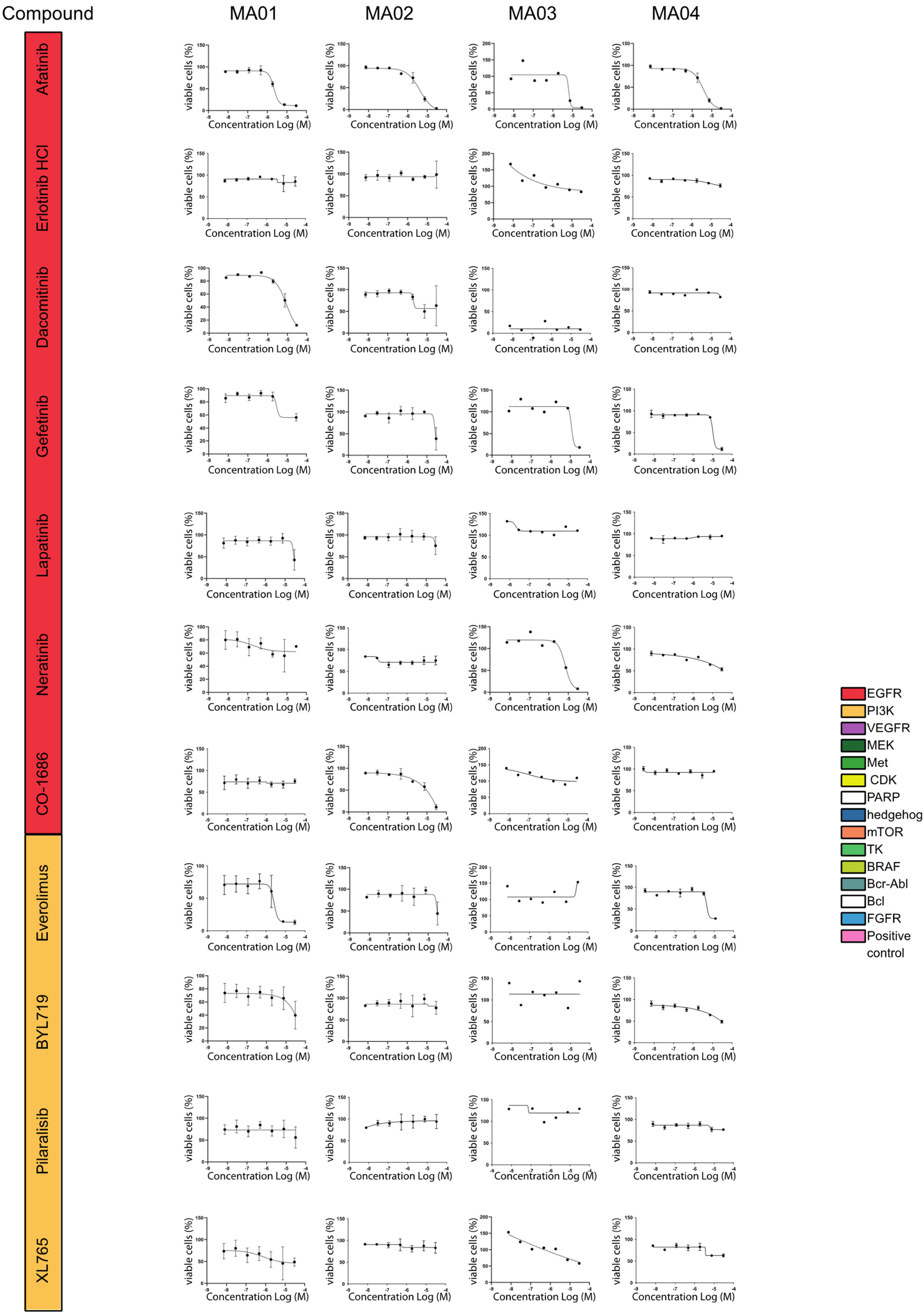

Compound
MA01
MA02
MA03
MA04
Afatinib
Erlotinib HCl
Dacomitinib
Gefetinib
Lapatinib
Neratinib
CO-1686
Everolimus
BYL719
Pilaralisib
XL765
viable cells (%)
Concentration Log (M)
EGFR
PI3K
VEGFR
MEK
Met
CDK
PARP
hedgehog
mTOR
TK
BRAF
Bcr-Abl
Bcl
FGFR
Positive control

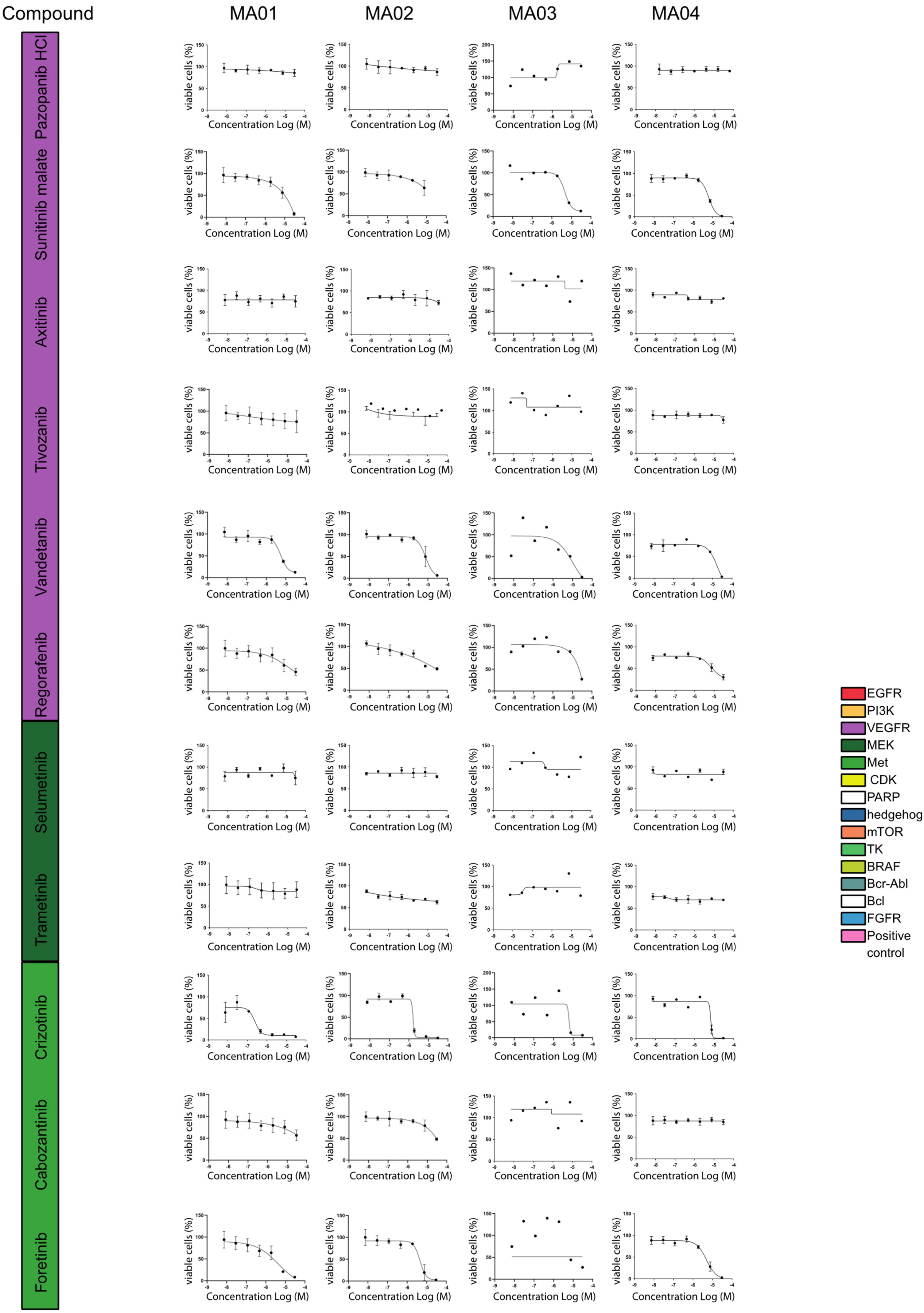
Compound
MA01
MA02
MA03
MA04
Pazopanib HCl
Sunitinib malate
Axitinib
Tivozanib
Vandetanib
Regorafenib
Selumetinib
Trametinib
Crizotinib
Cabozantinib
Foretinib
viable cells (%)
Concentration Log (M)
EGFR
PI3K
VEGFR
MEK
Met
CDK
PARP
hedgehog
mTOR
TK
BRAF
Bcr-Abl
Bcl
FGFR
Positive control

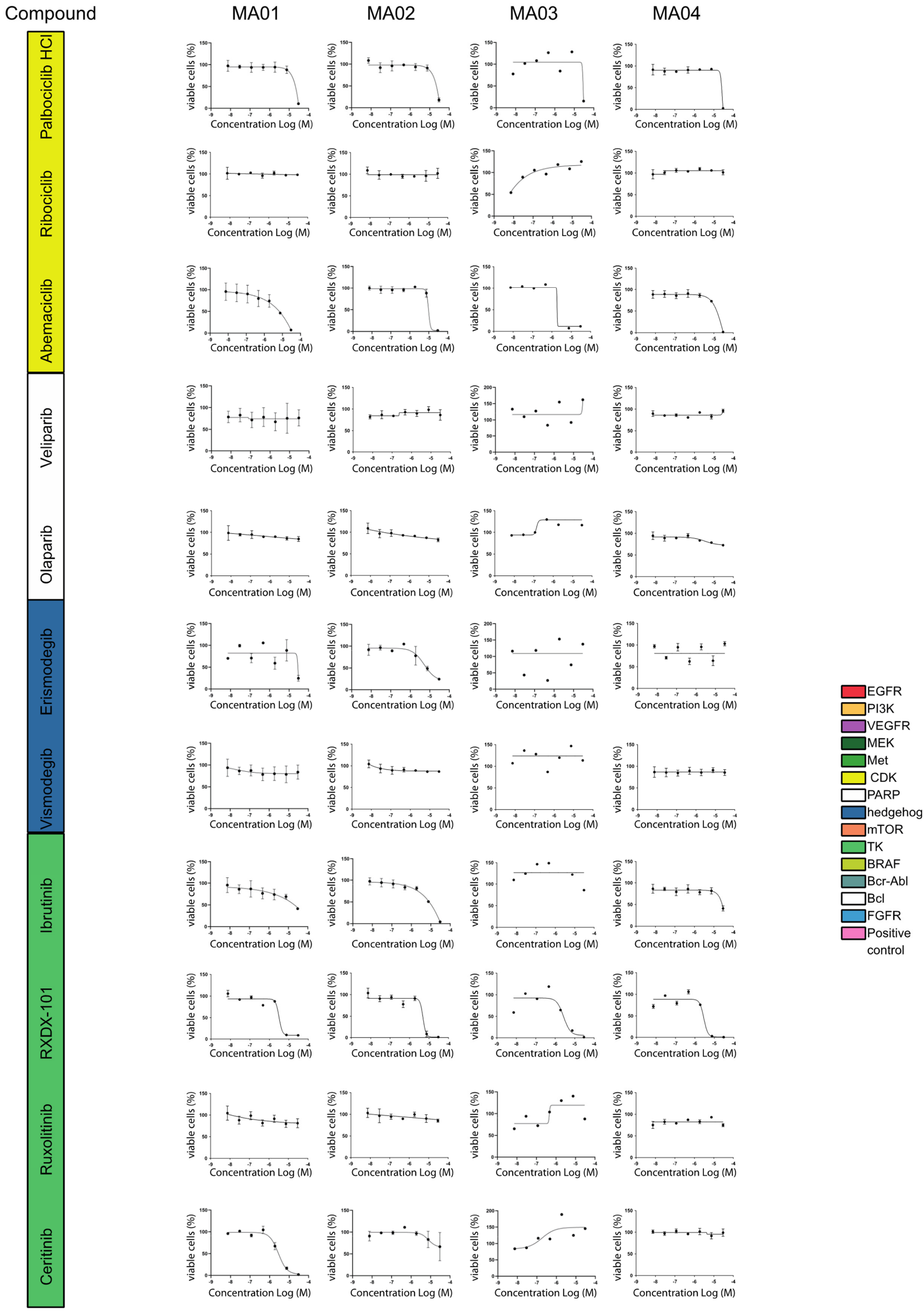

Compound
MA01
MA02
MA03
MA04
Palbociclib HCl
Ribociclib
Abemaciclib
Veliparib
Olaparib
Erismodegib
Vismodegib
Ibrutinib
RXDX-101
Ruxolitinib
Ceritinib
viable cells (%)
Concentration Log (M)
EGFR
PI3K
VEGFR
MEK
Met
CDK
PARP
hedgehog
mTOR
TK
BRAF
Bcr-Abl
Bcl
FGFR
Positive control

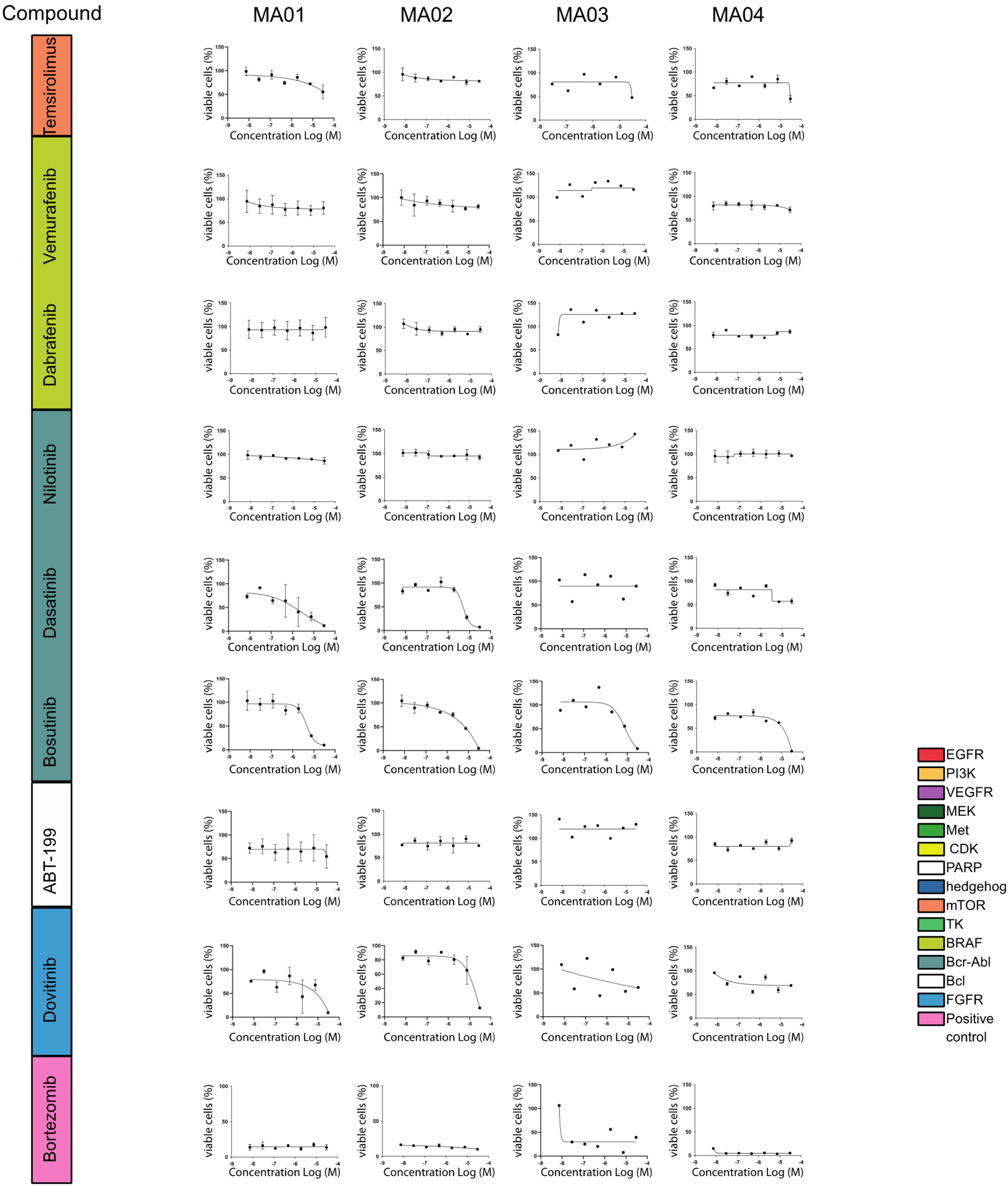

Figure S1. Cell viability curves of all compounds for patients MA01-MA04.

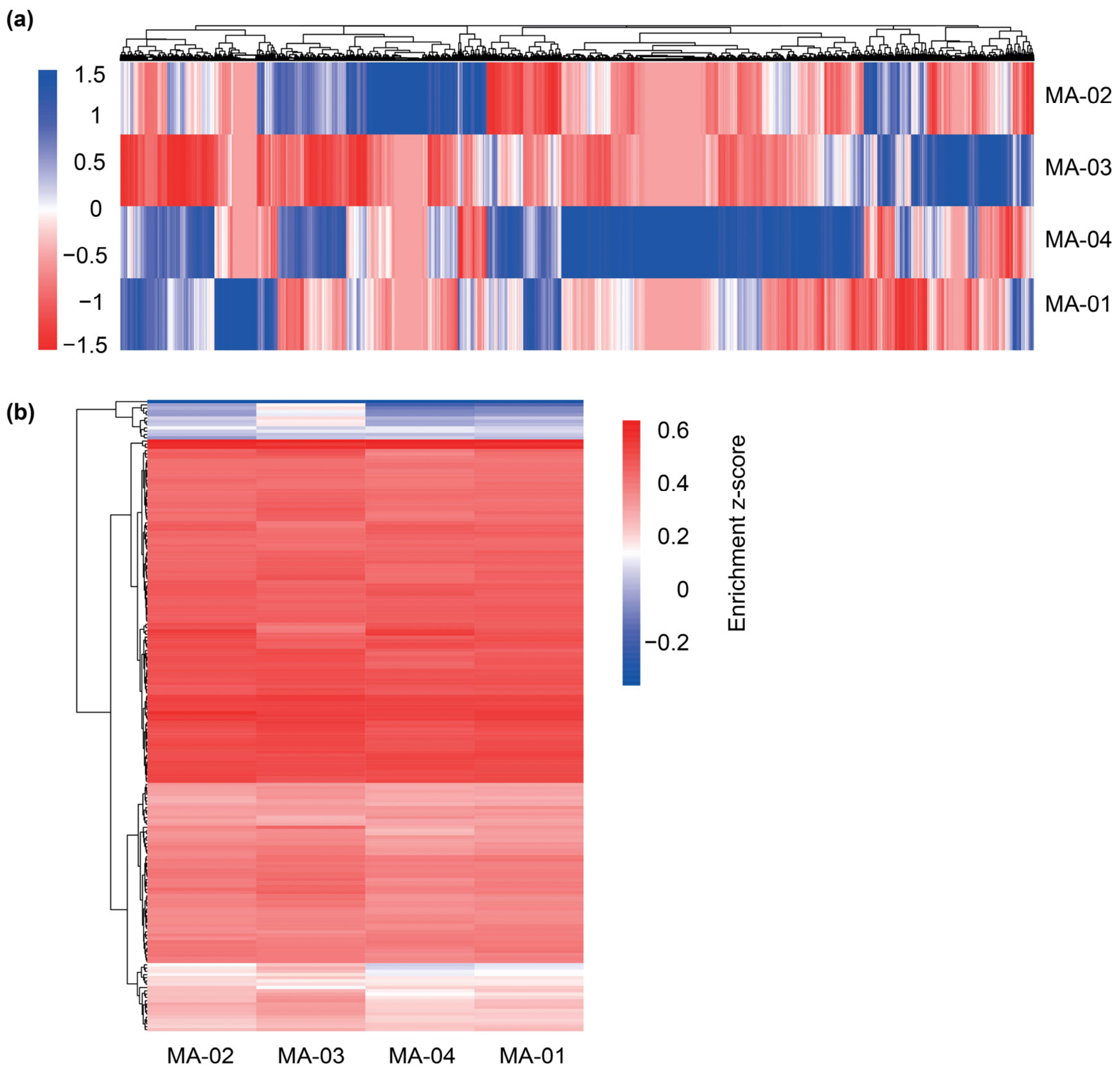

Figure S2. Expression analysis and intra-sample pathway enrichment in glioblastoma tumor tissue.

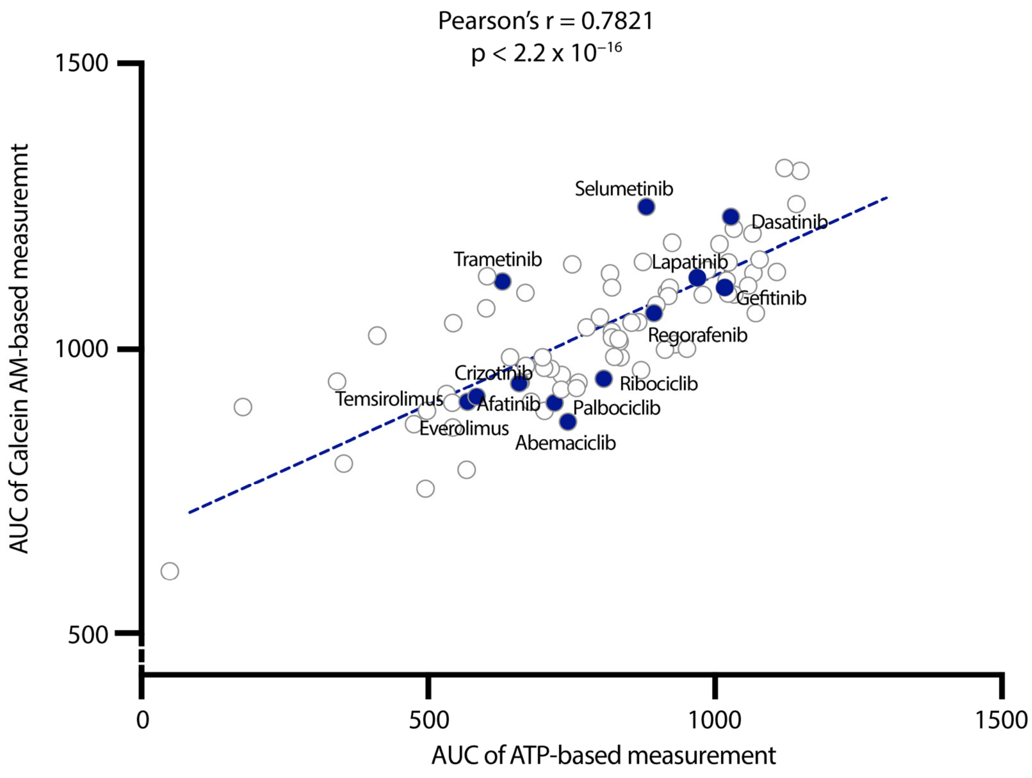

Figure S3. Correlation of AUCs derived from dose response curves of two independent assays.
