# Supplementary material for: Patient-Derived Tumor Organoids for Guidance of Personalized Drug Therapies in Recurrent Glioblastoma
Source: Int J Mol Sci. 2022 Jun 12;23(12):6572. doi: 10.3390/ijms23126572 (PMC9223608; doi:10.3390/ijms23126572)
Supplement: Supplementary file 1 [file ijms-23-06572-s001.zip › Table S2.pdf]

Table S2. Raw values of inter-individual enrichment of KEGG pathways from RNA-seq of glioblastoma tumor tissue.

	Pathways	cluster	Afatinib	Erlotinib HCl	Dacomitinib	Gefitinib	Lapatinib	Neratinib	CO-1686	Everolimus	BYL719	Pilaralisib	XL765	Pazopanib HCl	Sunitinib Malate
1	ABC_TRANSPORTERS	C3	0.042868983	0.344133884	-0.603075154	0.236562809	-0.285508155	-0.228591303	-0.420868494	-0.224758684	-0.098339820	-0.091118024	-0.169234872	0.172645329	0.346217840
2	ACUTE_MYELOID_LEUKEMIA	C3	0.419581299	0.047191204	0.390363130	-0.322163112	-0.091031985	0.508968870	0.666596482	-0.072568705	-0.165894137	0.060900321	-0.196497087	0.071498833	0.341592876
3	ADHERENS_JUNCTION	C3	0.474243727	0.158037622	0.317387501	-0.232366659	-0.095363754	0.510886322	0.624126508	-0.064732780	-0.134536716	0.094838362	-0.179123535	0.153216675	0.449462540
4	ADIPOCYTOKINE_SIGNALING_PATHWAY	C2	0.772671654	0.557024690	0.462027394	0.193483353	0.238367688	0.737196876	0.733448072	0.282494063	0.245681926	0.453788667	0.188231264	0.542590216	0.782399346
5	ALANINE_ASPARTATE_AND_GLUTAMATE_METABOLISM	C3	-0.058882281	0.130742145	0.208176869	0.495489232	0.551890470	-0.029296570	-0.140159183	0.514176007	0.538308072	0.345320966	0.591758018	0.206981387	-0.151337659
6	ALDOSTERONE_REGULATED_SODIUM_REABSORPTION	C3	0.285791785	-0.159158858	0.518525987	-0.443002115	-0.042950288	0.472178206	0.697948747	-0.051183227	-0.182161715	0.013789831	-0.180879849	-0.076028218	0.105323423
7	ALLOGRAFT_REJECTION	C2	0.926230274	0.667691268	0.824789810	0.408531164	0.622741605	0.964348554	0.957607794	0.645112824	0.578191163	0.747572158	0.548978803	0.728099006	0.819087925
8	ALPHA_LINOLENIC_ACID_METABOLISM	C2	0.564065383	0.535132780	0.795416256	0.700778594	0.942231961	0.640714931	0.553190646	0.917687540	0.883295405	0.821741451	0.918389394	0.659951594	0.381422052
9	ALZHEIMERS_DISEASE	C2	0.906597637	0.760379198	0.556891748	0.453448099	0.462902223	0.841281883	0.769471635	0.508737741	0.490076574	0.665026235	0.433124156	0.750969886	0.920783125
10	AMINO_SUGAR_AND_NUCLEOTIDE_SUGAR_METABOLISM	C2	0.774776848	0.641230742	0.354433970	0.290870306	0.231212303	0.685062033	0.630120950	0.284414266	0.276610426	0.467255060	0.209490004	0.598080590	0.839423622
11	AMINOACYL_TRNA_BIOSYNTHESIS	C3	0.185894403	0.574063552	-0.081360089	0.814211967	0.496964455	0.004325959	-0.272195494	0.511662766	0.625190122	0.464958562	0.617354093	0.528916207	0.310772556
12	AMYOTROPHIC_LATERAL_SCLEROSIS_ALS	C3	0.121246345	-0.117696777	-0.092085133	-0.493267102	-0.483872433	0.120642643	0.250648163	-0.450197673	-0.491629154	-0.285764023	-0.540659479	-0.173481494	0.177170109
13	ANTIGEN_PROCESSING_AND_PRESENTATION	C2	0.659570418	0.328646760	0.540207967	-0.037815833	0.158251837	0.711304144	0.799393621	0.185366843	0.106244730	0.329948709	0.067145603	0.353627508	0.591218328
14	APOPTOSIS	C3	0.556390051	0.171606633	0.560063721	-0.178248450	0.100314642	0.658871703	0.798852448	0.115656169	0.014644638	0.236639513	-0.011262807	0.219362357	0.443818879
15	ARACHIDONIC_ACID_METABOLISM	C3	0.490059609	0.081789601	0.561590783	-0.253247525	0.066277915	0.618891351	0.785308268	0.074871175	-0.036579388	0.180875915	-0.054786578	0.141306079	0.354954011
16	ARGININE_AND_PROLINE_METABOLISM	C2	0.865614577	0.557683965	0.828120721	0.284567751	0.550944106	0.935734281	0.970730805	0.568047942	0.484684115	0.666303869	0.460623523	0.628534737	0.734611052
17	ARRHYTHMOGENIC_RIGHT_VENTRICULAR_CARDIOMYOPATHY_ARVC	C2	0.760293244	0.473219090	0.572706428	0.115386623	0.265881795	0.780223463	0.822721413	0.298993758	0.234770550	0.450103521	0.189710412	0.490579453	0.711602336
18	ASCORBATE_AND_ALDARATE_METABOLISM	C3	0.485700081	0.062094844	0.617523406	-0.249764898	0.112213352	0.636507152	0.812882701	0.114624480	-0.005535688	0.203488898	-0.016135172	0.137973792	0.323437601
19	ASTHMA	C2	0.879565680	0.623526058	0.694618275	0.308800426	0.464880469	0.892158700	0.893131113	0.496530100	0.437892288	0.631332055	0.396363128	0.655148825	0.815765873
20	AUTOIMMUNE_THYROID_DISEASE	C2	0.671516657	0.338287854	0.560164546	-0.024381027	0.179465060	0.726244588	0.813520557	0.205777500	0.125257002	0.347765361	0.087225039	0.366752220	0.597785858
21	AXON_GUIDANCE	C3	0.119486092	-0.077171690	-0.157587743	-0.450900962	-0.500093591	0.088789994	0.193013551	-0.460930309	-0.486772090	-0.287705139	-0.541860464	-0.150122945	0.207268186
22	B_CELL_RECEPTOR_SIGNALING_PATHWAY	C3	0.571795896	0.261150853	0.400714687	-0.125091214	0.016424002	0.602774915	0.694676604	0.048498014	-0.020143295	0.208206177	-0.065779267	0.262685723	0.540123809
23	BASAL_CELL_CARCINOMA	C3	0.279554850	0.538762670	0.273294491	0.823697588	0.738704350	0.209610012	-0.005089457	0.729217830	0.788354561	0.627488789	0.809286023	0.574360505	0.265754314
24	BASAL_TRANSCRIPTION_FACTORS	C3	-0.015844945	0.219137746	0.166082450	0.578484355	0.569322897	-0.023510568	-0.170156811	0.539412752	0.581043133	0.384864191	0.625818867	0.276883781	-0.073047003
25	BASE_EXCISION_REPAIR	C3	-0.089949692	0.150817965	0.109891979	0.521944960	0.508694232	-0.093830106	-0.230632160	0.476486494	0.519051385	0.315471747	0.566441705	0.206082771	-0.144166804
26	BETA_ALANINE_METABOLISM	C1	-0.982523203	-0.873505590	-0.676162120	-0.639146597	-0.668333264	-0.920348238	-0.810764659	-0.707845609	-0.693445980	-0.831547132	-0.646194331	-0.885662638	-0.970333286
27	BIOSYNTHESIS_OF_UNSATURATED_FATTY_ACIDS	C3	0.205030796	0.582275786	-0.039602573	0.827437781	0.531963576	0.033074149	-0.240276519	0.544562682	0.653281590	0.492631793	0.648026501	0.545294062	0.316373954
28	BLADDER_CANCER	C3	0.365276644	-0.084471816	0.719212365	-0.287943332	0.198932555	0.589021989	0.802171237	0.177287382	0.033837580	0.195357489	0.052977585	0.041638169	0.120159615
29	BUTANOATE_METABOLISM	C1	-0.443787620	-0.001407984	-0.680065283	0.264988348	-0.161238507	-0.630605066	-0.825647585	-0.152605284	-0.019721237	-0.210060503	-0.022695181	-0.102258863	-0.239945531
30	CALCIUM_SIGNALING_PATHWAY	C3	-0.234053635	-0.304455044	-0.537976297	-0.593624390	-0.776809089	-0.306065024	-0.216090221	-0.739900735	-0.728030729	-0.590973658	-0.779425587	-0.418421674	-0.074236793
31	CARDIAC_MUSCLE_CONTRACTION	C1	-0.402876531	0.015562857	-0.508489523	0.345740348	0.015068376	-0.544496904	-0.733080870	0.009964288	0.125505676	-0.089705678	0.140051071	-0.044242433	-0.264109248
32	CELL_ADHESION_MOLECULES_CAMS	C3	0.399944680	0.072533791	0.273945709	-0.315161207	-0.166351476	0.448692870	0.582408149	-0.138796636	-0.212851776	0.015616548	-0.254249770	0.068400603	0.371028482
33	CELL_CYCLE	C1	-0.407985288	-0.156822021	-0.144683902	0.241249307	0.209523515	-0.395819783	-0.481169088	0.169545005	0.213766675	-0.008842325	0.269292765	-0.116500139	-0.444756540
34	CHEMOKINE_SIGNALING_PATHWAY	C3	0.442831529	0.025575670	0.543698524	-0.303319428	0.030372425	0.582741492	0.763265994	0.035989624	-0.079430465	0.135705869	-0.094332401	0.088260301	0.300673464
35	CHRONIC_MYELOID_LEUKEMIA	C3	0.360103584	-0.091521384	0.656170140	-0.335067682	0.117634940	0.567118423	0.782886572	0.102581480	-0.036381618	0.143485575	-0.025889725	0.017388309	0.140919818
36	CIRCADIAN_RHYTHM_MAMMAL	C2	0.895154216	0.634962580	0.737075017	0.334713010	0.511217164	0.916840657	0.917334813	0.540015814	0.477896685	0.665617627	0.440074046	0.675804247	0.817038863
37	CITRATE_CYCLE_TCA_CYCLE	C2	0.512404068	0.728208996	0.424384153	0.933544295	0.858594758	0.429620062	0.200093933	0.860206667	0.912461473	0.797698378	0.919490832	0.763521489	0.498206843
38	COLORECTAL_CANCER	C3	0.306254522	0.040986490	0.835642793	0.137221970	0.628750717	0.533675611	0.639995846	0.579917889	0.467064564	0.465807861	0.525845457	0.216887792	0.008423073
39	COMPLEMENT_AND_COAGULATION_CASCADES	C3	0.500380682	0.153367605	0.406043437	-0.225928700	-0.032739025	0.563740758	0.691055337	-0.007902328	-0.090766856	0.138334541	-0.128433277	0.168280570	0.441871103
40	CYSTEINE_AND_METHIONINE_METABOLISM	C3	-0.012082659	0.383691353	-0.170689480	0.686865228	0.398496778	-0.163382862	-0.409168199	0.400522158	0.509539649	0.316331002	0.516977026	0.349109033	0.093435902
41	CYTOKINE_CYTOKINE_RECEPTOR_INTERACTION	C3	0.543161950	0.133824003	0.628342984	-0.189214936	0.147423163	0.676354584	0.833556681	0.154555487	0.040950469	0.254009149	0.024985640	0.202810282	0.394570164
42	CYTOSOLIC_DNA_SENSING_PATHWAY	C3	0.549277286	0.278432062	0.305426492	-0.116055479	-0.040589989	0.548124137	0.619705845	-0.001924302	-0.054523836	0.171600345	-0.107860124	0.257950424	0.556102497
43	DILATED_CARDIOMYOPATHY	C2	0.671583858	0.289547706	0.705959443	-0.028353629	0.285514210	0.782518482	0.899310300	0.297360947	0.192806040	0.401865644	0.172315839	0.358315017	0.529717473
44	DNA_REPLICATION	C1	-0.244269266	0.004554813	-0.010714628	0.393762096	0.371810255	-0.240286817	-0.354044723	0.335382341	0.379232442	0.163726613	0.431231822	0.053499302	-0.291040805
45	DORSO_VENTRAL_AXIS_FORMATION	C3	-0.060558919	-0.258899083	-0.268931884	-0.603796261	-0.640364346	-0.070426622	0.067418202	-0.608990117	-0.638476461	-0.455277277	-0.684749134	-0.331141961	0.022072825
46	DRUG_METABOLISM_CYTOCHROME_P450	C3	0.478976598	0.095103358	0.475596405	-0.264533148	-0.002998618	0.579093769	0.732807867	0.013051338	-0.086055683	0.138943598	-0.113402096	0.132718225	0.380377534
47	DRUG_METABOLISM_OTHER_ENZYMES	C3	0.442562885	0.056888420	0.445474855	-0.303378452	-0.043080311	0.545014259	0.706512259	-0.027690670	-0.127248020	0.097676921	-0.154037704	0.092421034	0.346129073
48	ECM_RECEPTOR_INTERACTION	C2	0.843566495	0.519682756	0.839799710	0.250387703	0.540956070	0.927912167	0.976734583	0.554884114	0.465145039	0.646899703	0.444646723	0.597396265	0.699468464
49	ENDOCYTOSIS	C3	0.178578537	-0.087501004	-0.008229159	-0.469188381	-0.421699877	0.193024855	0.329352376	-0.389890684	-0.440909807	-0.227498035	-0.487811242	-0.130480641	0.212522663
50	ENDOMETRIAL_CANCER	C1	-0.429326672	-0.321450729	-0.813398569	-0.494735688	-0.843400950	-0.572145386	-0.557874624	-0.804721898	-0.740335764	-0.684127915	-0.790824542	-0.473466155	-0.191677945
51	EPITHELIAL_CELL_SIGNALING_IN_HELICOBACTER_PYLORI_INFECTION	C3	0.606237023	0.314948647	0.399285058	-0.072027955	0.044357173	0.621461054	0.695129676	0.079720297	0.018281098	0.244827092	-0.030890296	0.310414707	0.587165245
52	ERBB_SIGNALING_PATHWAY	C1	-0.557860851	-0.548823463	-0.771939449	-0.723203565	-0.943758740	-0.624135274	-0.525363586	-0.920360247	-0.892309290	-0.823898696	-0.926381484	-0.668647668	-0.385380208
53	ERBB_SIGNALING_PATHWAY.1	C1	-0.469408192	-0.567816372	-0.607447756	-0.796265575	-0.905356672	-0.484763774	-0.335217656	-0.886168921	-0.891688851	-0.782195453	-0.922427777	-0.659830555	-0.351917683
54	ETHER_LIPID_METABOLISM	C3	0.287971846	-0.133703080	0.421215909	-0.458278292	-0.128095727	0.438628127	0.650166918	-0.126049342	-0.243074864	-0.031883815	-0.254535203	-0.078162735	0.152839649
55	FATTY_ACID_METABOLISM	C3	0.233937608	0.081621181	-0.135907825	-0.297892288	-0.405227117	0.164007765	0.220008777	-0.357312041	-0.366265484	-0.168871617	-0.430785139	-0.004531463	0.350271461
56	FC_EPSILON_RI_SIGNALING_PATHWAY	C2	0.747746516	0.389721448	0.747080022	0.079716720	0.373369030	0.841962512	0.929903332	0.388252160	0.291364543	0.494756195	0.268078730	0.457190943	0.612699554
57	FC_GAMMA_R_MEDIATED_PHAGOCYTOSIS	C3	0.461883986	0.165478591	0.268832117	-0.229114228	-0.125152365	0.482542513	0.585886153	-0.091243605	-0.153050981	0.075236686	-0.201474756	0.149584799	0.456413445
58	FOCAL_ADHESION	C2	0.745898375	0.402796793	0.690616680	0.072401647	0.326166901	0.819934771	0.899271318	0.346605032	0.257937785	0.468449753	0.227804995	0.454866236	0.636349490

	Pathways	Axitinib	Tivozanib	Vandetanib	Regorafenib	Selumetinib	Trametinib	Crizotinib	Cabozantinib	Foretinib	Palbociclib HCl	Ribociclib	Abemaciclib	Veliparib	Olaparib
1	ABC_TRANSPORTERS	−0.010346404	0.034854692	0.505100234	0.585858472	0.403344917	0.654047231	−0.520023335	−0.107184028	−0.071548389	0.323485205	−0.896810476	−0.077810526	−0.189534280	0.410893986
2	ACUTE_MYELOID_LEUKEMIA	0.426635480	0.106477962	0.087362677	0.245993823	0.439336854	0.620158360	0.335864328	0.407353109	0.304776259	0.372971379	0.074140309	−0.248707425	0.199169175	0.278773462
3	ADHERENS_JUNCTION	0.471555828	0.163587051	0.223593950	0.392146655	0.553739127	0.756628986	0.282038481	0.434820970	0.340638159	0.476497579	−0.112914470	−0.214582459	0.213831415	0.398117844
4	ADIPOCYTOKINE_SIGNALING_PATHWAY	0.761793764	0.534043922	0.608124448	0.728960125	0.851298835	0.884085798	0.475140666	0.716709985	0.655716755	0.800608203	−0.416329507	0.172553071	0.530693232	0.745845881
5	ALANINE_ASPARTATE_AND_GLUTAMATE_METABOLISM	−0.039730486	0.246277132	−0.009646047	−0.220958994	−0.291569365	−0.736250258	0.244017911	0.025149121	0.111043795	−0.169244048	0.201345655	0.594025427	0.262370215	−0.129728541
6	ALDOSTERONE_REGULATED_SODIUM_REABSORPTION	0.312192110	0.006200953	−0.178609439	−0.061773721	0.175892562	0.275903760	0.433054618	0.331426505	0.224465615	0.141950797	0.439057132	−0.261707586	0.175197201	0.025809045
7	ALLOGRAFT_REJECTION	0.934926540	0.770378591	0.626299039	0.659747098	0.826302380	0.599848395	0.836522540	0.932960155	0.887189508	0.839364134	−0.199824044	0.507277611	0.830461468	0.766985951
8	ALPHA_LINOLENIC_ACID_METABOLISM	0.591984723	0.736188456	0.366006624	0.190130886	0.254342898	−0.322072357	0.828246237	0.658010677	0.701786388	0.380866834	0.128444711	0.884277979	0.811148231	0.360222121
9	ALZHEIMERS_DISEASE	0.894278615	0.738543232	0.787429370	0.860219043	0.956034575	0.854557354	0.596984206	0.853262833	0.818140275	0.932737362	−0.533327792	0.426828143	0.712370357	0.893636870
10	AMINO_SUGAR_AND_NUCLEOTIDE_SUGAR_METABOLISM	0.754562735	0.564333902	0.712572652	0.829797119	0.906688737	0.949232358	0.388476763	0.695431935	0.650340211	0.851086571	−0.585030574	0.213305262	0.515298874	0.818634309
11	AMINOACYL_TRNA_BIOSYNTHESIS	0.164777724	0.467849542	0.546797514	0.395840899	0.210029392	−0.110066049	0.027307971	0.154174804	0.264060264	0.277834306	−0.564769968	0.684855533	0.315460257	0.374583974
12	AMYOTROPHIC_LATERAL_SCLEROSIS_ALS	0.107759779	−0.196618287	0.005118076	0.212575356	0.313429148	0.720934260	−0.135471883	0.051686994	−0.041617408	0.198880624	−0.129646716	−0.554842060	−0.186651085	0.145364005
13	ANTIGEN_PROCESSING_AND_PRESENTATION	0.663001724	0.381836002	0.354562146	0.484239916	0.666484156	0.723897543	0.514994877	0.640115003	0.554720582	0.618746993	−0.110267821	0.023083758	0.448853287	0.533294991
14	APOPTOSIS	0.568390331	0.268595710	0.180930423	0.309620300	0.519289919	0.589400949	0.512613993	0.560203491	0.464140415	0.475665353	0.082727634	−0.070455651	0.375270480	0.375751979
15	ARACHIDONIC_ACID_METABOLISM	0.506589705	0.201328070	0.081857623	0.207973414	0.429358684	0.505575260	0.502610019	0.506635545	0.406223993	0.388668202	0.188008124	−0.121364579	0.328154868	0.282398350
16	ARGININE_AND_PROLINE_METABOLISM	0.878799557	0.682425259	0.514240263	0.560990606	0.751158218	0.561187591	0.822770316	0.882364494	0.823676416	0.759021574	−0.085483610	0.408519201	0.768111962	0.674113218
17	ARRHYTHMOGENIC_RIGHT_VENTRICULAR_CARDIOMYOPATHY_ARVC	0.759582355	0.508751385	0.497904880	0.612351765	0.775620627	0.779992716	0.566401968	0.731579741	0.658982231	0.735380420	−0.236371330	0.154942817	0.551431410	0.660672408
18	ASCORBATE_AND_ALDARATE_METABOLISM	0.507102701	0.210207911	0.043926240	0.155035693	0.387592198	0.425635933	0.553746654	0.517254281	0.417681061	0.358158447	0.264486191	−0.089641840	0.354889813	0.246789780
19	ASTHMA	0.880787691	0.678638353	0.620486566	0.694893115	0.851363188	0.735617031	0.704604875	0.860995273	0.805138828	0.836448074	−0.281597996	0.362938199	0.717268222	0.767400565
20	AUTOIMMUNE_THYROID_DISEASE	0.675800554	0.397261740	0.359875446	0.485546012	0.670151968	0.714293734	0.535206160	0.654766847	0.570059811	0.625432690	−0.103143046	0.041966290	0.467010228	0.539061876
21	AXON_GUIDANCE	0.100563738	−0.187794166	0.059261158	0.268330244	0.345073758	0.766643139	−0.190305303	0.035742847	−0.050253623	0.225657690	−0.223809632	−0.545335688	−0.203136506	0.183851724
22	B_CELL_RECEPTOR_SIGNALING_PATHWAY	0.569512165	0.275206322	0.315306105	0.469523334	0.633411598	0.777411428	0.374305146	0.534665209	0.446034326	0.566451516	−0.156648411	−0.101232539	0.323039167	0.488371793
23	BASAL_CELL_CARCINOMA	0.281792120	0.575220107	0.430822971	0.232259826	0.130190752	−0.358145926	0.355173278	0.314427083	0.413426357	0.242287799	−0.226528999	0.836790838	0.517848064	0.300419109
24	BASAL_TRANSCRIPTION_FACTORS	−0.003138824	0.299726842	0.092628420	−0.117851747	−0.212845935	−0.656540581	0.217037154	0.051047759	0.145469747	−0.094559315	0.070393998	0.640116995	0.285763419	−0.042702606
25	BASE_EXCISION_REPAIR	−0.077049828	0.228177043	0.027717107	−0.180692262	−0.281483190	−0.699172138	0.156287197	−0.022083729	0.072113838	−0.166056563	0.105734839	0.581385687	0.215314949	−0.112212503
26	BETA_ALANINE_METABOLISM	−0.974655972	−0.883837957	−0.862660346	−0.882551532	−0.969124558	−0.729819945	−0.730084046	−0.948352045	−0.933801618	−0.978554484	0.526093339	−0.639411456	−0.860738746	−0.946191655
27	BIOSYNTHESIS_OF_UNSATURATED_FATTY_ACIDS	0.186115422	0.490167539	0.546216829	0.389074846	0.211070092	−0.131394949	0.067642241	0.179750410	0.289358994	0.284200264	−0.538404310	0.711879290	0.346211689	0.377507214
28	BLADDER_CANCER	0.402724068	0.151821283	−0.161140313	−0.105100060	0.148447442	0.081160837	0.636897997	0.448232317	0.356149821	0.156263355	0.555420091	−0.040236524	0.352512703	0.034841343
29	BUTANOATE_METABOLISM	−0.473019737	−0.193440824	0.044934217	−0.041542666	−0.287819661	−0.273933440	−0.607180214	−0.499876667	−0.402485649	−0.275836739	−0.401268227	0.061174791	−0.366370290	−0.157983402
30	CALCIUM_SIGNALING_PATHWAY	−0.261746881	−0.486300615	−0.134428937	0.072596818	0.068352446	0.608298057	−0.567775294	−0.336086799	−0.402210304	−0.065618022	−0.235740046	−0.757994857	−0.546731105	−0.071581858
31	CARDIAC_MUSCLE_CONTRACTION	−0.420795801	−0.106741453	0.012072758	−0.121129503	−0.344407763	−0.458398316	−0.439992414	−0.422821810	−0.317904579	−0.298786980	−0.253670541	0.209090296	−0.242031202	−0.190476312
32	CELL_ADHESION_MOLECULES_CAMS	0.398603656	0.081358340	0.140968820	0.316310620	0.480809382	0.717094446	0.229867770	0.363689799	0.265019535	0.399095901	−0.053500960	−0.292276615	0.139855306	0.318325478
33	CELL_CYCLE	−0.395230582	−0.099510196	−0.256519304	−0.442020639	−0.562079071	−0.838040660	−0.121027205	−0.340720828	−0.253564155	−0.466929049	0.250847083	0.286339464	−0.107343187	−0.407671096
34	CHEMOKINE_SIGNALING_PATHWAY	0.461010590	0.151903334	0.024266612	0.151797658	0.376595557	0.466691570	0.478591627	0.463924859	0.361032590	0.335150829	0.237577716	−0.163597374	0.286724574	0.226674792
35	CHRONIC_MYELOID_LEUKEMIA	0.392837582	0.116171014	−0.144969141	−0.063128496	0.186717064	0.181921789	0.573873478	0.426843224	0.328029813	0.177535510	0.493901350	−0.114346324	0.303504038	0.057174591
36	CIRCADIAN_RHYTHM_MAMMAL	0.898813622	0.705534965	0.620348592	0.683449228	0.844728864	0.696904375	0.746885967	0.884497750	0.830821309	0.837880971	−0.253516497	0.403556679	0.751887562	0.766953748
37	CITRATE_CYCLE_TCA_CYCLE	0.512164414	0.763039862	0.625658769	0.445921663	0.371726044	−0.143710345	0.515077179	0.535318878	0.624197194	0.478275061	−0.351259463	0.943966449	0.702261165	0.524766442
38	COLORECTAL_CANCER	0.355538194	0.348854553	−0.140024320	−0.254570125	−0.076014528	−0.491977318	0.789303603	0.447876041	0.430748434	0.027595592	0.658577000	0.442002120	0.545205788	−0.053264561
39	COMPLEMENT_AND_COAGULATION_CASCADES	0.503346540	0.193677311	0.198994447	0.355451944	0.537288694	0.697262246	0.364579044	0.477133642	0.380566936	0.471156116	−0.032882591	−0.173534262	0.266748891	0.383493470
40	CYSTEINE_AND_METHIONINE_METABOLISM	−0.027750601	0.298212829	0.344506305	0.178707117	−0.014541236	−0.315574556	−0.076926924	−0.025250771	0.087603845	0.059825955	−0.398724369	0.580084295	0.165037029	0.158500620
41	CYTOKINE_CYTOKINE_RECEPTOR_INTERACTION	0.561656720	0.268351732	0.120627164	0.232553729	0.458630549	0.486613038	0.572958678	0.566495798	0.469926373	0.428134148	0.190173230	−0.043909492	0.399913225	0.320518179
42	CYTOSOLIC_DNA_SENSING_PATHWAY	0.540513775	0.253206486	0.353531485	0.518135230	0.657135020	0.841085299	0.287657028	0.494089699	0.409942174	0.579442567	−0.254072017	−0.131751044	0.273705313	0.512997674
43	DILATED_CARDIOMYOPATHY	0.687898475	0.419483613	0.268388097	0.362865917	0.580478605	0.538937926	0.667477079	0.690747977	0.604786818	0.560789682	0.085730056	0.108637134	0.535467169	0.459139100
44	DNA_REPLICATION	−0.231226451	0.073534503	−0.109066656	−0.309335738	−0.420638053	−0.775960018	0.025307248	−0.175665414	−0.083603730	−0.313347531	0.177647846	0.447325699	0.063077658	−0.256229246
45	DORSO_VENTRAL_AXIS_FORMATION	−0.076881562	−0.364976220	−0.119700610	0.093315836	0.165015981	0.643554385	−0.316769237	−0.136301296	−0.225450854	0.040774982	−0.101128881	−0.690750321	−0.367894883	−0.000587708
46	DRUG_METABOLISM_CYTOCHROME_P450	0.489147714	0.176427165	0.119063328	0.263536912	0.467605510	0.596191185	0.422564182	0.476387565	0.375808675	0.412425750	0.094127735	−0.170383621	0.279393974	0.314001458
47	DRUG_METABOLISM_OTHER_ENZYMES	0.452660037	0.135504247	0.084661722	0.233920654	0.437078910	0.586360523	0.389155402	0.439407250	0.336945001	0.378411706	0.111380153	−0.211070058	0.239537420	0.279916656
48	ECM_RECEPTOR_INTERACTION	0.859209940	0.657440048	0.471012394	0.517111259	0.715716141	0.526383513	0.828551443	0.866969205	0.805297948	0.725147015	−0.033486107	0.388125109	0.754430908	0.635902047
49	ENDOCYTOSIS	0.168339103	−0.144092707	0.023169432	0.225764868	0.344424092	0.718808066	−0.053969303	0.117507548	0.021038845	0.236272157	−0.099412870	−0.508739394	−0.119964076	0.174801135
50	ENDOMETRIAL_CANCER	−0.467858149	−0.576042419	−0.135870442	0.032955781	−0.070421633	0.471503827	−0.815298178	−0.550823727	−0.577047872	−0.197558569	−0.376623547	−0.736549788	−0.702984316	−0.156169247
51	EPITHELIAL_CELL_SIGNALING_IN_HELICOBACTER_PYLORI_INFECTION	0.601500448	0.316857030	0.372320149	0.523917445	0.678402774	0.811445615	0.380543354	0.562862615	0.478768807	0.611901496	−0.216118003	−0.061560018	0.352349814	0.538849294
52	ERBB_SIGNALING_PATHWAY	−0.584155679	−0.740226018	−0.382132705	−0.202716869	−0.256194425	0.322425964	−0.809192871	−0.648281600	−0.696494967	−0.383206207	−0.099556312	−0.896337035	−0.806159829	−0.367831854
53	ERBB_SIGNALING_PATHWAY.1	−0.487420735	−0.706673911	−0.416402027	−0.220301482	−0.213759708	0.356333644	−0.664150658	−0.542261216	−0.613270207	−0.341073274	0.023426139	−0.913955421	−0.726648963	−0.354512272
54	ETHER_LIPID_METABOLISM	0.306264533	−0.016251506	−0.121752033	0.021910648	0.241768129	0.410561495	0.342802967	0.308437944	0.199163960	0.187998704	0.315296854	−0.324086130	0.123652608	0.079856774
55	FATTY_ACID_METABOLISM	0.209123296	−0.056292789	0.223836454	0.425100110	0.481669372	0.864368689	−0.148195481	0.135093922	0.060304246	0.364965579	−0.380800371	−0.423095888	−0.101574803	0.334435876
56	FC_EPSILON_RI_SIGNALING_PATHWAY	0.762319886	0.514428699	0.363701076	0.445244548	0.653903356	0.566764818	0.720518384	0.763707316	0.686100411	0.641639398	0.013886911	0.208886146	0.618127497	0.545516935
57	FC_GAMMA_R_MEDIATED_PHAGOCYTOSIS	0.455954193	0.151306008	0.241634069	0.415551428	0.564692538	0.788236622	0.237693363	0.413411905	0.321399107	0.481965663	−0.161080371	−0.231159550	0.187926140	0.409390534
58	FOCAL_ADHESION	0.755956710	0.500682908	0.393945376	0.489479143	0.687197325	0.637365838	0.668198600	0.748030593	0.669694908	0.664305364	−0.054505129	0.175130126	0.587822762	0.573130374

	Pathways	Erismodegib	Vismodegib	Temsirolimus	Ibrutinib	RXDX-101	Ruxolitinib	Alectinib	Vemurafenib	Dabrafenib	Nilotinib	Dasatinib	Bosutinib	ABT-199	Dovitinib
1	ABC_TRANSPORTERS	0.069545636	0.043377927	0.530033049	0.078904058	0.364322606	0.418906421	−0.514636614	0.275726040	0.661757213	−0.220224365	−0.323709717	0.550382940	−0.173389418	0.058820563
2	ACUTE_MYELOID_LEUKEMIA	0.568638316	0.285218787	−0.085904776	0.628209684	0.483690884	0.188371953	0.110711900	0.261166414	0.208889812	0.421901208	0.218241684	0.373834616	0.303194384	0.420263387
3	ADHERENS_JUNCTION	0.623860591	0.342349306	0.054369722	0.682829349	0.590657446	0.310292862	0.059890048	0.358893992	0.366178555	0.427539687	0.206402821	0.511885375	0.319875770	0.477752938
4	ADIPOCYTOKINE_SIGNALING_PATHWAY	0.872631317	0.677439033	0.462574883	0.907167561	0.874546465	0.680063079	0.300320929	0.714254260	0.697752398	0.683574889	0.487895853	0.813971651	0.617361972	0.777546469
5	ALANINE_ASPARTATE_AND_GLUTAMATE_METABOLISM	−0.232303866	0.075072546	0.126393534	−0.305117759	−0.313847178	−0.046677092	0.452562067	−0.028054886	−0.243979815	0.058246441	0.295934846	−0.327185589	0.157122298	−0.067722848
6	ALDOSTERONE_REGULATED_SODIUM_REABSORPTION	0.413858391	0.163290045	−0.340499361	0.466400654	0.230327717	−0.058799075	0.228950206	0.056844324	−0.119275022	0.388792967	0.245754711	0.067079046	0.262443875	0.280488249
7	ALLOGRAFT_REJECTION	0.964707885	0.868670302	0.500720575	0.972055438	0.854991942	0.720939255	0.712608208	0.808435069	0.591015361	0.935443943	0.826461765	0.726127052	0.886270729	0.924216850
8	ALPHA_LINOLENIC_ACID_METABOLISM	0.430671785	0.646539417	0.402561541	0.368922686	0.256937570	0.403718225	0.933227285	0.492068399	0.116418693	0.699736316	0.850843139	0.141777938	0.751963665	0.553291581
9	ALZHEIMERS_DISEASE	0.962268511	0.844718079	0.669278352	0.976520303	0.970257301	0.848187352	0.469469227	0.878170099	0.823581183	0.812161372	0.657219875	0.916200637	0.777247966	0.911018668
10	AMINO_SUGAR_AND_NUCLEOTIDE_SUGAR_METABOLISM	0.863433712	0.692749131	0.585652016	0.892848249	0.919530175	0.762669801	0.235224374	0.768459734	0.812394664	0.641747708	0.447562924	0.896937622	0.595057189	0.782148099
11	AMINOACYL_TRNA_BIOSYNTHESIS	0.032190772	0.315455127	0.682854980	−0.035879394	0.161072694	0.458812545	0.224859441	0.381784322	0.417542388	0.097569968	0.240588775	0.269220684	0.229622664	0.189065590
12	AMYOTROPHIC_LATERAL_SCLEROSIS_ALS	0.296155059	−0.017904171	−0.143370307	0.369144640	0.341735014	0.057600900	−0.355000899	0.059050347	0.223463489	0.029541719	−0.207667371	0.327824305	−0.078180256	0.128455107
13	ANTIGEN_PROCESSING_AND_PRESENTATION	0.779717301	0.544442640	0.187956831	0.824431386	0.704777916	0.453634021	0.311600345	0.525270032	0.438423666	0.640764066	0.448108976	0.595185418	0.543957842	0.660699319
14	APOPTOSIS	0.682944850	0.434824719	0.009822450	0.731687364	0.564862528	0.291580111	0.301910469	0.382455297	0.257237433	0.581482695	0.400295151	0.430884674	0.470783754	0.555176237
15	ARACHIDONIC_ACID_METABOLISM	0.618146312	0.366884950	−0.088964091	0.668345516	0.478210715	0.197088018	0.291259798	0.297081028	0.152844213	0.538954727	0.366999815	0.332983860	0.422360425	0.487574919
16	ARGININE_AND_PROLINE_METABOLISM	0.919861563	0.794758346	0.376209450	0.934680518	0.786275720	0.619430397	0.679806198	0.718904247	0.489084053	0.896421953	0.779044988	0.641476956	0.831322705	0.862611150
17	ARRHYTHMOGENIC_RIGHT_VENTRICULAR_CARDIOMYOPATHY_ARVC	0.863276254	0.658468750	0.340979058	0.899530580	0.808035793	0.589252484	0.380587961	0.652048156	0.566810215	0.720541032	0.535203423	0.709836418	0.639721169	0.762275585
18	ASCORBATE_AND_ALDARATE_METABOLISM	0.604503311	0.368046669	−0.123248026	0.650931790	0.438519656	0.164300984	0.350590832	0.276112851	0.093053706	0.559580053	0.405360742	0.278369483	0.443513337	0.481625062
19	ASTHMA	0.948352463	0.801779164	0.480313011	0.968876009	0.879630910	0.709988217	0.551724546	0.779114022	0.639702219	0.849996557	0.698739901	0.773675140	0.789365852	0.880309371
20	AUTOIMMUNE_THYROID_DISEASE	0.788810239	0.558044453	0.193814286	0.832212335	0.708717624	0.460224408	0.333862954	0.534466281	0.437782287	0.656855838	0.467768746	0.595722173	0.560850900	0.672340958
21	AXON_GUIDANCE	0.291148987	−0.014612832	−0.080982123	0.362609286	0.368006480	0.100149158	−0.401276647	0.085499585	0.287400519	0.001863210	−0.238247905	0.375472907	−0.096801045	0.128212356
22	B_CELL_RECEPTOR_SIGNALING_PATHWAY	0.708975148	0.447458455	0.147554116	0.761668040	0.669319320	0.404461564	0.160377120	0.457779647	0.437243846	0.525704540	0.312263700	0.583876754	0.425093488	0.574928627
23	BASAL_CELL_CARCINOMA	0.103322477	0.414950103	0.555059466	0.025396523	0.097563387	0.384022008	0.555757028	0.374599004	0.214028995	0.304681081	0.497162202	0.117034857	0.422416272	0.275493637
24	BASAL_TRANSCRIPTION_FACTORS	−0.194040101	0.123754673	0.235149847	−0.269516226	−0.241069476	0.044553009	0.433570598	0.046630570	−0.133863007	0.069303332	0.301065725	−0.232912113	0.179018941	−0.022914267
25	BASE_EXCISION_REPAIR	−0.266189658	0.049777799	0.175085266	−0.340079782	−0.310145166	−0.024263370	0.375658607	−0.025736448	−0.192572690	−0.001869315	0.233696322	−0.296513881	0.107135587	−0.097020705
26	BETA_ALANINE_METABOLISM	−0.994574634	−0.952290847	−0.770854997	−0.989587559	−0.978693335	−0.919596870	−0.648620532	−0.958690949	−0.835230323	−0.913026683	−0.810170156	−0.914330663	−0.902328665	−0.984805351
27	BIOSYNTHESIS_OF_UNSATURATED_FATTY_ACIDS	0.047687836	0.336122373	0.681530768	−0.022061732	0.163465375	0.462213265	0.266637129	0.391897622	0.406410745	0.127283428	0.275965478	0.262770044	0.258926637	0.207489064
28	BLADDER_CANCER	0.448324194	0.272594588	−0.302220926	0.481191730	0.203603948	−0.033107289	0.475110685	0.110279282	−0.183242314	0.525309828	0.444182414	0.005599261	0.415922502	0.355891352
29	BUTANOATE_METABOLISM	−0.547172418	−0.336261461	0.202650978	−0.587679553	−0.341487543	−0.081131235	−0.420400457	−0.209740491	0.029355261	−0.558936430	−0.436600782	−0.160706740	−0.444496142	−0.437155867
30	CALCIUM_SIGNALING_PATHWAY	−0.072146813	−0.347808698	−0.225679554	−0.001550544	0.076755022	−0.137920487	−0.733354032	−0.200492787	0.125917410	−0.382295951	−0.593702954	0.152205662	−0.458884954	−0.222881963
31	CARDIAC_MUSCLE_CONTRACTION	−0.538444861	−0.275136419	0.182784438	−0.592754954	−0.394865114	−0.102798040	−0.224502087	−0.202860202	−0.069088382	−0.460190663	−0.287963710	−0.250185921	−0.337673837	−0.400150958
32	CELL_ADHESION_MOLECULES_CAMS	0.556752109	0.263426633	−0.029021739	0.619576601	0.519412550	0.227990356	0.003030828	0.277262605	0.292724646	0.361101748	0.138961828	0.440253685	0.247615207	0.403216733
33	CELL_CYCLE	−0.564077463	−0.276833529	−0.097524046	−0.626174902	−0.590893237	−0.322417700	0.096424874	−0.339720813	−0.434849091	−0.313578968	−0.077075231	−0.553697036	−0.214678210	−0.414556724
34	CHEMOKINE_SIGNALING_PATHWAY	0.573567033	0.317933156	−0.146158839	0.625420974	0.426776440	0.140423994	0.266062615	0.242733332	0.097029379	0.500820495	0.331551859	0.278935165	0.380891481	0.439929345
35	CHRONIC_MYELOID_LEUKEMIA	0.462262655	0.254078551	−0.297451065	0.503194142	0.241944256	−0.018526213	0.392033263	0.114735473	−0.134184051	0.494945414	0.385228060	0.056165621	0.378038040	0.352451235
36	CIRCADIAN_RHYTHM_MAMMAL	0.955900399	0.822457172	0.483313527	0.972909806	0.873541218	0.712283671	0.601091863	0.787763222	0.623801331	0.877983404	0.738266598	0.759522072	0.819788228	0.895033704
37	CITRATE_CYCLE_TCA_CYCLE	0.351180835	0.631029086	0.718268805	0.277238997	0.343493194	0.596032600	0.681464754	0.596387376	0.417256826	0.516519444	0.669228900	0.346308124	0.623934084	0.509292292
38	COLORECTAL_CANCER	0.246164360	0.326121499	−0.164011459	0.218919488	−0.042780946	−0.050706229	0.803568730	0.096177878	−0.345213029	0.543998775	0.654810139	−0.236059345	0.521282289	0.290728245
39	COMPLEMENT_AND_COAGULATION_CASCADES	0.644034613	0.370228053	0.027032801	0.700365407	0.578097351	0.295697101	0.143553255	0.360779108	0.319614528	0.481102708	0.272276142	0.477694652	0.370288283	0.502128525
40	CYSTEINE_AND_METHIONINE_METABOLISM	−0.174615870	0.127084039	0.500814619	−0.244296393	−0.063523080	0.249744432	0.142067458	0.174595713	0.204293163	−0.066821204	0.109556305	0.045153510	0.067088321	−0.010614912
41	CYTOKINE_CYTOKINE_RECEPTOR_INTERACTION	0.660245823	0.426777870	−0.047446417	0.705275400	0.507499242	0.238782104	0.370051534	0.345500663	0.171451118	0.601406603	0.440959286	0.353430132	0.489460145	0.539885274
42	CYTOSOLIC_DNA_SENSING_PATHWAY	0.690174438	0.425294365	0.190926061	0.744486488	0.688007978	0.430489728	0.075836671	0.464790519	0.496753814	0.471929602	0.247534136	0.627619444	0.377029903	0.554367814
43	DILATED_CARDIOMYOPATHY	0.771231822	0.567194785	0.105807264	0.807465307	0.625469601	0.384272764	0.478891968	0.489324098	0.297478893	0.717604592	0.565100896	0.473036156	0.619059799	0.668579100
44	DNA_REPLICATION	−0.413222537	−0.106773639	0.045879476	−0.482446163	−0.449734485	−0.168389871	0.246727696	−0.177400194	−0.312261650	−0.151762327	0.087524639	−0.424708672	−0.046174663	−0.251207105
45	DORSO_VENTRAL_AXIS_FORMATION	0.116718193	−0.195977985	−0.250984234	0.192463961	0.188889532	−0.083967917	−0.523630869	−0.101055580	0.119263247	−0.160833354	−0.390022690	0.201469391	−0.264477013	−0.052438682
46	DRUG_METABOLISM_CYTOCHROME_P450	0.618060147	0.349912714	−0.054034434	0.672872057	0.513271896	0.225993622	0.203172301	0.309345713	0.218367034	0.495916859	0.303497828	0.389396264	0.379625539	0.478550110
47	DRUG_METABOLISM_OTHER_ENZYMES	0.585847079	0.311003678	−0.088679814	0.642815357	0.483091814	0.190643517	0.167193169	0.272370929	0.190997433	0.459704795	0.265042752	0.361553817	0.340996092	0.442246202
48	ECM_RECEPTOR_INTERACTION	0.899328625	0.770942374	0.331283193	0.915089364	0.752956496	0.579910089	0.683342205	0.685298696	0.442871656	0.886781592	0.772481291	0.600567509	0.817831581	0.839883018
49	ENDOCYTOSIS	0.351977756	0.037844146	−0.132377586	0.423843140	0.375970178	0.085039882	−0.278398312	0.098856498	0.228430598	0.101097224	−0.134717819	0.345179611	−0.010297782	0.184802074
50	ENDOMETRIAL_CANCER	−0.311296531	−0.497963286	−0.161083566	−0.257774950	−0.083553439	−0.190398190	−0.902342592	−0.303529551	0.114460475	−0.618616429	−0.775548402	0.063419591	−0.648500254	−0.415794211
51	EPITHELIAL_CELL_SIGNALING_IN_HELICOBACTER_PYLORI_INFECTION	0.738978332	0.485738243	0.207654354	0.789358003	0.711727468	0.457460930	0.171640025	0.505085741	0.493250733	0.547766968	0.333783263	0.633604778	0.452903705	0.609989765
52	ERBB_SIGNALING_PATHWAY	−0.419877441	−0.644923144	−0.424783713	−0.356184342	−0.256315155	−0.414554253	−0.921730260	−0.497082015	−0.131827394	−0.686424755	−0.841469401	−0.149161210	−0.743973863	−0.547473183
53	ERBB_SIGNALING_PATHWAY.1	−0.309194100	−0.580293441	−0.494277429	−0.236660941	−0.200732641	−0.418449691	−0.817923701	−0.468088864	−0.167193671	−0.563307637	−0.741551101	−0.139244184	−0.647860128	−0.461024750
54	ETHER_LIPID_METABOLISM	0.433149634	0.155054592	−0.290683967	0.492727098	0.293042131	−0.010185878	0.123332386	0.085793775	−0.023901298	0.349338504	0.175192765	0.154585627	0.221003389	0.285347320
55	FATTY_ACID_METABOLISM	0.393612534	0.109367199	0.089273686	0.458630527	0.498548914	0.256712818	−0.347756901	0.230051448	0.446158567	0.085790755	−0.156556382	0.521095258	0.001479463	0.244120518
56	FC_EPSILON_RI_SIGNALING_PATHWAY	0.834342861	0.652869185	0.207148619	0.864189832	0.695620825	0.476322183	0.544213640	0.578614062	0.378007888	0.784690977	0.639585353	0.546647449	0.696557604	0.744981750
57	FC_GAMMA_R_MEDIATED_PHAGOCYTOSIS	0.613511011	0.330071604	0.074954637	0.673364872	0.599109434	0.322215783	0.016490202	0.361264309	0.395099668	0.399555866	0.172845020	0.532926813	0.294657221	0.466375809
58	FOCAL_ADHESION	0.841167619	0.645990651	0.234486176	0.874598038	0.726925170	0.501426759	0.484645688	0.592425530	0.428944351	0.759860246	0.598704735	0.592298123	0.671260290	0.744609983

	Pathways	cluster	Afatinib	Erlotinib HCl	Dacomitinib	Gefitinib	Lapatinib	Neratinib	CO-1686	Everolimus	BYL719	Pilaralisib	XL765	Pazopanib HCl	Sunitinib Malate
59	FOLATE_BIOSYNTHESIS	C1	-0.320957654	-0.266162661	-0.715245038	-0.492202618	-0.803719165	-0.452819498	-0.424967191	-0.761695322	-0.710787866	-0.621386640	-0.766225186	-0.411338962	-0.098547453
60	FRUCTOSE_AND_MANNOSE_METABOLISM	C3	0.159508783	0.390986866	-0.500247369	0.211766579	-0.265831561	-0.097668377	-0.267331430	-0.200658284	-0.091563762	-0.040097923	-0.169179855	0.225661970	0.446594888
61	GALACTOSE_METABOLISM	C2	0.969147315	0.948460391	0.590765022	0.757041274	0.698477023	0.862301726	0.700134798	0.742560687	0.755722518	0.861733213	0.704704364	0.941271291	0.998055484
62	GAP_JUNCTION	C3	-0.153726350	-0.454930046	-0.136950296	-0.766307255	-0.634486921	-0.069564569	0.148345885	-0.624423645	-0.696353615	-0.515487037	-0.718230863	-0.475564262	-0.165729920
63	GLIOMA	C1	-0.578223731	-0.347074833	-0.950145770	-0.397266824	-0.822580561	-0.749898518	-0.785987361	-0.790299046	-0.695086545	-0.714497287	-0.735143750	-0.507478662	-0.314449300
64	GLUTATHIONE_METABOLISM	C2	0.797555077	0.543046273	0.998771362	0.475212094	0.845998155	0.924643534	0.936511438	0.834025674	0.744438744	0.824335738	0.759047258	0.676847433	0.576006906
65	GLYCEROLIPID_METABOLISM	C2	0.923395028	0.775630129	0.588424695	0.478042633	0.499664898	0.862877030	0.788826146	0.543912908	0.523402466	0.694615223	0.468590412	0.772167186	0.928641898
66	GLYCEROPHOSPHOLIPID_METABOLISM	C2	0.996513101	0.928433480	0.744640875	0.772865651	0.816634864	0.939791914	0.801386325	0.848379009	0.839078998	0.935944803	0.803067732	0.958951238	0.962026114
67	GLYCINE_SERINE_AND_THREONINE_METABOLISM	C2	0.707255446	0.528377219	0.331302158	0.154942926	0.132791691	0.642651734	0.628325212	0.182762819	0.161238514	0.367570257	0.096679837	0.489459003	0.757931426
68	GLYCOLYSIS_GLUCONEOGENESIS	C2	0.750221845	0.741207857	0.208116634	0.437748413	0.241490364	0.591507895	0.463650617	0.303412062	0.336246570	0.487736464	0.260820604	0.664382566	0.882472850
69	GLYCOSAMINOGLYCAN_BIOSYNTHESIS_CHONDROITIN_SULFATE	C2	0.976283301	0.810297128	0.738289190	0.561360129	0.657732501	0.949436257	0.876318734	0.691817664	0.658008858	0.810609273	0.616302872	0.839653441	0.930051339
70	GLYCOSAMINOGLYCAN_BIOSYNTHESIS_HEPARAN_SULFATE	C3	0.091871084	0.164024524	-0.470427803	-0.121059914	-0.474298964	-0.094420189	-0.152076477	-0.413774253	-0.350370223	-0.229683577	-0.426507902	0.014631894	0.331858083
71	GLYCOSAMINOGLYCAN_BIOSYNTHESIS_KERATAN_SULFATE	C2	0.727427579	0.463453789	0.486275021	0.091883539	0.194162529	0.725329563	0.759771698	0.232364071	0.178683633	0.396450897	0.127373807	0.462703028	0.708810395
72	GLYCOSAMINOGLYCAN_DEGRADATION	C3	0.517513616	0.153575118	0.462508121	-0.216149622	0.011303962	0.597036444	0.731531386	0.032397559	-0.058100705	0.169324152	-0.091218469	0.181051182	0.437901461
73	GLYCOSPHINGOLIPID_BIOSYNTHESIS_GANGLIO_SERIES	C3	0.561085512	0.246547029	0.397559711	-0.139624424	0.006540531	0.595333533	0.691596026	0.037912187	-0.032153043	0.196530484	-0.077051398	0.249002155	0.527257523
74	GLYCOSPHINGOLIPID_BIOSYNTHESIS_GLOBO_SERIES	C3	0.487021617	0.052370244	0.804253123	-0.149864556	0.327455713	0.695913357	0.877452062	0.309420265	0.170983606	0.331873472	0.186673569	0.181227617	0.242321143
75	GLYCOSPHINGOLIPID_BIOSYNTHESIS_LACTO_AND_NEOLACTO_SERIES	C1	-0.813404675	-0.848064486	-0.756442526	-0.910909594	-0.990724122	-0.787607486	-0.616544386	-0.996279226	-0.997963336	-0.978295342	-0.997329896	-0.918381894	-0.727901885
76	GLYCOSYLPHOSPHATIDYLINOSITOL_GPI_ANCHOR_BIOSYNTHESIS	C1	-0.593801464	-0.846520567	-0.336549442	-0.985954731	-0.805914957	-0.450970876	-0.184625131	-0.825169146	-0.899416132	-0.810724522	-0.885042016	-0.842994392	-0.645650014
77	GLYOXYLATE_AND_DICARBOXYLATE_METABOLISM	C3	-0.025285995	0.394667499	-0.582134053	0.462023686	-0.075971855	-0.301469882	-0.556723225	-0.030053763	0.118475653	0.028578111	0.069416812	0.245215303	0.258787782
78	GNRH_SIGNALING_PATHWAY	C2	0.720389334	0.354570949	0.943169540	0.180076802	0.605408910	0.883145474	0.979159852	0.595411126	0.478237505	0.620817973	0.486884774	0.483011655	0.490508395
79	GRAFT_VERSUS_HOST_DISEASE	C2	0.806595706	0.487997709	0.721449681	0.168160001	0.402055974	0.865319724	0.918297980	0.424917461	0.343819010	0.547127103	0.311591414	0.538642288	0.703796244
80	HEDGEHOG_SIGNALING_PATHWAY	C1	-0.591859422	-0.294177820	-0.396295044	0.093093431	-0.030838212	-0.612149195	-0.692396331	-0.065164882	-0.001427952	-0.228762681	0.046661580	-0.291200481	-0.569246494
81	HEMATOPOIETIC_CELL_LINEAGE	C3	0.545398872	0.138211944	0.622638033	-0.187613653	0.143635231	0.675662941	0.831164708	0.151577714	0.039128124	0.253186160	0.022181603	0.205221891	0.400163659
82	HERPES_SIMPLEX_VIRUS_1_INFECTION	C2	0.856253692	0.544098094	0.816402222	0.264353981	0.529889481	0.926779855	0.966180701	0.547421970	0.463368834	0.648429179	0.438577428	0.613157633	0.727223869
83	HISTIDINE_METABOLISM	C3	-0.007395117	-0.447108753	0.355342094	-0.663966064	-0.236504494	0.216206099	0.491548725	-0.259783023	-0.396296082	-0.236387743	-0.377385602	-0.357379006	-0.203508790
84	HOMOLOGOUS_RECOMBINATION	C3	-0.221322958	0.099438810	-0.121443574	0.477374927	0.344972587	-0.276036196	-0.438218541	0.320741748	0.392870011	0.172766298	0.431029465	0.114670725	-0.206450417
85	HUMAN_PAPILLOMAVIRUS_INFECTION	C2	0.863384497	0.572009756	0.754423592	0.269165651	0.484823771	0.907879891	0.933455158	0.509315694	0.435988735	0.628727182	0.402738879	0.622637031	0.765462851
86	HUNTINGTONS_DISEASE	C2	0.670862706	0.736348511	0.706952094	0.880343513	0.977394502	0.664603257	0.499574937	0.971140397	0.974448256	0.911259598	0.988949905	0.819305349	0.567116572
87	HYPERTROPHIC_CARDIOMYOPATHY_HCM	C2	0.764141701	0.422106539	0.718585262	0.100592945	0.362521038	0.840798011	0.916023646	0.381832536	0.292347517	0.498989769	0.263833489	0.478917846	0.647875721
88	INOSITOL_PHOSPHATE_METABOLISM	C1	-0.523552458	-0.391002440	-0.872793749	-0.523616155	-0.878771690	-0.662526308	-0.646749933	-0.845064950	-0.776324819	-0.743606924	-0.820380373	-0.542182924	-0.285892512
89	INSULIN_SIGNALING_PATHWAY	C2	0.993459185	0.873926238	0.730116110	0.657653376	0.719174227	0.946856032	0.841524126	0.754267663	0.733558576	0.865118628	0.691854637	0.899248917	0.959970270
90	INTESTINAL_IMMUNE_NETWORK_FOR_IGA_PRODUCTION	C2	0.732944732	0.410746135	0.614036744	0.059364444	0.263219045	0.782543370	0.851627957	0.289981009	0.211891522	0.428768092	0.173988563	0.443846423	0.654912467
91	JAK_STAT_SIGNALING_PATHWAY	C3	0.518397761	0.116652327	0.570687906	-0.221972200	0.087531817	0.639796621	0.797048649	0.098050098	-0.010607456	0.208035898	-0.030949805	0.173832667	0.388340517
92	LEISHMANIA_INFECTION	C2	0.737847646	0.444046361	0.556822113	0.081790675	0.235844399	0.761492392	0.812304523	0.268471836	0.202389965	0.420294734	0.157629594	0.460719815	0.688828474
93	LEUKOCYTE_TRANSENDOTHELIAL_MIGRATION	C3	0.596334555	0.284781410	0.427960999	-0.098690574	0.048803050	0.628366416	0.716301274	0.080624150	0.011189956	0.238823866	-0.033968949	0.289646973	0.559973054
94	LIMONENE_AND_PINENE_DEGRADATION	C1	-0.159369619	-0.374510956	0.496039148	-0.181291609	0.285807620	0.092217003	0.251598712	0.220470537	0.115682062	0.055578515	0.193931048	-0.209915534	-0.443153589
95	LINOLEIC_ACID_METABOLISM	C2	0.525045717	0.663404343	-0.126663171	0.422435664	0.053502034	0.294876929	0.116535800	0.121730341	0.202521695	0.301205964	0.121106710	0.535984368	0.749538098
96	LONG_TERM_DEPRESSION	C1	-0.724880626	-0.624554358	-0.907369322	-0.697533766	-0.970574714	-0.805819439	-0.734084782	-0.954495872	-0.904716278	-0.897905211	-0.928106313	-0.750082443	-0.535040787
97	LONG_TERM_POTENTIATION	C1	-0.833348920	-0.676441109	-0.960298332	-0.663515286	-0.948593491	-0.912676613	-0.857090160	-0.941106086	-0.880813217	-0.922719674	-0.892349317	-0.795711111	-0.647826730
98	LYSINE_DEGRADATION	C3	-0.137575052	0.172495885	-0.043190240	0.542460311	0.425431897	-0.191083778	-0.361898421	0.402301877	0.471131300	0.257552054	0.508172634	0.194634975	-0.132903555
99	LYSOSOME	C2	0.689840297	0.384781404	0.519995269	0.014428649	0.172881628	0.719757338	0.786403719	0.204668914	0.135607081	0.357954960	0.091313319	0.399263731	0.641641196
100	MAPK_SIGNALING_PATHWAY	C3	0.577908229	0.181626533	0.623424447	-0.151084585	0.161291470	0.695873922	0.838787819	0.172392676	0.064784107	0.280981142	0.044241222	0.243293974	0.442991938
101	MAPK_SIGNALING_PATHWAY.1	C3	0.538064113	0.181930245	0.464523815	-0.190218847	0.025760211	0.610024971	0.735975969	0.048643021	-0.038702901	0.189071937	-0.073801067	0.206492690	0.464388041
102	MATURITY_ONSET_DIABETES_OF_THE_YOUNG	C1	-0.774922200	-0.732494545	-0.856764283	-0.803424110	-0.996740079	-0.810696399	-0.694118522	-0.989716292	-0.961667244	-0.948503220	-0.975128896	-0.836202377	-0.627247780
103	MELANOGENESIS	C3	0.326114626	-0.124578827	0.707506798	-0.314216092	0.183094926	0.558266720	0.778989024	0.158368884	0.012934294	0.167654786	0.035778465	0.005256480	0.074340473
104	MELANOMA	C3	-0.049080757	-0.449393926	0.159379449	-0.729569860	-0.419286431	0.118229677	0.377714116	-0.426786442	-0.540229320	-0.357841141	-0.541098857	-0.407087996	-0.168318774
105	METABOLISM_OF_XENOBIOTICS_BY_CYTOCHROME_P450	C3	0.413166000	0.006199701	0.479809385	-0.336760133	-0.033089114	0.540174571	0.719963102	-0.024046884	-0.133687458	0.085882983	-0.152982802	0.056263413	0.291044310
106	MISMATCH_REPAIR	C1	-0.283511370	0.026872713	-0.147250063	0.412929469	0.297143469	-0.324688328	-0.469191840	0.269188756	0.336630027	0.113253683	0.378925832	0.045676760	-0.276814220
107	MTOR_SIGNALING_PATHWAY	C3	0.376982464	-0.071853334	0.726660596	-0.276235897	0.209552166	0.599224577	0.809680085	0.188357709	0.045321129	0.207323097	0.063980941	0.054295285	0.132178211
108	N_GLYCAN_BIOSYNTHESIS	C2	0.688191438	0.502167996	0.961140056	0.542984900	0.905384881	0.818579045	0.806828526	0.882519636	0.804969476	0.824149841	0.834720855	0.647143945	0.456381592
109	NATURAL_KILLER_CELL_MEDIATED_CYTOTOXICITY	C3	0.508473238	0.127250954	0.498083865	-0.231894471	0.028929739	0.605817019	0.752490955	0.045682333	-0.052611459	0.172289647	-0.080596214	0.166040736	0.409061965
110	NEUROACTIVE_LIGAND_RECEPTOR_INTERACTION	C1	-0.778490132	-0.792950926	-0.787281478	-0.875110749	-0.998078991	-0.777562663	-0.625100192	-0.997011576	-0.988783940	-0.962604883	-0.995991659	-0.877832463	-0.667277305
111	NEUROTROPHIN_SIGNALING_PATHWAY	C3	0.370055407	-0.008317212	0.362257711	-0.373779650	-0.134997223	0.467374849	0.637707143	-0.118739861	-0.214611995	0.010423652	-0.242849276	0.016738921	0.289065962
112	NICOTINATE_AND_NICOTINAMIDE_METABOLISM	C2	0.844604961	0.569187261	0.987009092	0.445192827	0.798878317	0.959705437	0.976579068	0.794399018	0.704048735	0.813136404	0.708715313	0.690426923	0.640468785
113	NITROGEN_METABOLISM	C1	-0.450765716	-0.043341027	-0.869712092	0.067399876	-0.451074959	-0.682755569	-0.852962465	-0.420466752	-0.280557882	-0.395530938	-0.313026658	-0.198395610	-0.168882952
114	NOD_LIKE_RECEPTOR_SIGNALING_PATHWAY	C3	0.577886842	0.228965691	0.489319065	-0.142406934	0.067551308	0.643491643	0.757695236	0.091864276	0.006961129	0.234042066	-0.029531230	0.253630161	0.505868943
115	NON_HOMOLOGOUS_END_JOINING	C3	-0.186741581	0.109871546	-0.051225288	0.488911122	0.394896633	-0.224486067	-0.378117811	0.367639260	0.430455339	0.213822071	0.472169354	0.138220450	-0.194411624
116	NON_SMALL_CELL_LUNG_CANCER	C1	-0.784895509	-0.758534695	-0.264903405	-0.454371992	-0.285201861	-0.637336589	-0.513841041	-0.345348604	-0.371648355	-0.526474987	-0.298326078	-0.691166511	-0.901110165

	Pathways	Axitinib	Tivozanib	Vandetanib	Regorafenib	Selumetinib	Trametinib	Crizotinib	Cabozantinib	Foretinib	Palbociclib HCl	Ribociclib	Abemaciclib	Veliparib	Olaparib
59	FOLATE_BIOSYNTHESIS	-0.358180537	-0.507522664	-0.080214431	0.105293891	0.031959489	0.572584546	-0.721124026	-0.442991101	-0.481874754	-0.100071650	-0.385102904	-0.719822849	-0.621541828	-0.072647109
60	FRUCTOSE_AND_MANNOSE_METABOLISM	0.107563743	0.094829478	0.556651808	0.663977661	0.519376750	0.786305990	-0.424390259	0.007127669	0.024712230	0.429672288	-0.901801340	-0.087572287	-0.113267308	0.499096652
61	GALACTOSE_METABOLISM	0.954671852	0.920420682	0.944428027	0.944227374	0.984338336	0.721034680	0.665337381	0.921431777	0.927805351	0.999018785	-0.652809154	0.715308903	0.858684511	0.989738794
62	GAP_JUNCTION	-0.152785372	-0.465631456	-0.360061902	-0.162898056	-0.033277775	0.408532673	-0.219195852	-0.181599026	-0.286612296	-0.139048125	0.230151672	-0.754313644	-0.392693499	-0.209257335
63	GLIOMA	-0.619054002	-0.621685131	-0.174155526	-0.057126384	-0.224927662	0.261508762	-0.934803047	-0.695585902	-0.688133620	-0.330700315	-0.395883105	-0.663601182	-0.778959821	-0.258048301
64	GLUTATHIONE_METABOLISM	0.828414215	0.770764189	0.406222718	0.335135693	0.514306561	0.073676725	0.997196023	0.879489691	0.858778395	0.594594929	0.164885365	0.694761405	0.898444216	0.517491050
65	GLYCEROLIPID_METABOLISM	0.912541617	0.763565555	0.794846150	0.859575121	0.958191749	0.832656556	0.629219774	0.874942242	0.841691367	0.940594386	-0.520445402	0.460529753	0.741645961	0.900520742
66	GLYCEROPHOSPHOLIPID_METABOLISM	0.992960079	0.966414659	0.884272791	0.855217301	0.927923711	0.577572082	0.808381554	0.981190684	0.986279040	0.966316632	-0.492920983	0.796524198	0.946668521	0.942185161
67	GLYCINE_SERINE_AND_THREONINE_METABOLISM	0.689820805	0.462965144	0.605127726	0.742503111	0.839299923	0.937439120	0.348970100	0.632511819	0.573490587	0.773507911	-0.492855085	0.092386431	0.433767114	0.730078641
68	GLYCOLYSIS_GLUCONEOGENESIS	0.718976244	0.599589264	0.830715043	0.928917784	0.938477390	0.970899099	0.271136107	0.645936684	0.628000683	0.884037077	-0.784564202	0.290969362	0.491164383	0.884710536
69	GLYCOSAMINOGLYCAN_BIOSYNTHESIS_CHONDROITIN_SULFATE	0.974194904	0.853558467	0.789095963	0.815509002	0.932759999	0.697538269	0.777438110	0.955944663	0.929324603	0.942873979	-0.416377754	0.596741686	0.860213012	0.895290597
70	GLYCOSAMINOGLYCAN_BIOSYNTHESIS_HEPARAN_SULFATE	0.047752324	-0.093047848	0.344152790	0.516970090	0.448259833	0.852689397	-0.438333903	-0.050315443	-0.078610162	0.328498311	-0.685711779	-0.371739942	-0.241287386	0.358392629
71	GLYCOSAMINOGLYCAN_BIOSYNTHESIS_KERATAN_SULFATE	0.721725444	0.467606648	0.507707128	0.636272980	0.783071166	0.835201799	0.483643432	0.683983163	0.611795199	0.730980974	-0.300388856	0.100658730	0.491276283	0.663587546
72	GLYCOSAMINOGLYCAN_DEGRADATION	0.524030497	0.215816855	0.185927479	0.333209614	0.526701623	0.656322595	0.417822053	0.504674370	0.407448056	0.468385397	0.015884924	-0.142091881	0.302673852	0.375459732
73	GLYCOSPHINGOLIPID_BIOSYNTHESIS_GANGLIO_SERIES	0.559261028	0.262610377	0.300522398	0.455782383	0.621358882	0.769800260	0.369314618	0.525081003	0.435371463	0.553915151	-0.143347550	-0.113422247	0.312916887	0.474869319
74	GLYCOSPHINGOLIPID_BIOSYNTHESIS_GLOBO_SERIES	0.523396459	0.290018351	-0.033431659	0.007707031	0.259000730	0.127230806	0.735276912	0.568308420	0.483910447	0.276938636	0.475239307	0.096087982	0.481251586	0.158310402
75	GLYCOSPHINGOLIPID_BIOSYNTHESIS_LACTO_AND_NEOLACTO_SERIES	-0.821801575	-0.948838572	-0.732636428	-0.594649663	-0.623668706	-0.085909542	-0.827343667	-0.848401998	-0.897087556	-0.722672756	0.289871322	-0.990702407	-0.946135461	-0.720846500
76	GLYCOSYLPHOSPHATIDYLINOSITOL_GPI_ANCHOR_BIOSYNTHESIS	-0.581050796	-0.812210839	-0.785829211	-0.639835069	-0.541254456	-0.093150006	-0.444543441	-0.578264164	-0.666447765	-0.623212833	0.580023344	-0.926858111	-0.704418379	-0.681571607
77	GLYOXYLATE_AND_DICARBOXYLATE_METABOLISM	-0.073284455	0.115225640	0.502270159	0.488291945	0.253965599	0.324191088	-0.477887453	-0.145849732	-0.065991076	0.225392481	-0.849024829	0.169465831	-0.118291441	0.339915217
78	GNRH_SIGNALING_PATHWAY	0.752498265	0.582496187	0.250191528	0.249176328	0.476148738	0.197554548	0.909423779	0.795012717	0.735966624	0.518931808	0.272089321	0.408360572	0.740286230	0.415789202
79	GRAFT_VERSUS_HOST_DISEASE	0.815073640	0.580686060	0.474634337	0.557495121	0.745481982	0.654907640	0.709812853	0.806064255	0.735947389	0.729595139	-0.116974118	0.263326465	0.656283674	0.644156508
80	HEDGEHOG_SIGNALING_PATHWAY	-0.587847336	-0.299401723	-0.351134318	-0.504306518	-0.661682731	-0.800836595	-0.374798769	-0.550245389	-0.464447547	-0.594520356	0.196219645	0.078814132	-0.338790853	-0.519861578
81	HEMATOPOIETIC_CELL_LINEAGE	0.563277905	0.269236582	0.127121848	0.240659714	0.465305390	0.497032006	0.568042729	0.566867671	0.470318780	0.433578433	0.179600912	-0.045816733	0.398453663	0.326641850
82	HERPES_SIMPLEX_VIRUS_1_INFECTION	0.869126877	0.666302134	0.504102598	0.555933738	0.747294844	0.570962692	0.809387286	0.871482629	0.810678027	0.752045938	-0.083284418	0.386325874	0.752229196	0.666319159
83	HISTIDINE_METABOLISM	0.024637660	-0.265780329	-0.470964278	-0.359854871	-0.132715999	0.032909595	0.246844650	0.056827482	-0.050827252	-0.167172793	0.654286432	-0.462862219	-0.074186814	-0.281136707
84	HOMOLOGOUS_RECOMBINATION	-0.219130895	0.106465600	0.014458829	-0.177187495	-0.329795490	-0.654069671	-0.064923714	-0.182199032	-0.078878151	-0.234342891	-0.017019809	0.466969137	0.047591831	-0.157043115
85	HUMAN_PAPILLOMAVIRUS_INFECTION	0.870684993	0.661738936	0.551762830	0.618538393	0.795759367	0.657970163	0.753816014	0.861819044	0.801214659	0.788844742	-0.173324289	0.358788202	0.726478034	0.709697543
86	HUNTINGTONS_DISEASE	0.684196046	0.858709210	0.600240345	0.430537864	0.443704891	-0.124648995	0.772819254	0.725226084	0.785841759	0.559105250	-0.168462675	0.981073828	0.867237817	0.564354347
87	HYPERTROPHIC_CARDIOMYOPATHY_HCM	0.775269041	0.527536294	0.406423511	0.494167349	0.693211895	0.620185860	0.697663265	0.770078959	0.694040706	0.675625949	-0.049020511	0.210116028	0.617148587	0.584205999
88	INOSITOL_PHOSPHATE_METABOLISM	-0.560990955	-0.644228564	-0.210082256	-0.053780284	-0.171558627	0.371269454	-0.876626562	-0.639182720	-0.658808296	-0.293834390	-0.328293670	-0.764138650	-0.771092502	-0.246079129
89	INSULIN_SIGNALING_PATHWAY	0.989293577	0.906862626	0.847747347	0.854245721	0.949441406	0.672851693	0.781785858	0.970833873	0.957765266	0.968828809	-0.475347495	0.679909076	0.896730427	0.932730895
90	INTESTINAL_IMMUNE_NETWORK_FOR_IGA_PRODUCTION	0.737543297	0.475610534	0.422698803	0.534331611	0.717045418	0.713887257	0.596443687	0.718605059	0.639999067	0.681448502	-0.133445327	0.129555843	0.542357836	0.597030683
91	JAK_STAT_SIGNALING_PATHWAY	0.533799104	0.231308423	0.117877916	0.243151179	0.461850771	0.530016414	0.515671398	0.531904701	0.433186883	0.421507272	0.155806502	-0.095635441	0.352677708	0.316872539
92	LEISHMANIA_INFECTION	0.737345382	0.479302965	0.471502233	0.591104011	0.756575681	0.777670090	0.547141299	0.709079330	0.633753781	0.713226104	-0.219557587	0.121901869	0.524628453	0.637065793
93	LEUKOCYTE_TRANSENDOTHELIAL_MIGRATION	0.594672779	0.304040693	0.334276939	0.483661987	0.649643464	0.775372862	0.403169498	0.561313704	0.473959130	0.586288207	-0.159386282	-0.070240482	0.353309786	0.507576718
94	LIMONENE_AND_PINENE_DEGRADATION	-0.107884503	-0.080723653	-0.542727275	-0.657912124	-0.521088199	-0.803034951	0.424068672	-0.006895290	-0.019565564	-0.427632942	0.887609589	0.114730116	0.122411216	-0.493047842
95	LINOLEIC_ACID_METABOLISM	0.480327980	0.430718797	0.792237873	0.887741095	0.808326987	0.928249444	-0.044801895	0.389112426	0.396404686	0.739563401	-0.932665652	0.183306586	0.255559404	0.781360131
96	LONG_TERM_DEPRESSION	-0.751986902	-0.828908633	-0.466858022	-0.325253122	-0.428202776	0.125742698	-0.936510227	-0.809421005	-0.833421437	-0.540499622	-0.074416447	-0.886083402	-0.913295271	-0.501730691
97	LONG_TERM_POTENTIATION	-0.858153678	-0.872321527	-0.537164724	-0.432925808	-0.564197494	-0.057459203	-0.985316090	-0.904738558	-0.910363104	-0.658207381	-0.017860949	-0.845845796	-0.959755440	-0.605760667
98	LYSINE_DEGRADATION	-0.134389746	0.190340287	0.079136724	-0.118469828	-0.261545979	-0.624802991	0.017296701	-0.095854972	0.007849858	-0.160091466	-0.038290487	0.541616435	0.134711999	-0.086396084
99	LYSOSOME	0.689533636	0.418019041	0.418442614	0.548399116	0.716942057	0.772924582	0.503865421	0.660420735	0.579996737	0.667106948	-0.188539342	0.054164481	0.467573450	0.588659249
100	MAPK_SIGNALING_PATHWAY	0.593694481	0.302348419	0.174646153	0.289171262	0.508464492	0.536404551	0.574316734	0.593493056	0.499032127	0.475535414	0.130043663	-0.020343020	0.422197807	0.371531367
101	MAPK_SIGNALING_PATHWAY.1	0.543373561	0.238372265	0.216072254	0.362817371	0.552621004	0.677288623	0.423496959	0.521989880	0.426534876	0.494232938	-0.014286808	-0.122530797	0.319447249	0.403341035
102	MATURITY_ONSET_DIABETES_OF_THE_YOUNG	-0.794364809	-0.895322217	-0.590245943	-0.447253552	-0.519371635	0.037857355	-0.904706654	-0.839483212	-0.874919146	-0.628072947	0.082634342	-0.947879708	-0.943125016	-0.604927475
103	MELANOGENESIS	0.365135389	0.118728438	-0.205238826	-0.153144867	0.100488762	0.033998069	0.621584822	0.414174720	0.322480304	0.110490987	0.596255754	-0.059115192	0.325221694	-0.011283519
104	MELANOMA	-0.030375001	-0.349552772	-0.418301206	-0.259728132	-0.064433547	0.234601231	0.059165327	-0.026279154	-0.138826143	-0.134408302	0.471955446	-0.607801443	-0.204928279	-0.234538462
105	METABOLISM_OF_XENOBIOTICS_BY_CYTOCHROME_P450	0.428046411	0.111189107	0.019900691	0.160587493	0.376382225	0.508132898	0.415345759	0.423990737	0.319438389	0.324911837	0.201442712	-0.217493480	0.234737719	0.220163977
106	MISMATCH_REPAIR	-0.279338328	0.041732510	-0.059441251	-0.249156924	-0.398100962	-0.703603691	-0.098424480	-0.238938111	-0.138634596	-0.303815893	0.044937799	0.411699089	-0.007496382	-0.229017831
107	MTOR_SIGNALING_PATHWAY	0.414272061	0.164229870	-0.148986221	-0.093761739	0.159761891	0.087622815	0.645513633	0.459537331	0.367943802	0.168190284	0.547214776	-0.028986215	0.363969433	0.046926201
108	N_GLYCAN_BIOSYNTHESIS	0.722553973	0.745781241	0.337886866	0.215918137	0.362461258	-0.154634076	0.966159634	0.788560831	0.791708196	0.468918282	0.232735784	0.775051963	0.871354844	0.407947316
109	NATURAL_KILLER_CELL_MEDIATED_CYTOTOXICITY	0.518542585	0.209827094	0.148541785	0.289223392	0.493344323	0.605641493	0.448020644	0.505914807	0.407058466	0.440827192	0.077358132	-0.137225951	0.311190537	0.342792469
110	NEUROACTIVE_LIGAND_RECEPTOR_INTERACTION	-0.791735330	-0.920176650	-0.663274314	-0.516196593	-0.557168430	-0.002361854	-0.849843349	-0.827779244	-0.874644132	-0.663543207	0.196796429	-0.981958510	-0.937074231	-0.655753234
111	NEUROTROPHIN_SIGNALING_PATHWAY	0.378065395	0.053534583	0.033070407	0.195001230	0.389664767	0.590165362	0.302274753	0.360286403	0.255290693	0.320997959	0.113833477	-0.296657564	0.151570098	0.225540470
112	NICOTINATE_AND_NICOTINAMIDE_METABOLISM	0.871655103	0.776472626	0.452579969	0.409839240	0.597085736	0.206726365	0.985003174	0.911658095	0.880659563	0.661073601	0.099926522	0.645875151	0.896385389	0.579766850
113	NITROGEN_METABOLISM	-0.494522754	-0.323636180	0.082248263	0.092691742	-0.151438835	0.087447810	-0.802833180	-0.559748106	-0.492608305	-0.200652291	-0.581213499	-0.217949081	-0.531516650	-0.086518863
114	NOD_LIKE_RECEPTOR_SIGNALING_PATHWAY	0.582587259	0.284356443	0.260683512	0.402457216	0.590262361	0.694130311	0.453230232	0.560620260	0.468118059	0.535046426	-0.045153344	-0.076861484	0.361074812	0.445686822
115	NON_HOMOLOGOUS_END_JOINING	-0.180961449	0.139950885	0.012114751	-0.185957350	-0.322828137	-0.677207392	0.001771078	-0.137751919	-0.036902905	-0.220507534	0.027360595	0.501407752	0.096139383	-0.150352165
116	NON_SMALL_CELL_LUNG_CANCER	-0.756107328	-0.633356776	-0.838635606	-0.931951741	-0.953152868	-0.960685045	-0.326192210	-0.687325575	-0.667782907	-0.903954350	0.759037929	-0.323765074	-0.535158176	-0.899442970

	Pathways	Erismodegib	Vismodegib	Temsirolimus	Ibrutinib	RXDX-101	Ruxolitinib	Alectinib	Vemurafenib	Dabrafenib	Nilotinib	Dasatinib	Bosutinib	ABT-199	Dovitinib
59	FOLATE_BIOSYNTHESIS	−0.186699719	−0.405981531	−0.130501245	−0.127307688	0.024989506	−0.118464543	−0.837734300	−0.218366276	0.178205054	−0.509268285	−0.692678664	0.152248392	−0.553512894	−0.307522713
60	FRUCTOSE_AND_MANNOSE_METABOLISM	0.211271993	0.136249642	0.548169617	0.230195454	0.488530041	0.490747005	−0.460357762	0.362823852	0.730752663	−0.104184048	−0.245507854	0.651576542	−0.077992947	0.175540177
61	GALACTOSE_METABOLISM	0.957425343	0.961436290	0.880739173	0.942234173	0.982895891	0.978625576	0.620193229	0.993805651	0.907730795	0.871039564	0.789391606	0.951558712	0.885433056	0.972900520
62	GAP_JUNCTION	0.024116148	−0.294874141	−0.500186898	0.101556940	0.004163337	−0.298099665	−0.433624156	−0.271804521	−0.156921778	−0.167439878	−0.367825684	−0.039150782	−0.291792705	−0.150587272
63	GLIOMA	−0.508872241	−0.602694270	−0.141711870	−0.474685644	−0.252705297	−0.262469546	−0.947446093	−0.400074542	0.037199233	−0.767887702	−0.857356542	−0.068786749	−0.758865737	−0.564912662
64	GLUTATHIONE_METABOLISM	0.761097815	0.794324951	0.335270442	0.738437057	0.543791809	0.503855538	0.959967749	0.632509802	0.242089817	0.927927271	0.947621397	0.366928986	0.905484047	0.787712689
65	GLYCEROLIPID_METABOLISM	0.973103897	0.865311053	0.678879845	0.984687044	0.972599409	0.857124899	0.506844360	0.891166639	0.819824321	0.836334493	0.689095525	0.913355800	0.803587559	0.927273263
66	GLYCEROPHOSPHOLIPID_METABOLISM	0.968316621	0.996754108	0.821659280	0.946148440	0.932595190	0.934253206	0.773381225	0.978964164	0.801090461	0.952728778	0.902664147	0.863089829	0.964802509	0.996774921
67	GLYCINE_SERINE_AND_THREONINE_METABOLISM	0.816353886	0.609049622	0.464020976	0.855251384	0.858032652	0.663958445	0.172624385	0.677334463	0.724820240	0.587128340	0.377297407	0.825084813	0.523434226	0.714223430
68	GLYCOLYSIS_GLUCONEOGENESIS	0.812237503	0.695247001	0.739699028	0.830205006	0.935750434	0.847884595	0.162172299	0.816031683	0.929584019	0.567815372	0.393527034	0.964539965	0.553865248	0.760296983
69	GLYCOSAMINOGLYCAN_BIOSYNTHESIS_CHONDROITIN_SULFATE	0.999178136	0.934872060	0.682894416	0.999052155	0.949540085	0.860744305	0.678121304	0.917832096	0.759898651	0.933632855	0.825618845	0.860953883	0.908205880	0.977094432
70	GLYCOSAMINOGLYCAN_BIOSYNTHESIS_HEPARAN_SULFATE	0.209177834	0.013595189	0.277744083	0.256975986	0.437445188	0.314351058	−0.558464131	0.216638013	0.575538087	−0.140231255	−0.344975207	0.557695167	−0.169028992	0.106889714
71	GLYCOSAMINOGLYCAN_BIOSYNTHESIS_KERATAN_SULFATE	0.838757473	0.621799064	0.351431101	0.878754262	0.812587869	0.590781531	0.293660094	0.639008170	0.599998763	0.663361987	0.464230368	0.733447109	0.583275236	0.731025719
72	GLYCOSAMINOGLYCAN_DEGRADATION	0.656004449	0.389589396	0.013402519	0.710066319	0.569654556	0.288398714	0.199242518	0.363613035	0.291145180	0.515622624	0.316175699	0.456030061	0.403817743	0.518128088
73	GLYCOSPHINGOLIPID_BIOSYNTHESIS_GANGLIO_SERIES	0.699587743	0.435733189	0.132155271	0.752966559	0.657798264	0.390334895	0.154137580	0.444553366	0.423451074	0.517377406	0.303786251	0.571226449	0.415396214	0.564112503
74	GLYCOSPHINGOLIPID_BIOSYNTHESIS_GLOBO_SERIES	0.555871839	0.403382599	−0.172280953	0.581425129	0.311838451	0.095238668	0.583326003	0.240291307	−0.075992093	0.639992495	0.564861885	0.110977156	0.540434855	0.477649627
75	GLYCOSPHINGOLIPID_BIOSYNTHESIS_LACTO_AND_NEOLACTO_SERIES	−0.697452563	−0.884170033	−0.750564763	−0.640049917	−0.616864236	−0.757672967	−0.907384468	−0.808974371	−0.534712673	−0.844497650	−0.929815130	−0.546245002	−0.905670929	−0.808573101
76	GLYCOSYLPHOSPHATIDYLINOSITOL_GPI_ANCHOR_BIOSYNTHESIS	−0.452181223	−0.699983254	−0.866354498	−0.385408367	−0.507745409	−0.746091165	−0.590069437	−0.719542167	−0.624605544	−0.532184585	−0.638850111	−0.543731215	−0.640364577	−0.594676203
77	GLYOXYLATE_AND_DICARBOXYLATE_METABOLISM	−0.079307927	0.040159149	0.597797927	−0.103532150	0.200855425	0.389146539	−0.369515246	0.241714953	0.561386377	−0.250580568	−0.244934488	0.398194943	−0.152646968	−0.012365474
78	GNRH_SIGNALING_PATHWAY	0.747905128	0.666519594	0.129929626	0.753348586	0.519576283	0.370332075	0.795950401	0.509709425	0.158241316	0.850914911	0.801434119	0.325743622	0.782874579	0.711416611
79	GRAFT_VERSUS_HOST_DISEASE	0.889171829	0.716214109	0.322140472	0.916529190	0.781936805	0.578243131	0.539099542	0.666073268	0.495795118	0.812684205	0.659933904	0.651479036	0.734232576	0.805511041
80	HEDGEHOG_SIGNALING_PATHWAY	−0.726627287	−0.469747568	−0.185321862	−0.778037867	−0.695867965	−0.437459034	−0.163815852	−0.486644680	−0.473407855	−0.537080627	−0.322731808	−0.615751470	−0.440067690	−0.595443611
81	HEMATOPOIETIC_CELL_LINEAGE	0.663401401	0.428482843	−0.041346377	0.708781944	0.513879765	0.244640363	0.364297705	0.349919816	0.180324748	0.600467982	0.437951046	0.361619193	0.488613224	0.542317936
82	HERPES_SIMPLEX_VIRUS_1_INFECTION	0.914788162	0.782277995	0.363531854	0.931521031	0.782950807	0.609774833	0.661936632	0.708595048	0.485004819	0.885277802	0.763093241	0.638870712	0.817665246	0.853424418
83	HISTIDINE_METABOLISM	0.120099075	−0.124765277	−0.611260175	0.175517512	−0.077672361	−0.360787431	0.060612054	−0.246948723	−0.408127186	0.131901614	0.020117105	−0.234857805	0.002537202	−0.014276263
84	HOMOLOGOUS_RECOMBINATION	−0.391871870	−0.078353704	0.179259046	−0.462331375	−0.368185048	−0.064267791	0.165448627	−0.102918691	−0.165415455	−0.181984199	0.043528696	−0.304390310	−0.062073088	−0.225145130
85	HUMAN_PAPILLOMAVIRUS_INFECTION	0.930545101	0.784659740	0.408540866	0.950719463	0.828637320	0.650906265	0.599354840	0.735970197	0.555339108	0.864025406	0.723780171	0.702031953	0.797183593	0.862335766
86	HUNTINGTONS_DISEASE	0.530530291	0.762743358	0.647283245	0.464310115	0.433817996	0.615135047	0.890297287	0.667326788	0.372084419	0.733381595	0.865683147	0.365269029	0.807306718	0.664168203
87	HYPERTROPHIC_CARDIOMYOPATHY_HCM	0.853351319	0.668246797	0.249487987	0.884045041	0.732784873	0.514742170	0.519582055	0.608745827	0.431020886	0.783522633	0.629565050	0.594344217	0.697632908	0.762423506
88	INOSITOL_PHOSPHATE_METABOLISM	−0.415926526	−0.581924340	−0.218856304	−0.365861809	−0.187335517	−0.273110878	−0.944425757	−0.390742982	0.031857272	−0.703341231	−0.838856069	−0.032647312	−0.726116045	−0.510512740
89	INSULIN_SIGNALING_PATHWAY	0.996681405	0.968518321	0.758951020	0.987928397	0.960397578	0.908856050	0.707744582	0.957457989	0.800883626	0.942741236	0.854358909	0.883931122	0.933519375	0.994496087
90	INTESTINAL_IMMUNE_NETWORK_FOR_IGA_PRODUCTION	0.837955407	0.627820699	0.261339091	0.875508690	0.754055629	0.522938793	0.405860731	0.599498262	0.482550593	0.719535860	0.540723763	0.637984664	0.631027322	0.733429994
91	JAK_STAT_SIGNALING_PATHWAY	0.644780821	0.396411022	−0.052947545	0.693934220	0.509761578	0.232245614	0.305283845	0.330433082	0.188012757	0.561174631	0.387567287	0.366656671	0.446834729	0.516201874
92	LEISHMANIA_INFECTION	0.845621099	0.632585960	0.312283237	0.884151190	0.789901401	0.563616128	0.356755789	0.626537228	0.546548415	0.699170047	0.510127944	0.691529727	0.614996373	0.739865626
93	LEUKOCYTE_TRANSENDOTHELIAL_MIGRATION	0.729732442	0.474201263	0.166951025	0.780583765	0.685598873	0.424765848	0.191721595	0.480623092	0.449031790	0.553007453	0.342952150	0.596739642	0.453983096	0.599209086
94	LIMONENE_AND_PINENE_DEGRADATION	−0.218592228	−0.129853418	−0.527386488	−0.240712892	−0.492258592	−0.480638727	0.469829670	−0.355388888	−0.723937862	0.103048146	0.252742545	−0.651345529	0.082672319	−0.175409078
95	LINOLEIC_ACID_METABOLISM	0.572783618	0.493020254	0.747045255	0.586525275	0.787854573	0.763161137	−0.108382692	0.676963542	0.921973714	0.285892496	0.129507104	0.891739534	0.301470913	0.538815554
96	LONG_TERM_DEPRESSION	−0.621741069	−0.779224386	−0.463570356	−0.571254333	−0.438935009	−0.526015536	−0.991996633	−0.627337882	−0.242801167	−0.847833401	−0.948705283	−0.302904010	−0.877832170	−0.714879410
97	LONG_TERM_POTENTIATION	−0.761908369	−0.860310118	−0.498985033	−0.723783011	−0.581383831	−0.611989590	−0.994523787	−0.719185266	−0.347056350	−0.937755798	−0.988925942	−0.434378359	−0.946998439	−0.824592343
98	LYSINE_DEGRADATION	−0.312540214	0.006513305	0.239179953	−0.385697204	−0.298886200	0.006217890	0.246290162	−0.025395687	−0.113137026	−0.095380264	0.130036563	−0.245120890	0.025379663	−0.141789261
99	LYSOSOME	0.807040614	0.578001793	0.255268160	0.850081285	0.751822748	0.511400434	0.304937818	0.573728679	0.506331593	0.652350065	0.456049361	0.654263552	0.561890520	0.691991739
100	MAPK_SIGNALING_PATHWAY	0.695023336	0.461836369	0.006031345	0.739635462	0.555510514	0.290103919	0.370960301	0.391249942	0.229995301	0.621947283	0.455232288	0.408330660	0.512919774	0.575410590
101	MAPK_SIGNALING_PATHWAY.1	0.675050258	0.411389762	0.044154891	0.728227259	0.594500659	0.317082246	0.206265306	0.389836227	0.321324439	0.529883021	0.329064638	0.483943972	0.420385485	0.538995851
102	MATURITY_ONSET_DIABETES_OF_THE_YOUNG	−0.663969267	−0.837741473	−0.596608170	−0.609416655	−0.522552840	−0.635345877	−0.972480106	−0.716534056	−0.373225349	−0.860161286	−0.956647790	−0.412830670	−0.904565201	−0.766986610
103	MELANOGENESIS	0.406935473	0.235506535	−0.342382418	0.439294456	0.155958681	−0.077808645	0.464252734	0.067264129	−0.231246960	0.495259073	0.421940786	−0.043640300	0.385961013	0.316239349
104	MELANOMA	0.109520898	−0.184892957	−0.568634846	0.178230886	−0.014565492	−0.323573365	−0.152758090	−0.244231022	−0.286105816	0.022764926	−0.140128725	−0.127755421	−0.111407339	−0.051490996
105	METABOLISM_OF_XENOBIOTICS_BY_CYTOCHROME_P450	0.552847112	0.283160860	−0.152328025	0.608717264	0.425284781	0.131412124	0.196308362	0.224248807	0.112384234	0.454884844	0.273009793	0.289727641	0.333125861	0.411398650
106	MISMATCH_REPAIR	−0.450676436	−0.142644299	0.106155553	−0.519007093	−0.434925903	−0.137345192	0.130678562	−0.173504211	−0.236626941	−0.233108713	−0.004176724	−0.373833833	−0.117279033	−0.287829983
107	MTOR_SIGNALING_PATHWAY	0.459172202	0.284781684	−0.290235883	0.491551228	0.214759342	−0.020792853	0.484073747	0.122692815	−0.172362593	0.536005628	0.454774977	0.016529865	0.427274675	0.367623967
108	N_GLYCAN_BIOSYNTHESIS	0.608925907	0.720865921	0.310304700	0.569209654	0.384083967	0.417629196	0.988376937	0.541705500	0.124679754	0.844404378	0.928765200	0.217170639	0.849047099	0.676544666
109	NATURAL_KILLER_CELL_MEDIATED_CYTOTOXICITY	0.644090320	0.381553026	−0.024146241	0.697123448	0.538578068	0.255912110	0.230920064	0.340110620	0.242473525	0.524473424	0.333741423	0.413429971	0.410386012	0.508003549
110	NEUROACTIVE_LIGAND_RECEPTOR_INTERACTION	−0.658519406	−0.851252814	−0.683110275	−0.599898877	−0.553141844	−0.693421615	−0.933690013	−0.755607497	−0.451218039	−0.834696454	−0.933823464	−0.468892683	−0.893098226	−0.772182412
111	NEUROTROPHIN_SIGNALING_PATHWAY	0.522854021	0.233439745	−0.139858877	0.584548725	0.434962161	0.133985654	0.075281337	0.207197329	0.159410102	0.378032595	0.175042872	0.324950757	0.256111868	0.370542600
112	NICOTINATE_AND_NICOTINAMIDE_METABOLISM	0.830379302	0.823833534	0.361924323	0.816614661	0.628820081	0.555112396	0.919203440	0.679923443	0.319382235	0.952193175	0.936538754	0.455088183	0.918274393	0.836279770
113	NITROGEN_METABOLISM	−0.479598640	−0.397294903	0.190534509	−0.488666776	−0.201350467	−0.041885531	−0.698320905	−0.198260923	0.184729571	−0.645835073	−0.631143889	0.011540421	−0.565688747	−0.438498586
114	NOD_LIKE_RECEPTOR_SIGNALING_PATHWAY	0.709918885	0.454535096	0.090014389	0.760563376	0.631095479	0.361294942	0.239813740	0.433805408	0.359531875	0.566182191	0.367368421	0.520659384	0.460438840	0.578892029
115	NON_HOMOLOGOUS_END_JOINING	−0.359933505	−0.044031305	0.172563421	−0.431761486	−0.358249787	−0.058584660	0.230084670	−0.085482034	−0.181538064	−0.130689280	0.099829628	−0.310176572	−0.013799877	−0.191664152
116	NON_SMALL_CELL_LUNG_CANCER	−0.844675371	−0.729822023	−0.744009415	−0.861413404	−0.952518555	−0.861881903	−0.215546381	−0.838661631	−0.926928483	−0.613658082	−0.442434154	−0.969663056	−0.597803546	−0.794206330

	Pathways	cluster	Afatinib	Erlotinib HCl	Dacomitinib	Gefitinib	Lapatinib	Neratinib	CO-1686	Everolimus	BYL719	Pilaralisib	XL765	Pazopanib HCl	Sunitinib Malate
117	NOTCH_SIGNALING_PATHWAY	C1	−0.792358136	−0.551570489	−0.997038380	−0.499713550	−0.865041827	−0.916866927	−0.921017983	−0.852126680	−0.765632940	−0.836272482	−0.781781762	−0.686240667	−0.571204894
118	NUCLEOTIDE_EXCISION_REPAIR	C3	−0.117532570	0.108628096	0.114692327	0.483648678	0.492252282	−0.107942852	−0.230737162	0.456962727	0.493668284	0.289908654	0.544494577	0.169829237	−0.183366139
119	O_GLYCAN_BIOSYNTHESIS	C3	0.091045040	−0.293775538	0.181232685	−0.624041126	−0.368845225	0.219128583	0.444053465	−0.363549409	−0.465283287	−0.259998439	−0.481398347	−0.270171651	0.005459032
120	OLFACTORY_TRANSDUCTION	C3	−0.230918123	−0.301259241	−0.536386038	−0.591126233	−0.774752358	−0.303404204	−0.214031524	−0.737670296	−0.725700006	−0.588282285	−0.777297043	−0.415337302	−0.070855955
121	ONE_CARBON_POOL_BY_FOLATE	C3	0.147250090	0.368044842	0.281252738	0.694424119	0.689940528	0.129107655	−0.038215965	0.666102765	0.706039680	0.529545262	0.743704159	0.428769591	0.087078442
122	OOCYTE_MEIOSIS	C1	−0.295138361	−0.079296854	0.001320819	0.312942475	0.336119284	−0.263777147	−0.347920552	0.293891079	0.325209059	0.112100511	0.383541740	−0.018677618	−0.365252354
123	OTHER_GLYCAN_DEGRADATION	C3	0.593774947	0.314856216	0.363900662	−0.075314294	0.019485964	0.598919587	0.667280905	0.056928689	0.000577976	0.226626620	−0.051065082	0.302817045	0.587564295
124	OXIDATIVE_PHOSPHORYLATION	C2	0.961279090	0.979333076	0.661809537	0.878640455	0.846681792	0.867726596	0.681940198	0.880319312	0.896304830	0.955608750	0.859796776	0.995142425	0.960040142
125	P53_SIGNALING_PATHWAY	C1	−0.524504855	−0.531820796	0.052299872	−0.204709315	0.056218719	−0.351093829	−0.254781767	−0.008813577	−0.051689947	−0.207813484	0.028938463	−0.421794915	−0.704876287
126	PANCREATIC_CANCER	C3	0.355693211	−0.011784475	0.859199175	−0.033768026	0.500088773	0.602310418	0.764140380	0.458293746	0.322254465	0.389776517	0.369616057	0.158749447	0.053458109
127	PANTOTHENATE_AND_COA_BIOSYNTHESIS	C2	0.723606415	0.341688534	0.861065128	0.095236419	0.478408658	0.865205638	0.971485108	0.478670097	0.364741193	0.542262771	0.359669492	0.445500064	0.529293107
128	PARKINSONS_DISEASE	C2	0.927831814	0.954239556	0.715341241	0.916219914	0.926324368	0.857956667	0.672334877	0.949806379	0.960964872	0.990115850	0.937630704	0.991734181	0.893844976
129	PATHOGENIC_ESCHERICHIA_COLI_INFECTION	C2	0.964694626	0.755220180	0.803127134	0.510418994	0.671332644	0.972026851	0.927799740	0.698539714	0.647972456	0.804605082	0.614005493	0.803939536	0.882800772
130	PATHWAYS_IN_CANCER	C3	0.552687815	0.140509442	0.653189769	−0.174711451	0.173905118	0.691190319	0.848300375	0.179431073	0.063717833	0.273790520	0.049833769	0.214805031	0.395803126
131	PENTOSE_AND_GLUCURONATE_INTERCONVERSIONS	C3	0.485281142	0.074029122	0.566962715	−0.257837893	0.068380984	0.617916437	0.787006478	0.075946870	−0.037009545	0.179346363	−0.053991956	0.135862384	0.346133179
132	PENTOSE_PHOSPHATE_PATHWAY	C2	0.925847478	0.970128079	0.676197774	0.926126586	0.905220796	0.840212030	0.644006746	0.932172976	0.950797512	0.980119387	0.923399628	0.997305901	0.909979207
133	PEROXISOME	C2	0.973418823	0.970135572	0.627383282	0.818067207	0.770869320	0.871623927	0.697435656	0.810614921	0.825196150	0.910993803	0.780290257	0.973209377	0.989055618
134	PHENYLALANINE_METABOLISM	C2	0.649705492	0.327900815	0.981706344	0.264359757	0.721603772	0.833366849	0.913747098	0.697493665	0.582753186	0.667658329	0.610369062	0.481585629	0.385608726
135	PHOSPHATIDYLINOSITOL_SIGNALING_SYSTEM	C3	0.024302066	0.171519139	−0.570131315	−0.062562823	−0.482174342	−0.191317890	−0.283559251	−0.421076293	−0.335902550	−0.250012642	−0.411332424	0.008110105	0.294309218
136	PI3KAKT_SIGNALING_PATHWAY	C3	0.513060449	0.155165119	0.443459325	−0.218205739	−0.003046183	0.586648588	0.718278159	0.019447181	−0.068302498	0.159852636	−0.103114939	0.178172241	0.440992928
137	PORPHYRIN_AND_CHLOROPHYLL_METABOLISM	C3	0.516992058	0.108269233	0.593768557	−0.221685074	0.105483256	0.647512925	0.809214361	0.113521017	0.001140273	0.216807759	−0.016168299	0.172110161	0.375789818
138	PPAR_SIGNALING_PATHWAY	C3	0.511188487	0.142746488	0.467336088	−0.224843344	0.010389492	0.595212935	0.733615799	0.030402629	−0.062031169	0.164951156	−0.093905046	0.172617360	0.427178280
139	PRIMARY_BILE_ACID_BIOSYNTHESIS	C3	0.276061081	−0.135412802	0.378697727	−0.470382524	−0.165190798	0.414921378	0.622481974	−0.160111823	−0.271933674	−0.057661426	−0.287155546	−0.089590687	0.156894932
140	PRIMARY_IMMUNODEFICIENCY	C3	0.448858589	0.008069791	0.672567667	−0.264482872	0.154342735	0.631518805	0.824731055	0.147065192	0.015550513	0.208543352	0.016839511	0.105898254	0.249957844
141	PRION_DISEASES	C3	0.125026153	−0.216459147	0.098884347	−0.575266668	−0.404569231	0.207102730	0.399446490	−0.387879026	−0.469851453	−0.257134011	−0.499420135	−0.221311869	0.089461054
142	PROGESTERONE_MEDIATED_OOCYTE_MATURATION	C1	−0.554362761	−0.449800601	−0.081949976	−0.077911434	0.062005300	−0.441454906	−0.409256501	0.004238588	−0.002439131	−0.195068204	0.071411169	−0.369415082	−0.675102872
143	PROPANOATE_METABOLISM	C3	−0.051551506	0.375000797	−0.592822754	0.457232775	−0.078962611	−0.324843935	−0.579550888	−0.035080490	0.114101794	0.016091052	0.067673436	0.227455758	0.229928362
144	PROSTATE_CANCER	C3	0.542077757	0.195226747	0.446998798	−0.181090320	0.018265859	0.605188368	0.724732845	0.043206510	−0.040144531	0.188285111	−0.077649939	0.214319443	0.478090083
145	PROTEASOME	C2	0.749637301	0.781175873	0.763099237	0.881686300	0.994485825	0.745598549	0.587648246	0.992190237	0.988097931	0.950716065	0.997137382	0.864886688	0.641881472
146	PROTEIN_DIGESTION_AND_ABSORPTION	C3	0.326222110	−0.114536696	0.530817450	−0.409215477	−0.021378991	0.503145153	0.719069416	−0.026583278	−0.154753940	0.046203044	−0.157016227	−0.035093723	0.152814436
147	PROTEIN_EXPORT	C3	0.102870502	0.507364486	−0.158215302	0.761782997	0.426935783	−0.081183405	−0.352760875	0.440232350	0.558351253	0.387960937	0.552173902	0.455752904	0.238041209
148	PROXIMAL_TUBULE_BICARBONATE_RECLAMATION	C1	−0.383323492	0.066403781	−0.665958806	0.314918319	−0.132632545	−0.585849710	−0.796261243	−0.119031376	0.018671686	−0.163572793	0.009855326	−0.041155193	−0.166953862
149	PURINE_METABOLISM	C1	−0.623336695	−0.884802523	−0.040346030	−0.823180670	−0.450792547	−0.381273032	−0.106708851	−0.506415856	−0.609750607	−0.611493198	−0.548557983	−0.791223970	−0.814650293
150	PYRIMIDINE_METABOLISM	C3	−0.027631089	0.276440393	0.036879164	0.629437815	0.515268229	−0.088416138	−0.275435775	0.495815014	0.564158591	0.360965153	0.597038905	0.301394729	−0.025718948
151	PYRUVATE_METABOLISM	C3	−0.217820166	0.092310878	−0.098382756	0.472329679	0.357238417	−0.263915598	−0.420209058	0.331231668	0.399100350	0.179603025	0.439318813	0.113193867	−0.212932992
152	REGULATION_OF_ACTIN_CYTOSKELETON	C3	0.519576270	0.139180428	0.507343857	−0.219438071	0.041672972	0.616157884	0.760283119	0.058607302	−0.039488629	0.185244471	−0.067610560	0.178635313	0.419495683
153	REGULATION_OF_AUTOPHAGY	C3	0.494344407	0.057966713	0.784875103	−0.162877172	0.300650737	0.695746259	0.877248412	0.285697394	0.148986317	0.318679574	0.160679081	0.179883069	0.260180343
154	RENAL_CELL_CARCINOMA	C3	0.505656686	0.104303261	0.555203395	−0.236713581	0.068933182	0.626131727	0.785504718	0.079736661	−0.028415909	0.191042717	−0.049207919	0.159368133	0.378613897
155	RENIN_ANGIOTENSIN_SYSTEM	C2	0.612679492	0.209604064	0.701706063	−0.098983490	0.248028688	0.745392642	0.885822677	0.254521958	0.141124512	0.347921866	0.126637255	0.287200910	0.453245370
156	RETINOL_METABOLISM	C3	0.328527654	−0.046013727	0.318757348	−0.412099783	−0.183006228	0.424238524	0.599816707	−0.166706686	−0.260902758	−0.036820977	−0.289387276	−0.025775133	0.254528802
157	RIBOFLAVIN_METABOLISM	C1	−0.454275269	−0.763281971	−0.191546478	−0.946078212	−0.713045570	−0.294963894	−0.018882206	−0.730574580	−0.820833111	−0.702232712	−0.809167528	−0.745236019	−0.532867684
158	RIBOSOME	C3	0.271856573	−0.180787882	0.673101318	−0.363778213	0.137536042	0.510412001	0.742183198	0.110616365	−0.036193447	0.114873630	−0.010929267	−0.051027630	0.018778359
159	RIG_I_LIKE_RECEPTOR_SIGNALING_PATHWAY	C2	0.759880425	0.431353582	0.670239350	0.094667998	0.323244447	0.819862518	0.887731377	0.346979568	0.264521854	0.476076207	0.230577502	0.475487774	0.664709211
160	RNA_DEGRADATION	C1	−0.476695570	−0.202964215	−0.242212048	0.194124681	0.123345468	−0.479065087	−0.565745038	0.085666458	0.138726707	−0.087760288	0.191363301	−0.177443248	−0.489289657
161	RNA_POLYMERASE	C1	−0.472676359	−0.039513314	−0.814862978	0.146050855	−0.343018388	−0.688598709	−0.871630983	−0.322102516	−0.182128677	−0.335009293	−0.201498555	−0.174064749	−0.219164038
162	SELENOAMINO_ACID_METABOLISM	C3	0.285037548	0.671573879	−0.083880715	0.851587983	0.490846347	0.073675656	−0.219212724	0.517128068	0.637412875	0.506345836	0.615180212	0.607648880	0.440519213
163	SMALL_CELL_LUNG_CANCER	C2	0.770498798	0.410314891	0.908581282	0.190356721	0.570842090	0.906582986	0.990534455	0.570193802	0.460796058	0.624043982	0.457792095	0.519908410	0.571252728
164	SNARE_INTERACTIONS_IN_VESICULAR_TRANSPORT	C3	0.337794274	−0.103786211	0.545682289	−0.396249399	−0.004208881	0.515917212	0.730201740	−0.009743808	−0.138552848	0.061709614	−0.140325644	−0.022206458	0.161257174
165	SPHINGOLIPID_METABOLISM	C3	−0.113040817	−0.277829021	−0.348241769	−0.609817702	−0.687003264	−0.139734825	−0.013143922	−0.653982782	−0.672376090	−0.500621610	−0.720294186	−0.362256413	−0.008416928
166	SPLICEOSOME	C1	−0.333214638	−0.126291355	−0.015828907	0.267399797	0.301199190	−0.293292605	−0.364984967	0.256945565	0.284888474	0.071732184	0.345357168	−0.063950878	−0.407964805
167	STAPHYLOCOCCUS_AUREUS_INFECTION	C2	0.626444440	0.251881977	0.614202426	−0.092288372	0.183111040	0.721524572	0.842306126	0.199881908	0.101687484	0.320849640	0.074675723	0.302758615	0.511963752
168	STARCH_AND_SUCROSE_METABOLISM	C3	0.515180184	0.116182841	0.557985147	−0.226050980	0.075985351	0.633032323	0.789224672	0.087460265	−0.019691071	0.200149048	−0.041228274	0.170388629	0.389968925
169	STEROID_BIOSYNTHESIS	C2	0.732767594	0.694696953	0.847399958	0.788161881	0.992011334	0.777140440	0.665301467	0.981091645	0.951930941	0.927227785	0.970270694	0.803508270	0.576974153
170	STEROID_HORMONE_BIOSYNTHESIS	C3	0.306410235	−0.105965783	0.404950179	−0.442136755	−0.134066031	0.444298711	0.646876296	−0.128469834	−0.240592845	−0.025138478	−0.256297355	−0.058153041	0.184728071
171	SULFUR_METABOLISM	C2	0.788375811	0.438091392	0.809069858	0.153197299	0.462241919	0.886926532	0.962334179	0.474434864	0.377045944	0.568649390	0.357738153	0.516200952	0.638965035
172	SYSTEMIC_LUPUS_ERYTHEMATOSUS	C3	0.093847294	0.398761483	0.101045307	0.724858652	0.595825992	0.015824519	−0.193603768	0.582874560	0.654528212	0.465458608	0.679833659	0.420085576	0.104014333
173	T_CELL_RECEPTOR_SIGNALING_PATHWAY	C3	0.484404436	0.049349018	0.682833425	−0.230973803	0.175795800	0.658044995	0.841314828	0.171159987	0.042653608	0.238963426	0.040906482	0.143843263	0.292125890
174	TASTE_TRANSDUCTION	C1	−0.133968656	−0.054286749	0.296968040	0.298167941	0.493029075	−0.026720451	−0.055872326	0.441201862	0.426079733	0.252830353	0.494518389	0.057671744	−0.297329271

	Pathways	Axitinib	Tivozanib	Vandetanib	Regorafenib	Selumetinib	Trametinib	Crizotinib	Cabozantinib	Foretinib	Palbociclib HCl	Ribociclib	Abemaciclib	Veliparib	Olaparib
117	NOTCH_SIGNALING_PATHWAY	−0.823315520	−0.779876540	−0.410745308	−0.331052524	−0.503893436	−0.047282401	−0.998218043	−0.875997140	−0.859850201	−0.588591124	−0.162176945	−0.719018821	−0.904947273	−0.514753457
118	NUCLEOTIDE_EXCISION_REPAIR	−0.102381264	0.197455905	−0.018662416	−0.227004108	−0.320405315	−0.733956571	0.155101818	−0.043600746	0.047625215	−0.204080485	0.156886662	0.555410446	0.195125565	−0.154139638
119	O_GLYCAN_BIOSYNTHESIS	0.102430227	−0.228884202	−0.243907823	−0.070110891	0.117870112	0.419482435	0.097047272	0.091066420	−0.020928384	0.038259548	0.296784125	−0.538884410	−0.112676082	−0.057218329
120	OLFACTORY_TRANSDUCTION	−0.258684842	−0.483352211	−0.131157058	0.075925146	0.071728838	0.610988120	−0.565909367	−0.333183248	−0.399276918	−0.062243334	−0.238279529	−0.755788963	−0.544089042	−0.068198827
121	ONE_CARBON_POOL_BY_FOLATE	0.158858198	0.451685096	0.237625845	0.026940358	−0.055254283	−0.546137630	0.342027315	0.209572489	0.303153986	0.066572725	−0.015937767	0.757117193	0.433638505	0.113918535
122	OOCYTE_MEIOSIS	−0.277646100	0.012475432	−0.200305626	−0.398198660	−0.492876819	−0.836591317	0.024846559	−0.214950273	−0.130025340	−0.384881875	0.278959205	0.391182438	0.023650826	−0.336485144
123	OTHER_GLYCAN_DEGRADATION	0.586911240	0.303544123	0.379713689	0.535611860	0.682010104	0.831483246	0.347432477	0.544383996	0.461363270	0.611390744	−0.245343507	−0.078009465	0.330340809	0.542039582
124	OXIDATIVE_PHOSPHORYLATION	0.952737547	0.987261775	0.936736704	0.882628236	0.910351606	0.529479710	0.743663855	0.937610577	0.964211889	0.957192451	−0.585839338	0.868725385	0.934671709	0.955322305
125	P53_SIGNALING_PATHWAY	−0.486697205	−0.337480196	−0.665861356	−0.807841495	−0.793782396	−0.994501880	0.002158621	−0.398764227	−0.370709652	−0.705915677	0.774763725	−0.010561834	−0.211605231	−0.714549437
126	PANCREATIC_CANCER	0.404412872	0.293362967	−0.164646894	−0.216914725	0.009433955	−0.294697944	0.793156276	0.485227733	0.434660295	0.081549940	0.675933597	0.274227127	0.509384443	−0.023790377
127	PANTOTHENATE_AND_COA_BIOSYNTHESIS	0.749034578	0.529409207	0.273245628	0.316243742	0.544505259	0.366335428	0.825060517	0.773922646	0.701164248	0.559601782	0.186260623	0.286282994	0.670971694	0.454684261
128	PARKINSONS_DISEASE	0.925666939	0.998526220	0.881821601	0.792820747	0.821094066	0.367819000	0.795319226	0.926542798	0.962887505	0.889872855	−0.486047166	0.940899658	0.963711622	0.888313903
129	PATHOGENIC_ESCHERICHIA_COLI_INFECTION	0.968851196	0.834188081	0.718005713	0.740613088	0.882650358	0.631407964	0.830122790	0.961117318	0.927369126	0.899018198	−0.304118535	0.582773270	0.869128677	0.839311017
130	PATHWAYS_IN_CANCER	0.572586073	0.283962500	0.120733084	0.226380850	0.455454702	0.465267746	0.597643493	0.580535092	0.485087776	0.429497601	0.206294599	−0.020896199	0.420056605	0.320687768
131	PENTOSE_AND_GLUCURONATE_INTERCONVERSIONS	0.502568654	0.197745218	0.071766478	0.196292751	0.419497827	0.492507359	0.506772956	0.504109827	0.403494147	0.380064373	0.202142537	−0.121708015	0.327579355	0.272897765
132	PENTOSE_PHOSPHATE_PATHWAY	0.920545265	0.995908157	0.907532589	0.823629677	0.840887523	0.404423243	0.760740854	0.915706845	0.954043139	0.904665861	−0.536815033	0.932081992	0.947949916	0.908327817
133	PEROXISOME	0.961486852	0.957998347	0.948725016	0.924230353	0.959019251	0.640638204	0.705867289	0.936119439	0.951680210	0.988300428	−0.628139199	0.790252861	0.899820317	0.982315627
134	PHENYLALANINE_METABOLISM	0.689123203	0.596318787	0.182022415	0.122418190	0.333030674	−0.056356970	0.951241722	0.753349654	0.715988722	0.409782208	0.385506232	0.529484194	0.767676820	0.315446925
135	PHOSPHATIDYLINOSITOL_SIGNALING_SYSTEM	−0.024012901	−0.113088152	0.355277435	0.508808211	0.399359651	0.793081978	−0.525197765	−0.124779097	−0.135335204	0.284789668	−0.746980552	−0.343155909	−0.288606462	0.334374484
136	PI3KAKT_SIGNALING_PATHWAY	0.518292156	0.209574087	0.192204199	0.342701063	0.532091385	0.671960310	0.400058451	0.496452486	0.399494080	0.471046786	−0.002561102	−0.151912905	0.291417509	0.379990403
137	PORPHYRIN_AND_CHLOROPHYLL_METABOLISM	0.534383397	0.234601923	0.102222503	0.221779393	0.445380392	0.498675050	0.536657205	0.536545319	0.437942101	0.409429696	0.187202612	−0.083768592	0.363411821	0.302539979
138	PPAR_SIGNALING_PATHWAY	0.518528674	0.209431090	0.173020926	0.319465481	0.515453002	0.643721661	0.420953413	0.500666007	0.402756984	0.458011126	0.032391799	−0.146161443	0.299862898	0.363822179
139	PRIMARY_BILE_ACID_BIOSYNTHESIS	0.291634119	−0.034748305	−0.112596419	0.039709381	0.251940024	0.446875221	0.302207113	0.288280784	0.178623532	0.191375917	0.278685404	−0.352613241	0.095177347	0.086599775
140	PRIMARY_IMMUNODEFICIENCY	0.477153704	0.194834708	−0.034715936	0.055113662	0.300046789	0.293175024	0.600624756	0.501945165	0.404074385	0.285766486	0.385185340	−0.065867541	0.364526933	0.168533366
141	PRION_DISEASES	0.127329880	−0.202906217	−0.138948125	0.051272873	0.212958659	0.554695020	0.029108605	0.098719582	−0.009039589	0.119039441	0.134842245	−0.542315527	−0.123859851	0.036321932
142	PROGESTERONE_MEDIATED_OOCYTE_MATURATION	−0.526191336	−0.312437635	−0.568186773	−0.727959402	−0.774493840	−0.983908805	−0.106726491	−0.450124991	−0.397582592	−0.685219252	0.603010359	0.055297319	−0.238918872	−0.664106857
143	PROPANOATE_METABOLISM	−0.098772628	0.098765246	0.479152580	0.459434541	0.221652184	0.286700714	−0.489266329	−0.168902927	−0.086703807	0.195949191	−0.828912686	0.167739859	−0.133274825	0.312034121
144	PROSTATE_CANCER	0.545660021	0.241779325	0.234523941	0.383963171	0.568185342	0.700351576	0.408760195	0.521092759	0.426713356	0.507235528	−0.044411751	−0.123476609	0.315616719	0.419039388
145	PROTEASOME	0.762670139	0.905761870	0.649909216	0.495852936	0.527623109	−0.032210136	0.827379217	0.799829551	0.851225691	0.636719139	−0.194070954	0.985403438	0.918970550	0.633302549
146	PROTEIN_DIGESTION_AND_ABSORPTION	0.350992403	0.043621650	−0.130767117	−0.011836782	0.224285868	0.319494977	0.449818001	0.366893355	0.260605143	0.189111504	0.395285597	−0.235725522	0.205902034	0.074097026
147	PROTEIN_EXPORT	0.080787299	0.390667236	0.486667766	0.337950098	0.140125006	−0.148758321	−0.052154358	0.069218765	0.180670305	0.203900336	−0.546165406	0.622804353	0.233851276	0.305573615
148	PROXIMAL_TUBULE_BICARBONATE_RECLAMATION	−0.415343836	−0.138474093	0.118905971	0.036684559	−0.212707779	−0.203075651	−0.586071422	−0.447916522	−0.349598215	−0.203408703	−0.472068817	0.097389697	−0.322718561	−0.083505657
149	PURINE_METABOLISM	−0.584873006	−0.698432735	−0.940640625	−0.918333037	−0.793626062	−0.634727360	−0.157414635	−0.521470181	−0.581192384	−0.793226405	0.952297173	−0.618429431	−0.512173288	−0.861340961
150	PYRIMIDINE_METABOLISM	−0.025015611	0.297444614	0.178612868	−0.022231964	−0.158130489	−0.559770578	0.104781909	0.011947683	0.116497972	−0.052553519	−0.095843036	0.630126021	0.238780074	0.018892441
151	PYRUVATE_METABOLISM	−0.213928411	0.109490187	0.002086557	−0.191948342	−0.338051081	−0.671407225	−0.044363882	−0.173987903	−0.071985431	−0.240020336	0.009775681	0.472208619	0.057887009	−0.165795698
152	REGULATION_OF_ACTIN_CYTOSKELETON	0.529672304	0.222613439	0.159209032	0.298237172	0.502514524	0.608101525	0.458346538	0.517232722	0.419048984	0.451164795	0.072022297	−0.124196533	0.323602793	0.353216013
153	REGULATION_OF_AUTOPHAGY	0.528708106	0.283971841	−0.018430878	0.033435875	0.284000166	0.175474029	0.716446082	0.568653139	0.481382372	0.295078991	0.444875004	0.071992757	0.469477158	0.176597200
154	RENAL_CELL_CARCINOMA	0.520628343	0.215615948	0.108475402	0.236854543	0.454405443	0.533463088	0.499347850	0.517697562	0.418155990	0.411801786	0.155796405	−0.113365565	0.335942257	0.307509281
155	RENIN_ANGIOTENSIN_SYSTEM	0.632568040	0.356871858	0.182396860	0.277002934	0.504691141	0.474289402	0.653023495	0.641634853	0.551186088	0.486024281	0.173133002	0.056851611	0.489468275	0.378824560
156	RETINOL_METABOLISM	0.335678508	0.008243369	0.001684370	0.169368212	0.359632268	0.585797682	0.256533199	0.316077364	0.209935991	0.286266732	0.120035105	−0.341744615	0.103912675	0.192361873
157	RIBOFLAVIN_METABOLISM	−0.438428960	−0.703204810	−0.712257203	−0.559546359	−0.426426913	−0.012430874	−0.302124553	−0.433416436	−0.532073734	−0.505981009	0.582777255	−0.861187536	−0.577272350	−0.579901551
158	RIBOSOME	0.311602535	0.063506834	−0.259922831	−0.205438782	0.047272464	0.000480712	0.582298404	0.361983738	0.268780672	0.055154062	0.633113663	−0.106692687	0.273890738	−0.066810773
159	RIG_I_LIKE_RECEPTOR_SIGNALING_PATHWAY	0.767308171	0.514668091	0.429591574	0.528246932	0.717625083	0.674877926	0.652792183	0.754606242	0.678311500	0.691546123	−0.104357801	0.182357524	0.590496740	0.604508335
160	RNA_DEGRADATION	−0.467525064	−0.170801965	−0.286687364	−0.461720282	−0.598350691	−0.825840485	−0.218101240	−0.419568422	−0.331576784	−0.513053709	0.223592844	0.214999177	−0.192505683	−0.446714819
161	RNA_POLYMERASE	−0.510809928	−0.286857740	0.053864263	0.021378259	−0.230202093	−0.081954170	−0.744774602	−0.560034260	−0.477882890	−0.253553012	−0.505600062	−0.109267712	−0.483298195	−0.134864262
162	SELENOAMINO_ACID_METABOLISM	0.257386178	0.532221264	0.667624890	0.542253102	0.355702969	0.069864553	0.033453297	0.231322430	0.335661759	0.407998493	−0.701356604	0.689148587	0.358507760	0.505476768
163	SMALL_CELL_LUNG_CANCER	0.796587499	0.605358074	0.328691422	0.350502821	0.571704350	0.335519424	0.881212087	0.825430788	0.762331759	0.599514266	0.162563692	0.386159898	0.742933149	0.499110216
164	SNARE_INTERACTIONS_IN_VESICULAR_TRANSPORT	0.363022317	0.057802415	−0.122877280	−0.006822987	0.230614565	0.315692109	0.465324484	0.379979108	0.274367571	0.197571109	0.396445956	−0.219640389	0.221331758	0.082114662
165	SPHINGOLIPID_METABOLISM	−0.132600784	−0.405732952	−0.128775209	0.084608529	0.135841286	0.636966991	−0.391854624	−0.196570749	−0.280230397	0.007672731	−0.136607501	−0.718816304	−0.424316982	−0.024263764
166	SPLICEOSOME	−0.314599904	−0.030324147	−0.246950152	−0.441595885	−0.532957808	−0.860843793	0.002407277	−0.250256884	−0.168154712	−0.426817271	0.316872603	0.350852706	−0.012944659	−0.380531498
167	STAPHYLOCOCCUS_AUREUS_INFECTION	0.638234419	0.352124096	0.253586126	0.370657541	0.578711320	0.604810095	0.574709218	0.630834731	0.540220868	0.542791224	0.041459727	0.016747133	0.454217986	0.444722766
168	STARCH_AND_SUCROSE_METABOLISM	0.529749270	0.225719284	0.120806608	0.248919652	0.465473090	0.541903436	0.503504027	0.526127315	0.427180604	0.422960152	0.144576714	−0.104706251	0.344106967	0.319263035
169	STEROID_BIOSYNTHESIS	0.754126037	0.866530630	0.545551887	0.393815431	0.463807585	−0.102051104	0.892665610	0.804122243	0.842061208	0.577361848	−0.038182162	0.941611792	0.920048955	0.555040770
170	STEROID_HORMONE_BIOSYNTHESIS	0.322153663	−0.002448229	−0.086147979	0.063113180	0.277285179	0.456668320	0.330621639	0.319228055	0.210469906	0.219216949	0.266949901	−0.322110072	0.127439268	0.113897034
171	SULFUR_METABOLISM	0.805268751	0.579356167	0.395597765	0.456754673	0.665893268	0.521089329	0.787096571	0.813146384	0.742381705	0.666980090	0.022699490	0.296919481	0.687197693	0.571601570
172	SYSTEMIC_LUPUS_ERYTHEMATOSUS	0.093831581	0.411436599	0.303294962	0.104465867	−0.028700602	−0.457683887	0.179242232	0.125019363	0.230494908	0.077057219	−0.190552739	0.715514896	0.341867776	0.148406205
173	T_CELL_RECEPTOR_SIGNALING_PATHWAY	0.511208848	0.229484658	0.009075718	0.099762957	0.342363280	0.329394688	0.615308007	0.533139001	0.436475188	0.327468413	0.344931995	−0.039636386	0.392650947	0.211729145
174	TASTE_TRANSDUCTION	−0.102125915	0.130166269	−0.216828986	−0.417761484	−0.430623741	−0.855900678	0.299026372	−0.018247587	0.044238574	−0.306342605	0.464407988	0.470648111	0.209753164	−0.296100567

	Pathways	Erismodegib	Vismodegib	Temsirolimus	Ibrutinib	RXDX-101	Ruxolitinib	Alectinib	Vemurafenib	Dabrafenib	Nilotinib	Dasatinib	Bosutinib	ABT-199	Dovitinib
117	NOTCH_SIGNALING_PATHWAY	−0.748246989	−0.795429161	−0.346759687	−0.722376466	−0.531721557	−0.505167188	−0.970979225	−0.632371571	−0.238251559	−0.924351761	−0.953788819	−0.357288448	−0.907232237	−0.782343140
118	NUCLEOTIDE_EXCISION_REPAIR	−0.291795289	0.020276470	0.127312738	−0.364496959	−0.347097380	−0.067516428	0.372216224	−0.063908714	−0.240584586	−0.017941563	0.220321752	−0.339918848	0.087212423	−0.125213431
119	O_GLYCAN_BIOSYNTHESIS	0.257146046	−0.051069905	−0.406279728	0.327425520	0.165070948	−0.150377425	−0.128881011	−0.081943555	−0.094125309	0.122140403	−0.070292901	0.064021402	−0.009921144	0.090870252
120	OLFACTORY_TRANSDUCTION	−0.069020910	−0.344709511	−0.222612186	0.001537493	0.080110176	−0.134576634	−0.731648811	−0.197170023	0.129166786	−0.379610835	−0.591350079	0.155550004	−0.456140083	−0.219726033
121	ONE_CARBON_POOL_BY_FOLATE	−0.031916819	0.284155065	0.367265102	−0.109482479	−0.082359959	0.198210260	0.548633544	0.206816475	0.002797014	0.222254964	0.440783104	−0.084759746	0.332026529	0.140442785
122	OOCYTE_MEIOSIS	−0.458165435	−0.163162464	−0.050180981	−0.524215914	−0.517589439	−0.252400087	0.238983827	−0.250776931	−0.404723269	−0.180253983	0.061646773	−0.506261266	−0.083740748	−0.303243957
123	OTHER_GLYCAN_DEGRADATION	0.728532686	0.472869146	0.216511625	0.779834468	0.713785086	0.460821576	0.138587892	0.502045527	0.508818065	0.525095149	0.306962384	0.644110110	0.431608956	0.598186684
124	OXIDATIVE_PHOSPHORYLATION	0.906227584	0.986369177	0.905298860	0.872809213	0.904419016	0.964300529	0.749206649	0.987798547	0.838270170	0.897806286	0.876467330	0.864846390	0.935393058	0.962488207
125	P53_SIGNALING_PATHWAY	−0.619974025	−0.450702024	−0.572889188	−0.654288972	−0.789608956	−0.668661266	0.131585438	−0.610824687	−0.831388739	−0.312759865	−0.107853453	−0.853359570	−0.286194222	−0.537174060
126	PANCREATIC_CANCER	0.351131046	0.329496042	−0.240385058	0.347058900	0.054979916	−0.050956175	0.733965308	0.107574271	−0.310257297	0.582041000	0.621423241	−0.157554286	0.520373620	0.341373227
127	PANTOTHENATE_AND_COA_BIOSYNTHESIS	0.784910723	0.645125263	0.130097460	0.804535855	0.589640435	0.394512788	0.673908903	0.521413123	0.234652399	0.817242503	0.718341509	0.410140288	0.733702968	0.717214656
128	PARKINSONS_DISEASE	0.846896037	0.971979492	0.869500262	0.802963120	0.814497323	0.908771667	0.831010708	0.943527370	0.741297641	0.899667995	0.920266047	0.762097716	0.947942600	0.926705463
129	PATHOGENIC_ESCHERICHIA_COLI_INFECTION	0.989549155	0.919247865	0.605089391	0.990562400	0.905028477	0.801345263	0.724216965	0.875782120	0.676435947	0.952460949	0.850993293	0.793665741	0.918038311	0.963731974
130	PATHWAYS_IN_CANCER	0.665350203	0.439051663	−0.045762626	0.708439657	0.504690084	0.240409464	0.398512456	0.350858232	0.162676758	0.618028072	0.463931152	0.345864455	0.507330757	0.548917345
131	PENTOSE_AND_GLUCURONATE_INTERCONVERSIONS	0.612588568	0.362525140	−0.098688068	0.662526086	0.468760197	0.187781122	0.296159099	0.289487217	0.140337027	0.538098608	0.368429320	0.321388023	0.421169600	0.482562809
132	PENTOSE_PHOSPHATE_PATHWAY	0.846782660	0.969892529	0.896833434	0.803574119	0.831992505	0.929212403	0.796705353	0.955310395	0.776848317	0.882271002	0.896769050	0.790885046	0.933152164	0.925668285
133	PEROXISOME	0.942064921	0.980442347	0.899079257	0.918532054	0.955527465	0.980178596	0.683225913	0.999188309	0.883917239	0.890062040	0.834929163	0.920281136	0.915048596	0.976059629
134	PHENYLALANINE_METABOLISM	0.630809979	0.632066849	0.101022692	0.617385161	0.371596789	0.292604429	0.895894825	0.439841922	0.025380847	0.823840122	0.845137375	0.171098771	0.780219076	0.637745679
135	PHOSPHATIDYLINOSITOL_SIGNALING_SYSTEM	0.120587955	−0.033424217	0.317181013	0.159973929	0.379439923	0.304660543	−0.610411524	0.187110551	0.577848703	−0.223415579	−0.402859320	0.527867337	−0.231392487	0.040156255
136	PI3KAKT_SIGNALING_PATHWAY	0.653326370	0.384363240	0.019805201	0.708151910	0.574272171	0.292669499	0.180583012	0.364304671	0.302786606	0.504835976	0.301967568	0.465301138	0.393433983	0.514082919
137	PORPHYRIN_AND_CHLOROPHYLL_METABOLISM	0.639954603	0.396949795	−0.067466732	0.687722469	0.494224919	0.218864129	0.329119326	0.321969712	0.163815936	0.569913847	0.403074002	0.344878410	0.455523973	0.514153616
138	PPAR_SIGNALING_PATHWAY	0.649631131	0.383097846	0.000309671	0.703760728	0.559010427	0.276585069	0.202246920	0.353741141	0.276562097	0.513492273	0.315659362	0.442915489	0.400775598	0.511564425
139	PRIMARY_BILE_ACID_BIOSYNTHESIS	0.426646508	0.140227075	−0.282458248	0.488569467	0.301905475	−0.004788376	0.078934818	0.083772790	−0.001420962	0.323887338	0.140713900	0.173023923	0.195274000	0.274331883
140	PRIMARY_IMMUNODEFICIENCY	0.554452421	0.339823552	−0.193882823	0.595822843	0.353466796	0.090816785	0.411420789	0.217486384	−0.014724297	0.559019925	0.432426719	0.175039691	0.444128479	0.442539710
141	PRION_DISEASES	0.298080415	−0.019600191	−0.301550226	0.370712581	0.254394260	−0.057357841	−0.200904798	−0.011309330	0.038589305	0.110106412	−0.104999288	0.181926917	−0.015976389	0.127612871
142	PROGESTERONE_MEDIATED_OOCYTE_MATURATION	−0.674169221	−0.455858669	−0.442171116	−0.718853310	−0.784268939	−0.600745482	0.063355844	−0.576493894	−0.735208733	−0.385357832	−0.161119878	−0.802043168	−0.329340731	−0.564688315
143	PROPANOATE_METABOLISM	−0.110086359	0.017858811	0.580191522	−0.135948035	0.167856625	0.363904183	−0.373864681	0.216258293	0.532998276	−0.271694922	−0.257535695	0.366076167	−0.171232852	−0.038936229
144	PROSTATE_CANCER	0.679997903	0.415300823	0.063119345	0.733481323	0.608849786	0.332941993	0.191423707	0.401219299	0.344775908	0.525269396	0.320801259	0.504071120	0.417211997	0.543527903
145	PROTEASOME	0.623183541	0.828460353	0.678150711	0.562081625	0.521600642	0.675118346	0.921725610	0.733767297	0.432983407	0.806522324	0.915473099	0.442749208	0.870099224	0.743274981
146	PROTEIN_DIGESTION_AND_ABSORPTION	0.454803715	0.203030851	−0.295179860	0.507109881	0.278003636	−0.011053931	0.243375655	0.102502563	−0.069352625	0.420019143	0.271056417	0.116962811	0.294839428	0.321392232
147	PROTEIN_EXPORT	−0.049737238	0.233980205	0.630249863	−0.116532822	0.089427917	0.391655042	0.150902500	0.307094788	0.366042230	0.012226062	0.159309826	0.208445745	0.145722817	0.106362761
148	PROXIMAL_TUBULE_BICARBONATE_RECLAMATION	−0.485231537	−0.277249561	0.272582481	−0.525800820	−0.267593715	−0.007680794	−0.403403465	−0.140272905	0.108072051	−0.514029959	−0.401851840	−0.082420612	−0.397902245	−0.375868172
149	PURINE_METABOLISM	−0.570699454	−0.667734745	−0.966886407	−0.540819798	−0.757974999	−0.890090491	−0.208614338	−0.808959617	−0.938462520	−0.424515573	−0.388979276	−0.859996155	−0.501708309	−0.633361509
150	PYRIMIDINE_METABOLISM	−0.205889962	0.116709984	0.332078548	−0.281576044	−0.195147001	0.110802456	0.330286522	0.083584405	−0.022605887	0.008893279	0.228779177	−0.148332835	0.131334797	−0.031768235
151	PYRUVATE_METABOLISM	−0.389044836	−0.075128769	0.165496871	−0.459777102	−0.375088109	−0.073485314	0.185254001	−0.107237177	−0.182894077	−0.170248484	0.057969411	−0.317735784	−0.052016739	−0.222149631
152	REGULATION_OF_ACTIN_CYTOSKELETON	0.653741433	0.393554094	−0.013261359	0.706044197	0.547608838	0.266808334	0.242242876	0.351482303	0.250797919	0.535557106	0.345720027	0.421784422	0.422321959	0.519068651
153	REGULATION_OF_AUTOPHAGY	0.571106478	0.404977761	−0.162799981	0.599901023	0.336947604	0.110113329	0.555543123	0.251374628	−0.047807660	0.636309303	0.548512076	0.140503633	0.533597294	0.485704983
154	RENAL_CELL_CARCINOMA	0.634447365	0.382211197	−0.062849946	0.684743270	0.502297204	0.222127232	0.287195329	0.318776153	0.183090035	0.546422728	0.370386971	0.361229028	0.431079213	0.503634300
155	RENIN_ANGIOTENSIN_SYSTEM	0.715469950	0.505609377	0.018972347	0.753743849	0.552599823	0.302216666	0.461965971	0.414423804	0.209822634	0.677429423	0.530408536	0.391306995	0.572953646	0.608710300
156	RETINOL_METABOLISM	0.485713665	0.189731858	−0.170421654	0.549672439	0.404575442	0.099905956	0.027734486	0.168996220	0.137489859	0.333025328	0.126972475	0.300235942	0.209593882	0.329357439
157	RIBOFLAVIN_METABOLISM	−0.303809267	−0.572384368	−0.816063446	−0.234575353	−0.386406660	−0.653705105	−0.471594790	−0.608355428	−0.557344180	−0.382994628	−0.507462447	−0.449374909	−0.502945073	−0.455914018
158	RIBOSOME	0.355397878	0.180040492	−0.395195410	0.389485353	0.103200437	−0.133746684	0.424949695	0.010616603	−0.281392835	0.445813691	0.374475585	−0.094952468	0.334364112	0.261835106
159	RIG_I_LIKE_RECEPTOR_SIGNALING_PATHWAY	0.855697762	0.660335608	0.270952883	0.889142616	0.755420835	0.533313567	0.469605598	0.617806130	0.470761591	0.760577051	0.594287003	0.629138236	0.674689320	0.759363142
160	RNA_DEGRADATION	−0.626542082	−0.347383852	−0.123352289	−0.685390453	−0.629818410	−0.361497686	−0.000634536	−0.391931633	−0.445195139	−0.398415319	−0.168524235	−0.574880818	−0.298614325	−0.482091296
161	RNA_POLYMERASE	−0.535254954	−0.393713308	0.187723824	−0.558329848	−0.282974582	−0.074632869	−0.600419033	−0.222642624	0.106755884	−0.635428843	−0.571229849	−0.078781649	−0.538271519	−0.462677905
162	SELENOAMINO_ACID_METABOLISM	0.148782225	0.400866299	0.786027632	0.086961745	0.306349472	0.581078112	0.206809872	0.494739478	0.567506453	0.159735858	0.265522897	0.424451659	0.286427087	0.290414470
163	SMALL_CELL_LUNG_CANCER	0.813380037	0.705556148	0.196513559	0.824693752	0.614038589	0.447068353	0.749386875	0.575255360	0.265316299	0.868297978	0.788655528	0.433367631	0.796320450	0.763554410
164	SNARE_INTERACTIONS_IN_VESICULAR_TRANSPORT	0.464197877	0.215891389	−0.286909200	0.515472097	0.284409155	−0.002383979	0.260420483	0.112762630	−0.065686645	0.433744165	0.287062784	0.121264621	0.309385999	0.332787378
165	SPHINGOLIPID_METABOLISM	0.061719996	−0.243727744	−0.249982741	0.136824077	0.155672679	−0.103681185	−0.589088090	−0.133560670	0.118514961	−0.227118598	−0.453235362	0.185871580	−0.324329656	−0.104015773
166	SPLICEOSOME	−0.492392029	−0.203930648	−0.097780745	−0.556383372	−0.556402040	−0.298003931	0.213589813	−0.294782397	−0.447540989	−0.212224438	0.029516484	−0.546930806	−0.119348003	−0.341564179
167	STAPHYLOCOCCUS_AUREUS_INFECTION	0.742445743	0.511841833	0.085419125	0.785974642	0.622777250	0.364417627	0.372358818	0.456962868	0.314499716	0.649756148	0.475405808	0.486363659	0.545884375	0.625074850
168	STARCH_AND_SUCROSE_METABOLISM	0.643393589	0.392147901	−0.050493307	0.693335320	0.513027668	0.234123096	0.291699490	0.330098564	0.195186474	0.553777898	0.377162822	0.372760524	0.439224209	0.513261513
169	STEROID_BIOSYNTHESIS	0.615569151	0.800848825	0.559249890	0.558833990	0.466676808	0.588890151	0.969927633	0.671811158	0.319541330	0.829389635	0.939193682	0.355577484	0.876319894	0.724166960
170	STEROID_HORMONE_BIOSYNTHESIS	0.454436574	0.171843301	−0.256723628	0.514967372	0.327260040	0.023028634	0.108213341	0.113426009	0.019939714	0.354474886	0.172177209	0.195739979	0.227078819	0.304575819
171	SULFUR_METABOLISM	0.858694164	0.704722633	0.247121607	0.881257400	0.706606881	0.508863197	0.626547284	0.617159371	0.383820904	0.837450768	0.711643320	0.550211306	0.757386163	0.784618783
172	SYSTEMIC_LUPUS_ERYTHEMATOSUS	−0.084751908	0.236661100	0.448600230	−0.161884936	−0.066349105	0.238689261	0.398309024	0.211269859	0.100714127	0.113963423	0.321214066	−0.020765022	0.238429980	0.090439236
173	T_CELL_RECEPTOR_SIGNALING_PATHWAY	0.589517124	0.375547004	−0.151448904	0.630279854	0.394899771	0.134043985	0.425276011	0.258543007	0.030007089	0.586314407	0.455561856	0.218911174	0.473179375	0.478500990
174	TASTE_TRANSDUCTION	−0.287180116	−0.020145191	−0.103736672	−0.350237511	−0.438703620	−0.227161863	0.477449226	−0.173384762	−0.455273070	0.044440124	0.280027289	−0.498525358	0.114252956	−0.146093674

	Pathways	cluster	Afatinib	Erlotinib HCl	Dacomitinib	Gefitinib	Lapatinib	Neratinib	CO-1686	Everolimus	BYL719	Pilaralisib	XL765	Pazopanib HCl	Sunitinib Malate
175	TAURINE_AND_HYPOTAURINE_METABOLISM	C1	−0.492888090	−0.191378701	−0.306544331	0.201645194	0.086259030	−0.516895755	−0.617692527	0.052881840	0.116922259	−0.111892110	0.164569323	−0.180514197	−0.479708503
176	TERPENOID_BACKBONE_BIOSYNTHESIS	C3	−0.053310729	0.227911421	0.064189495	0.589901930	0.514100215	−0.092324439	−0.260666517	0.489574516	0.548309909	0.343812879	0.587014283	0.264659162	−0.073030169
177	TGF_BETA_SIGNALING_PATHWAY	C3	0.118406605	−0.318149547	0.369670083	−0.594497682	−0.214394280	0.308043828	0.559177369	−0.225158638	−0.353309189	−0.164751588	−0.349695041	−0.247058651	−0.046106400
178	THYROID_CANCER	C3	0.271451313	−0.126524798	0.335553672	−0.473085816	−0.198010823	0.395291879	0.595520001	−0.189133371	−0.294392312	−0.076835937	−0.314235049	−0.091824824	0.170993952
179	TIGHT_JUNCTION	C2	0.598643728	0.202154914	0.912803388	0.046038291	0.520868387	0.796621244	0.933599604	0.501723925	0.371180989	0.507683199	0.390072215	0.343050969	0.343023463
180	TOLL_LIKE_RECEPTOR_SIGNALING_PATHWAY	C2	0.697988074	0.366250859	0.590235708	0.009557608	0.219052605	0.753403836	0.834837982	0.244916847	0.164080284	0.384115389	0.126854191	0.398579020	0.619114951
181	TRYPTOPHAN_METABOLISM	C3	0.459665463	0.017198581	0.734000028	−0.223217546	0.228125707	0.657837188	0.849693704	0.215218442	0.078888983	0.257727596	0.087524553	0.130633084	0.236913667
182	TYPE_I_DIABETES_MELLITUS	C2	0.936816585	0.683923341	0.877169068	0.458664822	0.696565950	0.985891403	0.973295451	0.714009383	0.644786241	0.798181243	0.622194860	0.757605948	0.810361296
183	TYPE_II_DIABETES_MELLITUS	C1	−0.757650298	−0.943940785	−0.454338728	−0.997888548	−0.849166288	−0.618638200	−0.364372314	−0.876373573	−0.936152803	−0.898302796	−0.910889147	−0.942399756	−0.801080378
184	TYROSINE_METABOLISM	C3	0.101073627	−0.288037663	0.198588383	−0.617151256	−0.353922847	0.232469630	0.458190568	−0.349274402	−0.452801326	−0.247267919	−0.468115208	−0.261409015	0.010504010
185	UBIQUITIN_MEDIATED_PROTEOLYSIS	C1	−0.622787252	−0.479386882	−0.195141389	−0.102102962	−0.018550500	−0.533440136	−0.512741577	−0.072871629	−0.065447206	−0.267124192	0.004444040	−0.417846002	−0.710987261
186	VALINE_LEUCINE_AND_ISOLEUCINE_BIOSYNTHESIS	C2	0.666868766	0.840926951	0.519053538	0.976493845	0.911218753	0.577320607	0.346166922	0.921337652	0.966368433	0.891698475	0.962356149	0.874057091	0.653723384
187	VALINE_LEUCINE_AND_ISOLEUCINE_DEGRADATION	C3	0.223462383	0.618632692	−0.110680170	0.825345848	0.471119011	0.020158649	−0.267127649	0.493097481	0.613409650	0.467988482	0.596558069	0.558468797	0.373034459
188	VASCULAR_SMOOTH_MUSCLE_CONTRACTION	C3	0.475584713	0.061581305	0.566410794	−0.267922746	0.063493518	0.611661904	0.784193333	0.070132964	−0.044119707	0.171387648	−0.060036980	0.124932456	0.333541551
189	VASOPRESSIN_REGULATED_WATER_REABSORPTION	C3	−0.120355037	−0.215654004	−0.444438443	−0.534620008	−0.699399907	−0.191780151	−0.106876925	−0.659150562	−0.653196766	−0.497221452	−0.708774896	−0.324205816	0.030282130
190	VEGF_SIGNALING_PATHWAY	C2	0.740205296	0.435507991	0.583276016	0.077930427	0.251927217	0.773887855	0.830928762	0.282277312	0.211506337	0.428858602	0.169441598	0.459005526	0.679347643
191	VIBRIO_CHOLERAE_INFECTION	C3	0.290022009	0.262866585	−0.231883723	−0.082479853	−0.331728965	0.141937594	0.110038523	−0.271653759	−0.238406289	−0.073124131	−0.315120479	0.142853103	0.476049057
192	VIRAL_MYOCARDITIS	C2	0.843310513	0.522262694	0.928625041	0.313953086	0.655834340	0.953421528	0.999758430	0.659612194	0.563069668	0.716769551	0.555829135	0.625587128	0.660434060
193	WNT_SIGNALING_PATHWAY	C3	0.391613365	−0.010723451	0.852214745	−0.093739758	0.437435771	0.634642187	0.812181689	0.401892699	0.260542749	0.361235059	0.299139170	0.150211168	0.100159386

	Pathways	Axitinib	Tivozanib	Vandetanib	Regorafenib	Selumetinib	Trametinib	Crizotinib	Cabozantinib	Foretinib	Palbociclib HCl	Ribociclib	Abemaciclib	Veliparib	Olaparib
175	TAURINE_AND_HYPOTAURINE_METABOLISM	−0.488035294	−0.185434285	−0.261412832	−0.430203588	−0.584038975	−0.785921200	−0.276478391	−0.447669829	−0.356306778	−0.505571478	0.158643691	0.196060349	−0.225600102	−0.431171142
176	TERPENOID_BACKBONE_BIOSYNTHESIS	−0.046702537	0.270743493	0.119893947	−0.084832606	−0.207947162	−0.617278197	0.124025583	−0.002580496	0.098482463	−0.098093783	−0.017697915	0.613264305	0.229516218	−0.033058859
177	TGF_BETA_SIGNALING_PATHWAY	0.143517300	−0.170542653	−0.320395790	−0.187109895	0.037988265	0.223746548	0.273131693	0.159900611	0.048689204	−0.009797766	0.497334590	−0.427793757	−0.003121512	−0.122022614
178	THYROID_CANCER	0.283764439	−0.045362613	−0.091832761	0.069168627	0.272124087	0.492142441	0.262427372	0.273950422	0.164575162	0.204531782	0.229572676	−0.374658996	0.072086681	0.104002471
179	TIGHT_JUNCTION	0.636319587	0.455294431	0.090174606	0.090749233	0.331106013	0.081649208	0.861282521	0.688621726	0.622315350	0.373979239	0.421571333	0.302351333	0.639228941	0.262964802
180	TOLL_LIKE_RECEPTOR_SIGNALING_PATHWAY	0.702980214	0.431118979	0.381922723	0.500645740	0.686430203	0.707294060	0.567589498	0.683891247	0.601438237	0.646528569	−0.106761985	0.080964214	0.501981904	0.560049860
181	TRYPTOPHAN_METABOLISM	0.492209048	0.230040210	−0.046042162	0.022083128	0.272150699	0.210239007	0.661745575	0.527049602	0.434313697	0.272544769	0.439305167	0.000118777	0.412366372	0.153501185
182	TYPE_I_DIABETES_MELLITUS	0.948998315	0.808271277	0.623584994	0.634685402	0.802366343	0.521085281	0.890412173	0.955993794	0.916851125	0.829871836	−0.159596611	0.577461214	0.877128672	0.757655497
183	TYPE_II_DIABETES_MELLITUS	−0.744326188	−0.914665240	−0.891870073	−0.775543831	−0.713820330	−0.277542533	−0.559992238	−0.734035806	−0.804512527	−0.784198206	0.634572016	−0.945118563	−0.815837455	−0.825506597
184	TYROSINE_METABOLISM	0.113239243	−0.217956980	−0.241429125	−0.069856471	0.121227932	0.413874547	0.114058259	0.103347437	−0.008780298	0.043605949	0.304779644	−0.526951397	−0.098745881	−0.053221058
185	UBIQUITIN_MEDIATED_PROTEOLYSIS	−0.599587834	−0.375095772	−0.579960618	−0.732338875	−0.804034564	−0.968635193	−0.215411175	−0.531750937	−0.474644687	−0.724025214	0.548752338	−0.001704469	−0.323134532	−0.691552484
186	VALINE_LEUCINE_AND_ISOLEUCINE_BIOSYNTHESIS	0.664630008	0.872246718	0.747370007	0.590038040	0.539845346	0.027100488	0.612652290	0.679330344	0.756694375	0.637299474	−0.432722242	0.983673033	0.811015055	0.673111837
187	VALINE_LEUCINE_AND_ISOLEUCINE_DEGRADATION	0.197541100	0.486086164	0.609001188	0.474915997	0.283260122	−0.004977508	0.003466266	0.176272355	0.283685797	0.339751822	−0.652215884	0.669860083	0.317070594	0.439302252
188	VASCULAR_SMOOTH_MUSCLE_CONTRACTION	0.493467490	0.188208533	0.058084964	0.182152030	0.406701170	0.480562690	0.504705906	0.496120873	0.395014414	0.367690674	0.216286230	−0.128681868	0.320640864	0.259777042
189	VASOPRESSIN_REGULATED_WATER_REABSORPTION	−0.147646666	−0.388726187	−0.049536788	0.160736508	0.173246010	0.684922933	−0.471088079	−0.223130773	−0.293413135	0.040836945	−0.272698909	−0.690956760	−0.445252803	0.027813866
190	VEGF_SIGNALING_PATHWAY	0.741750045	0.482922118	0.456248277	0.571949924	0.744312166	0.752962554	0.570878417	0.717293785	0.640960549	0.704551092	−0.187306591	0.130269438	0.536628725	0.625155843
191	VIBRIO_CHOLERAE_INFECTION	0.253050578	0.058424309	0.421861441	0.602214670	0.593802380	0.940547470	−0.208458790	0.162773546	0.116558697	0.480512592	−0.634688075	−0.280021871	−0.051670225	0.482868578
192	VIRAL_MYOCARDITIS	0.866076517	0.702746215	0.439155697	0.446994307	0.650826346	0.370946318	0.916710967	0.891188159	0.839425479	0.685354696	0.063336205	0.492136823	0.820119878	0.594580156
193	WNT_SIGNALING_PATHWAY	0.437716330	0.280133780	−0.144076861	−0.164394053	0.076902456	−0.168614132	0.781404358	0.508861959	0.444458819	0.131429438	0.639365416	0.202323524	0.495250982	0.018315801

	Pathways	Erismodegib	Vismodegib	Temsirolimus	Ibrutinib	RXDX-101	Ruxolitinib	Alectinib	Vemurafenib	Dabrafenib	Nilotinib	Dasatinib	Bosutinib	ABT-199	Dovitinib
175	TAURINE_AND_HYPOTAURINE_METABOLISM	−0.640868358	−0.362814828	−0.094014475	−0.698886565	−0.619011290	−0.344674273	−0.056746439	−0.387964613	−0.406339238	−0.435320284	−0.211899173	−0.547085330	−0.331269357	−0.496990588
176	TERPENOID_BACKBONE_BIOSYNTHESIS	−0.231636431	0.089966332	0.271859597	−0.306973175	−0.241926300	0.057790067	0.348334956	0.040320287	−0.089575953	0.003383949	0.230498433	−0.207600901	0.121225276	−0.058602691
177	TGF_BETA_SIGNALING_PATHWAY	0.259978032	−0.010435745	−0.477483514	0.319928109	0.091939948	−0.209031213	0.065311182	−0.104618517	−0.232818737	0.218472964	0.070890294	−0.055269616	0.086273441	0.113773427
178	THYROID_CANCER	0.427032329	0.133093631	−0.262405812	0.491035356	0.320331372	0.011538174	0.035765674	0.091514255	0.033086342	0.302946341	0.109968441	0.202491802	0.174926635	0.270764808
179	TIGHT_JUNCTION	0.632274056	0.540335917	−0.029732272	0.641718040	0.378927262	0.214887418	0.744627073	0.363831043	−0.001195547	0.759093621	0.719077284	0.173672405	0.681323501	0.588198975
180	TOLL_LIKE_RECEPTOR_SIGNALING_PATHWAY	0.809675707	0.588117624	0.217599641	0.850441012	0.724746274	0.483173410	0.370823859	0.559977760	0.450306735	0.686687226	0.503135780	0.608388869	0.593421242	0.698494022
181	TRYPTOPHAN_METABOLISM	0.549432894	0.361370687	−0.196694977	0.584070128	0.325897612	0.081898308	0.488392010	0.218322712	−0.054524856	0.592166581	0.489170109	0.135463533	0.482993942	0.451809715
182	TYPE_I_DIABETES_MELLITUS	0.958230758	0.891340112	0.508348460	0.958296849	0.830458592	0.718696736	0.784536222	0.813544689	0.560338627	0.964831944	0.879976644	0.692821744	0.923198130	0.933464458
183	TYPE_II_DIABETES_MELLITUS	−0.642357504	−0.838713353	−0.935425862	−0.585148603	−0.687839490	−0.872673246	−0.660577563	−0.859231531	−0.751078881	−0.684893159	−0.746109513	−0.702974074	−0.773271033	−0.759152773
184	TYROSINE_METABOLISM	0.265859377	−0.040557106	−0.404337244	0.335500121	0.168947732	−0.146276275	−0.111565936	−0.075271929	−0.095612638	0.135823883	−0.054830148	0.064473394	0.003595126	0.100644848
185	UBIQUITIN_MEDIATED_PROTEOLYSIS	−0.740308310	−0.522307373	−0.444870026	−0.783477054	−0.818507580	−0.625585780	−0.039221301	−0.619110046	−0.728157309	−0.476143948	−0.255552699	−0.812201006	−0.414720909	−0.631609287
186	VALINE_LEUCINE_AND_ISOLEUCINE_BIOSYNTHESIS	0.524206320	0.767534553	0.811937108	0.456567199	0.516099695	0.731888836	0.745751557	0.739312763	0.554889487	0.654208235	0.769733746	0.506582876	0.749168278	0.664653548
187	VALINE_LEUCINE_AND_ISOLEUCINE_DEGRADATION	0.080809627	0.345229483	0.736812150	0.016931787	0.233132963	0.518962199	0.188083711	0.432633270	0.501362850	0.109249451	0.229847336	0.352374199	0.239041793	0.228229900
188	VASCULAR_SMOOTH_MUSCLE_CONTRACTION	0.602983794	0.352730118	−0.112148690	0.653075827	0.456356961	0.174591279	0.294218083	0.277416289	0.125895223	0.531573883	0.363327814	0.307629533	0.413959239	0.472697523
189	VASOPRESSIN_REGULATED_WATER_REABSORPTION	0.044928881	−0.239791653	−0.154395347	0.115832935	0.184308318	−0.043193462	−0.649645195	−0.097159841	0.206619196	−0.270525521	−0.494542637	0.246826664	−0.351376749	−0.109262684
190	VEGF_SIGNALING_PATHWAY	0.846243332	0.635402846	0.296317691	0.884070535	0.779040000	0.551668701	0.380887577	0.620207498	0.524242154	0.711618157	0.527090245	0.673359166	0.626092735	0.741595265
191	VIBRIO_CHOLERAE_INFECTION	0.420466148	0.193139817	0.319217335	0.472086849	0.594708854	0.424161953	−0.365508130	0.361045541	0.638205108	0.084925475	−0.142277166	0.666225611	0.035589428	0.303167563
192	VIRAL_MYOCARDITIS	0.870838424	0.790520958	0.316831280	0.874770248	0.687988421	0.550163136	0.801582686	0.670485079	0.362522626	0.924753961	0.853523718	0.517668196	0.866812242	0.837022508
193	WNT_SIGNALING_PATHWAY	0.413540163	0.344166535	−0.243335871	0.420327941	0.126488992	−0.022318535	0.688128346	0.135862259	−0.256289454	0.600753565	0.602078616	−0.087622800	0.523271331	0.378547744
