# Supplementary material for: Patient-Derived Tumor Organoids for Guidance of Personalized Drug Therapies in Recurrent Glioblastoma
Source: Int J Mol Sci. 2022 Jun 12;23(12):6572. doi: 10.3390/ijms23126572 (PMC9223608; doi:10.3390/ijms23126572)
Supplement: Supplementary file 1 [file ijms-23-06572-s001.zip › TableS1.pdf]

Table S1. Mean area-under-the-curve values for all tested drugs in the individual patients MA01-MA04 and their corresponding mean z-score. The color-coding of the drugs is analogous to the colors used to group drugs in Figure 4. Abbreviation: SD, standard deviation

	MA01				MA02				MA03				MA04			
	mean AUC	SD AUC	z-score	SD z-score	mean AUC	SD AUC	z-score	SD z-score	mean AUC	SD AUC	z-score	SD z-score	mean AUC	SD AUC	z-score	SD z-score
Afatinib	236.50	8.75	−0.94	0.21	251.34	11.55	−0.90	0.22	269.39	0.00	−1.52	0.00	247.87	10.61	−0.72	0.20
Erlotinib HCl	317.44	19.03	1.03	0.46	340.57	18.43	0.83	0.36	402.13	0.00	0.40	0.00	313.45	5.19	0.53	0.10
Dacomitinib	266.66	10.57	−0.20	0.26	295.59	25.40	−0.04	0.49	363.81	0.00	−0.16	0.00	326.53	3.70	0.77	0.07
Gefitinib	298.58	11.91	0.57	0.29	329.50	20.42	0.62	0.40	377.46	0.00	0.04	0.00	300.50	6.74	0.28	0.13
Lapatinib	297.68	23.25	0.55	0.57	341.94	20.23	0.86	0.39	403.91	0.00	0.42	0.00	328.40	9.29	0.81	0.18
Neratinib	246.04	29.20	−0.71	0.71	264.88	11.62	−0.63	0.23	358.82	0.00	−0.23	0.00	281.11	4.56	−0.09	0.09
CO-1686	259.14	20.76	−0.39	0.50	265.69	13.86	−0.62	0.27	404.44	0.00	0.43	0.00	309.10	8.54	0.44	0.16
Everolimus	198.39	29.86	−1.86	0.73	306.76	26.45	0.18	0.51	392.42	0.00	0.26	0.00	262.85	9.62	−0.44	0.18
BYL719	242.87	28.11	−0.78	0.68	319.46	28.02	0.42	0.54	396.18	0.00	0.31	0.00	277.00	8.61	−0.17	0.16
Pilaralisib	266.05	30.62	−0.22	0.74	333.54	22.54	0.70	0.44	444.82	0.00	1.01	0.00	303.60	9.59	0.34	0.18
XL765	239.24	32.01	−0.87	0.78	316.76	17.44	0.37	0.34	366.60	0.00	−0.12	0.00	278.20	9.80	−0.14	0.19
Pazopanib HCl	323.66	15.43	1.18	0.37	344.22	19.80	0.90	0.38	422.07	0.00	0.68	0.00	327.75	11.57	0.80	0.22
Sunitinib Malate	271.55	22.58	−0.09	0.55	283.14	18.90	−0.28	0.37	299.68	0.00	−1.08	0.00	263.00	9.48	−0.43	0.18
Axitinib	281.06	19.45	0.15	0.47	302.67	20.37	0.10	0.39	405.54	0.00	0.45	0.00	301.07	7.19	0.29	0.14
Tivozanib	300.44	33.49	0.62	0.81	336.15	14.43	0.75	0.28	411.60	0.00	0.53	0.00	315.78	11.12	0.57	0.21
Vandetanib	265.97	18.43	−0.22	0.45	286.17	20.70	−0.22	0.40	293.45	0.00	−1.17	0.00	248.72	11.95	−0.70	0.23
Regorafenib	286.45	30.31	0.28	0.74	293.00	16.50	−0.09	0.32	351.20	0.00	−0.34	0.00	251.58	9.28	−0.65	0.18
Selumetinib	312.24	16.07	0.90	0.39	312.73	14.12	0.29	0.27	368.50	0.00	−0.09	0.00	299.57	5.73	0.26	0.11
Trametinib	315.14	36.23	0.97	0.88	263.47	10.45	−0.66	0.20	348.18	0.00	−0.38	0.00	257.30	10.74	−0.54	0.20
Crizotinib	144.80	15.57	−3.17	0.38	210.33	8.54	−1.69	0.17	357.23	0.00	−0.25	0.00	266.48	10.46	−0.37	0.20
Cabozantinib	289.08	31.68	0.34	0.77	314.97	19.27	0.34	0.37	445.26	0.00	1.02	0.00	317.09	12.44	0.59	0.24
Foretinib	221.30	28.18	−1.31	0.68	254.15	21.66	−0.84	0.42	359.50	0.00	−0.22	0.00	246.98	14.55	−0.74	0.28
Palbociclib HCl	308.28	20.56	0.81	0.50	321.24	16.25	0.46	0.31	384.65	0.00	0.14	0.00	299.20	10.58	0.25	0.20
Ribociclib	354.52	8.40	1.93	0.20	354.80	15.12	1.11	0.29	366.04	0.00	−0.12	0.00	376.47	8.42	1.72	0.16
Abemaciclib	258.22	35.16	−0.41	0.85	318.66	13.25	0.41	0.26	304.97	0.00	−1.01	0.00	282.83	12.50	−0.06	0.24
Veliparib	269.58	46.00	−0.13	1.12	322.62	13.67	0.48	0.26	430.52	0.00	0.81	0.00	313.02	6.51	0.52	0.12
Olaparib	325.23	14.37	1.22	0.35	338.64	12.04	0.79	0.23	420.74	0.00	0.67	0.00	311.94	8.17	0.50	0.16
Erismodegib	279.50	27.41	0.11	0.67	286.43	19.77	−0.22	0.38	437.26	0.00	0.90	0.00	293.60	15.05	0.15	0.29
Vismodegib	297.19	31.69	0.54	0.77	328.28	12.73	0.59	0.25	438.32	0.00	0.92	0.00	314.07	15.54	0.54	0.30
Temsirolimus	286.89	15.67	0.29	0.38	308.91	10.83	0.22	0.21	286.38	0.00	−1.28	0.00	272.75	10.04	−0.25	0.19
Ibrutinib	273.96	27.27	−0.03	0.66	272.14	13.01	−0.49	0.25	476.38	0.00	1.47	0.00	286.06	14.59	0.01	0.28
RXDX-101	250.38	6.64	−0.60	0.16	249.40	12.65	−0.93	0.24	255.76	0.00	−1.72	0.00	239.35	5.77	−0.88	0.11
Ruxolitinib	316.13	20.99	1.00	0.51	340.12	17.25	0.82	0.33	371.40	0.00	−0.05	0.00	300.77	7.48	0.28	0.14
Ceritinib/Alectinib	255.53	12.91	−0.47	0.31	341.00	21.89	0.84	0.42	448.39	0.00	1.06	0.00	354.84	10.87	1.31	0.21
Vemurafenib	292.84	32.79	0.43	0.80	310.96	25.14	0.26	0.49	436.02	0.00	0.89	0.00	291.05	11.35	0.10	0.22
Dabrafenib	333.66	37.46	1.42	0.91	336.52	14.35	0.75	0.28	441.21	0.00	0.96	0.00	291.72	6.21	0.11	0.12
Nilotinib	329.77	7.59	1.33	0.18	350.83	12.96	1.03	0.25	444.30	0.00	1.01	0.00	358.15	16.55	1.37	0.31
Dasatinib	198.55	40.45	−1.86	0.98	267.25	10.37	−0.59	0.20	374.29	0.00	−0.01	0.00	270.17	6.24	−0.30	0.12
Bosutinib	269.74	24.13	−0.13	0.59	266.60	12.51	−0.60	0.24	292.55	0.00	−1.19	0.00	243.01	5.86	−0.81	0.11
ABT-199	245.37	46.77	−0.72	1.14	295.34	18.54	−0.04	0.36	429.02	0.00	0.78	0.00	292.99	7.52	0.14	0.14
Dovitinib	252.19	22.11	−0.56	0.54	273.20	17.85	−0.47	0.35	353.35	0.00	−0.31	0.00	267.30	7.73	−0.35	0.15
Bortezomib	52.62	6.32	−5.41	0.15	49.74	2.71	−4.80	0.05	102.53	0.00	−3.93	0.00	20.09	1.17	−5.05	0.02
