# Supplementary material for: Patient-Derived Tumor Organoids for Guidance of Personalized Drug Therapies in Recurrent Glioblastoma
Source: Int J Mol Sci. 2022 Jun 12;23(12):6572. doi: 10.3390/ijms23126572 (PMC9223608; doi:10.3390/ijms23126572)
Supplement: Supplementary file 1 [file ijms-23-06572-s001.zip › TableS3.pdf]

Table S3. Pearson correlation coefficients of PD-GBO drug response score and specific inter-individual KEGG pathway enrichment levels from RNA-seq of glioblastoma tumor tissue.

| | Patient ID | | | |
|---|---|---|---|---|
| | MA01 | MA02 | MA03 | MA04 |
| N_GLYCAN_BIOSYNTHESIS | -0.575057848 | 1.439325655 | -0.097342393 | -0.766925414 |
| OTHER_GLYCAN_DEGRADATION | 0.108933002 | -0.261672798 | 1.282188339 | -1.129448543 |
| O_GLYCAN_BIOSYNTHESIS | -0.6064274 | -0.506166616 | 1.493609241 | -0.381015225 |
| GLYCOSAMINOGLYCAN_DEGRADATION | -0.28818805 | -0.126198375 | 1.39360873 | -0.979222306 |
| GLYCOSAMINOGLYCAN_BIOSYNTHESIS_KERATAN_SULFATE | 0.163788922 | -0.032738805 | 1.151749416 | -1.282799533 |
| GLYCEROLIPID_METABOLISM | 0.484891581 | 0.318766881 | 0.67991343 | -1.483571892 |
| GLYCOSYLPHOSPHATIDYLINOSITOL_GPI_ANCHOR_BIOSYNTHESIS | -0.807092358 | -0.904789107 | 1.035787229 | 0.676094235 |
| GLYCEROPHOSPHOLIPID_METABOLISM | 0.458856872 | 0.846101862 | 0.12921858 | -1.434177314 |
| ETHER_LIPID_METABOLISM | -0.694294278 | -0.148602199 | 1.456009886 | -0.613113409 |
| ARACHIDONIC_ACID_METABOLISM | -0.550610942 | 0.058327646 | 1.380217054 | -0.887933758 |
| LINOLEIC_ACID_METABOLISM | 1.29336618 | -0.517374964 | 0.227815194 | -1.00380641 |
| ALPHA_LINOLENIC_ACID_METABOLISM | -0.331681031 | 1.487682653 | -0.643644329 | -0.512357293 |
| SPHINGOLIPID_METABOLISM | 0.161265938 | -1.193721475 | 1.228983028 | -0.196527491 |
| GLYCOSPHINGOLIPID_BIOSYNTHESIS_LACTO_AND_NEOLACTO_SERIES | -0.245763043 | -1.292928521 | 0.564223256 | 0.974468308 |
| GLYCOSPHINGOLIPID_BIOSYNTHESIS_GLOBO_SERIES | -0.979853933 | 0.639918677 | 1.056706701 | -0.716771446 |
| GLYCOSPHINGOLIPID_BIOSYNTHESIS_GANGLIO_SERIES | -0.046390879 | -0.224392121 | 1.341798219 | -1.07101522 |
| RIBOFLAVIN_METABOLISM | -0.866089182 | -0.787779502 | 1.188633232 | 0.465235452 |
| NICOTINATE_AND_NICOTINAMIDE_METABOLISM | -0.453035563 | 1.148670766 | 0.43747218 | -1.133107383 |
| PANTOTHENATE_AND_COA_BIOSYNTHESIS | -0.592613981 | 0.684824599 | 0.996864619 | -1.089075236 |
| AMINOACYL_TRNA_BIOSYNTHESIS | 0.934070134 | 0.510744386 | -1.363854319 | -0.080960201 |
| BASAL_TRANSCRIPTION_FACTORS | -0.006598709 | 1.000850067 | -1.364301474 | 0.370050116 |
| NON_HOMOLOGOUS_END_JOINING | 0.120717757 | 0.735760943 | -1.447382416 | 0.590903716 |
| SNARE_INTERACTIONS_IN_VESICULAR_TRANSPORT | -0.832386664 | 0.085379249 | 1.378698412 | -0.631690996 |
| LYSOSOME | -0.006928037 | -0.003313716 | 1.229843003 | -1.21960125 |
| CARDIAC_MUSCLE_CONTRACTION | 0.630025264 | 0.021489719 | -1.419002769 | 0.767487786 |
| RENIN_ANGIOTENSIN_SYSTEM | -0.555677024 | 0.321968448 | 1.248280272 | -1.014571696 |
| CIRCADIAN_RHYTHM_MAMMAL | 0.057360381 | 0.476155473 | 0.879467655 | -1.412983509 |
| TASTE_TRANSDUCTION | -0.615506676 | 1.098039899 | -1.047194883 | 0.56466166 |
| PROXIMAL_TUBULE_BICARBONATE_RECLAMATION | 0.956767941 | -0.339838488 | -1.25154583 | 0.634616376 |
| PATHOGENIC_ESCHERICHIA_COLI_INFECTION | 0.134886696 | 0.701596219 | 0.616098064 | -1.452580979 |
| SYSTEMIC_LUPUS_ERYTHEMATOSUS | 0.376308432 | 0.886586153 | -1.429853702 | 0.166959118 |
| PRIMARY_IMMUNODEFICIENCY | -0.841748028 | 0.314989765 | 1.276666991 | -0.749908728 |
| HYPERTROPHIC_CARDIOMYOPATHY_HCM | -0.242761243 | 0.362857251 | 1.123630598 | -1.243726607 |
| ARRHYTHMOGENIC_RIGHT_VENTRICULAR_CARDIOMYOPATHY_ARVC | 0.053451961 | 0.109793727 | 1.13734468 | -1.300590368 |

| | | | | |
|---|---|---|---|---|
| DILATED_CARDIOMYOPATHY | -0.433575213 | 0.325484343 | 1.215990068 | -1.107899198 |
| GLYCOLYSIS_GLUCONEOGENESIS | 0.966052474 | -0.194436708 | 0.544579546 | -1.316195313 |
| CITRATE_CYCLE_TCA_CYCLE | 0.477538893 | 1.128870435 | -1.125177243 | -0.481232084 |
| PENTOSE_PHOSPHATE_PATHWAY | 0.583826661 | 0.995383912 | -0.330582258 | -1.248628315 |
| PENTOSE_AND_GLUCURONATE_INTERCONVERSIONS | -0.571307765 | 0.070152349 | 1.378209676 | -0.87705426 |
| FRUCTOSE_AND_MANNOSE_METABOLISM | 1.370911296 | -0.933916783 | 0.05801636 | -0.495010873 |
| GALACTOSE_METABOLISM | 0.717505951 | 0.576955891 | 0.163475986 | -1.457937828 |
| ASCORBATE_AND_ALDARATE_METABOLISM | -0.667337578 | 0.174344137 | 1.342567752 | -0.849574311 |
| FATTY_ACID_METABOLISM | 0.441271629 | -0.938665897 | 1.197508273 | -0.700114005 |
| STEROID_BIOSYNTHESIS | -0.131556609 | 1.441068575 | -0.498865915 | -0.810646051 |
| PRIMARY_BILE_ACID_BIOSYNTHESIS | -0.633997106 | -0.225526325 | 1.473264299 | -0.613740868 |
| STEROID_HORMONE_BIOSYNTHESIS | -0.624347641 | -0.187600721 | 1.465541527 | -0.653593165 |
| OXIDATIVE_PHOSPHORYLATION | 0.636781135 | 0.862204997 | -0.146321193 | -1.352664939 |
| PURINE_METABOLISM | -1.347387155 | -0.115357586 | 0.506173134 | 0.956571607 |
| PYRIMIDINE_METABOLISM | 0.266200994 | 0.832335118 | -1.452862126 | 0.354326015 |
| ALANINE_ASPARTATE_AND_GLUTAMATE_METABOLISM | -0.205619403 | 1.047658133 | -1.28866517 | 0.44662644 |
| GLYCINE_SERINE_AND_THREONINE_METABOLISM | 0.483176388 | -0.223863684 | 1.028532771 | -1.287845475 |
| CYSTEINE_AND_METHIONINE_METABOLISM | 0.741076605 | 0.483461064 | -1.468809731 | 0.244272062 |
| VALINE_LEUCINE_AND_ISOLEUCINE_DEGRADATION | 1.05572676 | 0.413534682 | -1.298131157 | -0.171130286 |
| VALINE_LEUCINE_AND_ISOLEUCINE_BIOSYNTHESIS | 0.543772034 | 1.132595587 | -0.928834267 | -0.747533354 |
| LYSINE_DEGRADATION | 0.210351392 | 0.740966147 | -1.464147245 | 0.512829705 |
| ARGININE_AND_PROLINE_METABOLISM | -0.199938433 | 0.633549915 | 0.893291027 | -1.326902509 |
| HISTIDINE_METABOLISM | -1.127104345 | -0.079973861 | 1.308777034 | -0.101698829 |
| TYROSINE_METABOLISM | -0.622154593 | -0.480037101 | 1.493187875 | -0.390996181 |
| PHENYLALANINE_METABOLISM | -0.842616366 | 1.214288529 | 0.425077549 | -0.796749713 |
| TRYPTOPHAN_METABOLISM | -0.924005774 | 0.462728739 | 1.186072239 | -0.724795204 |
| BETA_ALANINE_METABOLISM | -0.49448818 | -0.583550782 | -0.418546685 | 1.496585647 |
| TAURINE_AND_HYPOTAURINE_METABOLISM | 0.002878292 | 0.36845834 | -1.367887568 | 0.996550936 |
| SELENOAMINO_ACID_METABOLISM | 1.109627873 | 0.404819687 | -1.235800471 | -0.27864709 |
| GLUTATHIONE_METABOLISM | -0.528627157 | 1.273720984 | 0.268001984 | -1.01309581 |
| STARCH_AND_SUCROSE_METABOLISM | -0.489426631 | 0.046229936 | 1.37487904 | -0.931682345 |
| AMINO_SUGAR_AND_NUCLEOTIDE_SUGAR_METABOLISM | 0.619786023 | -0.122246606 | 0.864142758 | -1.361682175 |
| GLYCOSAMINOGLYCAN_BIOSYNTHESIS_CHONDROITIN_SULFATE | 0.313262933 | 0.620719043 | 0.552914164 | -1.48689614 |
| GLYCOSAMINOGLYCAN_BIOSYNTHESIS_HEPARAN_SULFATE | 1.008447893 | -1.161324892 | 0.634413255 | -0.481536255 |
| INOSITOL_PHOSPHATE_METABOLISM | 0.654871142 | -1.489399355 | 0.369229328 | 0.465298884 |
| PYRUVATE_METABOLISM | 0.159035873 | 0.672784369 | -1.45900799 | 0.627187748 |
| GLYOXYLATE_AND_DICARBOXYLATE_METABOLISM | 1.4114758 | -0.532858741 | -0.850291291 | -0.028325768 |

| | | | | |
|---|---|---|---|---|
| PROPANOATE_METABOLISM | 1.390872252 | -0.520387983 | -0.891202273 | 0.020718004 |
| BUTANOATE_METABOLISM | 0.86467464 | -0.335944815 | -1.264290218 | 0.735560394 |
| ONE_CARBON_POOL_BY_FOLATE | 0.075565674 | 1.1145528 | -1.317510237 | 0.127391763 |
| FOLATE_BIOSYNTHESIS | 0.659596739 | -1.450043457 | 0.668996173 | 0.121450545 |
| RETINOL_METABOLISM | -0.40005026 | -0.348121883 | 1.478370946 | -0.730198802 |
| PORPHYRIN_AND_CHLOROPHYLL_METABOLISM | -0.555946963 | 0.11622094 | 1.355806712 | -0.916080688 |
| TERPENOID_BACKBONE_BIOSYNTHESIS | 0.149095649 | 0.875871251 | -1.430454437 | 0.405487537 |
| LIMONENE_AND_PINENE_DEGRADATION | -1.34378239 | 0.96074394 | -0.123012762 | 0.506051212 |
| NITROGEN_METABOLISM | 1.115410275 | -0.91094323 | -0.774966368 | 0.570499323 |
| SULFUR_METABOLISM | -0.356473229 | 0.558994659 | 1.025669705 | -1.228191135 |
| METABOLISM_OF_XENOBIOTICS_BY_CYTOCHROME_P450 | -0.551637168 | -0.080324149 | 1.431677058 | -0.799715741 |
| DRUG_METABOLISM_CYTOCHROME_P450 | -0.401264737 | -0.101007491 | 1.413600178 | -0.91132795 |
| DRUG_METABOLISM_OTHER_ENZYMES | -0.418610854 | -0.149598056 | 1.433224704 | -0.865015793 |
| BIOSYNTHESIS_OF_UNSATURATED_FATTY_ACIDS | 0.886755378 | 0.577019145 | -1.368045999 | -0.095728523 |
| ABC_TRANSPORTERS | 1.418526216 | -0.930258063 | -0.204131473 | -0.28413668 |
| RIBOSOME | -1.166945585 | 0.45922198 | 1.120972233 | -0.413248627 |
| RNA_DEGRADATION | -0.106739471 | 0.4578519 | -1.341175868 | 0.990063439 |
| RNA_POLYMERASE | 1.020243495 | -0.685188447 | -1.014135184 | 0.679080136 |
| DNA_REPLICATION | -0.10409618 | 0.786681442 | -1.375056379 | 0.692471117 |
| SPLICEOSOME | -0.303043324 | 0.761218626 | -1.283542165 | 0.825366863 |
| PROTEASOME | 0.119592033 | 1.364622169 | -0.637087618 | -0.847126583 |
| PROTEIN_EXPORT | 0.932846871 | 0.425987883 | -1.395348622 | 0.036513868 |
| PPAR_SIGNALING_PATHWAY | -0.312585618 | -0.117864093 | 1.397543512 | -0.967093801 |
| BASE_EXCISION_REPAIR | -0.038516298 | 0.937460876 | -1.375656105 | 0.476711527 |
| NUCLEOTIDE_EXCISION_REPAIR | -0.112498953 | 0.942653075 | -1.351605553 | 0.521451431 |
| MISMATCH_REPAIR | 0.123222837 | 0.607722776 | -1.449065999 | 0.718120386 |
| HOMOLOGOUS_RECOMBINATION | 0.203186025 | 0.640092258 | -1.468882716 | 0.625604433 |
| MAPK_SIGNALING_PATHWAY | -0.481881891 | 0.164777578 | 1.318371241 | -1.001266929 |
| ERBB_SIGNALING_PATHWAY | 0.280191199 | -1.478079771 | 0.697190323 | 0.500698248 |
| CALCIUM_SIGNALING_PATHWAY | 0.371691361 | -1.35779269 | 1.008825144 | -0.022723815 |
| CYTOKINE_CYTOKINE_RECEPTOR_INTERACTION | -0.566683501 | 0.180792644 | 1.326904853 | -0.941013996 |
| CHEMOKINE_SIGNALING_PATHWAY | -0.615449076 | 0.035672004 | 1.397110108 | -0.817333035 |
| PHOSPHATIDYLINOSITOL_SIGNALING_SYSTEM | 1.14338741 | -1.184383654 | 0.400669297 | -0.359673053 |
| NEUROACTIVE_LIGAND_RECEPTOR_INTERACTION | -0.110064399 | -1.360840688 | 0.570805098 | 0.900099989 |
| CELL_CYCLE | -0.171595535 | 0.596380118 | -1.335927951 | 0.911143368 |
| OOCYTE_MEIOSIS | -0.253106099 | 0.790570795 | -1.309525548 | 0.772060852 |
| P53_SIGNALING_PATHWAY | -1.011252676 | 0.62599804 | -0.676649368 | 1.061904004 |
| UBIQUITIN_MEDIATED_PROTEOLYSIS | -0.598660893 | 0.419050534 | -1.016963054 | 1.196573413 |
| REGULATION_OF_AUTOPHAGY | -0.937901217 | 0.577726841 | 1.107909966 | -0.74773559 |
| ENDOCYTOSIS | -0.001527888 | -0.813599092 | 1.409021973 | -0.593894993 |
| PEROXISOME | 0.687315796 | 0.709987612 | 0.024980171 | -1.422283579 |
| MTOR_SIGNALING_PATHWAY | -1.064694913 | 0.508149747 | 1.130721208 | -0.574176042 |

| | | | | |
|---|---|---|---|---|
| APOPTOSIS | -0.403177734 | 0.045145891 | 1.35615571 | -0.998123867 |
| VASCULAR_SMOOTH_MUSCLE_CONTRACTION | -0.59077067 | 0.071623384 | 1.380087622 | -0.860940336 |
| WNT_SIGNALING_PATHWAY | -1.18444026 | 0.933601696 | 0.714498713 | -0.463660148 |
| DORSO_VENTRAL_AXIS_FORMATION | 0.083889114 | -1.113215538 | 1.296953868 | -0.267627444 |
| NOTCH_SIGNALING_PATHWAY | 0.518047112 | -1.306122224 | -0.203934323 | 0.992009435 |
| HEDGEHOG_SIGNALING_PATHWAY | -0.030239825 | 0.218088318 | -1.30509713 | 1.117248638 |
| TGF_BETA_SIGNALING_PATHWAY | -0.926182445 | -0.155963339 | 1.41638477 | -0.334238986 |
| AXON_GUIDANCE | 0.221411079 | -0.98895265 | 1.29984659 | -0.532305019 |
| VEGF_SIGNALING_PATHWAY | -0.020432105 | 0.11596032 | 1.173213557 | -1.268741772 |
| FOCAL_ADHESION | -0.230878637 | 0.304160673 | 1.157405927 | -1.230687963 |
| ECM_RECEPTOR_INTERACTION | -0.276988094 | 0.648421501 | 0.917425573 | -1.28885898 |
| CELL_ADHESION_MOLECULES_CAMS | -0.144801638 | -0.425297397 | 1.433014708 | -0.862915672 |
| ADHERENS_JUNCTION | -0.071077522 | -0.356142002 | 1.391924788 | -0.964705264 |
| TIGHT_JUNCTION | -0.912688742 | 0.907693682 | 0.821972656 | -0.816977596 |
| GAP_JUNCTION | -0.418608482 | -0.91499536 | 1.407848757 | -0.074244914 |
| COMPLEMENT_AND_COAGULATION_CASCADES | -0.2062586 | -0.219217053 | 1.39993758 | -0.974461927 |
| ANTIGEN_PROCESSING_AND_PRESENTATION | -0.124325456 | 0.018893954 | 1.273090345 | -1.167658843 |
| TOLL_LIKE_RECEPTOR_SIGNALING_PATHWAY | -0.138802556 | 0.111974206 | 1.231575853 | -1.204747503 |
| NOD_LIKE_RECEPTOR_SIGNALING_PATHWAY | -0.207314427 | -0.078621504 | 1.349193423 | -1.063257492 |
| RIG_I_LIKE_RECEPTOR_SIGNALING_PATHWAY | -0.155607542 | 0.27089918 | 1.145631756 | -1.260923394 |
| CYTOSOLIC_DNA_SENSING_PATHWAY | 0.135327037 | -0.354264834 | 1.29947229 | -1.080534493 |
| JAK_STAT_SIGNALING_PATHWAY | -0.507669086 | 0.070238079 | 1.367990442 | -0.930559435 |
| HEMATOPOIETIC_CELL_LINEAGE | -0.550919364 | 0.168600777 | 1.330233014 | -0.947914427 |
| NATURAL_KILLER_CELL_MEDIATED_CYTOTOXICITY | -0.382662515 | -0.063914609 | 1.3958432 | -0.949266076 |
| T_CELL_RECEPTOR_SIGNALING_PATHWAY | -0.789051319 | 0.321198883 | 1.27430748 | -0.806455045 |
| B_CELL_RECEPTOR_SIGNALING_PATHWAY | -0.027948041 | -0.217312022 | 1.33133143 | -1.086071368 |
| FC_EPSILON_RI_SIGNALING_PATHWAY | -0.337091564 | 0.416558718 | 1.124244644 | -1.203711798 |
| FC_GAMMA_R_MEDIATED_PHAGOCYTOSIS | 0.00998236 | -0.426193775 | 1.376789422 | -0.960578006 |
| LEUKOCYTE_TRANSENDOTHELIAL_MIGRATION | -0.030077588 | -0.171183278 | 1.315031243 | -1.113770377 |
| INTESTINAL_IMMUNE_NETWORK_FOR_IGA_PRODUCTION | -0.104291667 | 0.163321679 | 1.18718278 | -1.246212792 |
| LONG_TERM_POTENTIATION | 0.283858874 | -1.365159092 | 0.055284415 | 1.026015803 |
| NEUROTROPHIN_SIGNALING_PATHWAY | -0.402266619 | -0.28228617 | 1.465706371 | -0.781153583 |
| LONG_TERM_DEPRESSION | 0.318687133 | -1.461812893 | 0.338997484 | 0.804128276 |
| OLFACTORY_TRANSDUCTION | 0.374687179 | -1.356476584 | 1.009366291 | -0.027576885 |
| REGULATION_OF_ACTIN_CYTOSKELETON | -0.377154266 | -0.048253566 | 1.388488833 | -0.963081001 |
| INSULIN_SIGNALING_PATHWAY | 0.413649766 | 0.690848383 | 0.380934805 | -1.485432953 |
| GNRH_SIGNALING_PATHWAY | -0.710893743 | 0.942019053 | 0.773403243 | -1.004528553 |

| | | | | |
|---|---|---|---|---|
| PROGESTERONE_MEDIATED_OOCYTE_MATURATION | -0.709300295 | 0.561265811 | -0.967836077 | 1.115870561 |
| MELANOGENESIS | -1.125098755 | 0.50227884 | 1.11388003 | -0.491060115 |
| ADIPOCYTOKINE_SIGNALING_PATHWAY | 0.341531899 | -0.027676686 | 1.033311323 | -1.347166535 |
| TYPE_II_DIABETES_MELLITUS | -0.822087475 | -0.902961584 | 0.760212674 | 0.964836386 |
| TYPE_I_DIABETES_MELLITUS | -0.086758667 | 0.822600952 | 0.643283256 | -1.379125542 |
| MATURITY_ONSET_DIABETES_OF_THE_YOUNG | 0.078252968 | -1.415026623 | 0.446921001 | 0.889852655 |
| ALDOSTERONE_REGULATED_SODIUM_REABSORPTION | -0.88372673 | 0.055966879 | 1.381687135 | -0.553927284 |
| VASOPRESSIN_REGULATED_WATER_REABSORPTION | 0.38913634 | -1.278540063 | 1.084384878 | -0.194981155 |
| ALZHEIMERS_DISEASE | 0.508268966 | 0.255362945 | 0.710174333 | -1.473806243 |
| PARKINSONS_DISEASE | 0.502165132 | 1.055090295 | -0.319230053 | -1.238025373 |
| AMYOTROPHIC_LATERAL_SCLEROSIS_ALS | 0.066802896 | -0.916373991 | 1.372126711 | -0.522555616 |
| HUNTINGTONS_DISEASE | 0.114075855 | 1.371458131 | -0.779398144 | -0.706135842 |
| PRION_DISEASES | -0.360804174 | -0.655154819 | 1.488924493 | -0.4729655 |
| VIBRIO_CHOLERAE_INFECTION | 0.848217136 | -0.954245546 | 0.878944624 | -0.772916214 |
| EPITHELIAL_CELL_SIGNALING_IN_HELICOBACTER_PYLORI_INFECTION | 0.058441482 | -0.209150639 | 1.288096096 | -1.137386939 |
| LEISHMANIA_INFECTION | 0.031522268 | 0.073832804 | 1.169616458 | -1.274971531 |
| PATHWAYS_IN_CANCER | -0.593591357 | 0.230546303 | 1.305932783 | -0.942887729 |
| COLORECTAL_CANCER | -1.138469405 | 1.291433438 | 0.033944638 | -0.186908671 |
| RENAL_CELL_CARCINOMA | -0.504503364 | 0.042573274 | 1.379218973 | -0.917288884 |
| PANCREATIC_CANCER | -1.215686328 | 1.067832717 | 0.504571494 | -0.356717883 |
| ENDOMETRIAL_CANCER | 0.693594217 | -1.480940996 | 0.482508495 | 0.304838284 |
| GLIOMA | 0.805274854 | -1.411082445 | 0.005517411 | 0.600290181 |
| PROSTATE_CANCER | -0.199160612 | -0.150954151 | 1.374277487 | -1.024162724 |
| THYROID_CANCER | -0.555858107 | -0.303451163 | 1.48552701 | -0.626217741 |
| BASAL_CELL_CARCINOMA | 0.367190297 | 1.055651598 | -1.319093335 | -0.10374856 |
| MELANOMA | -0.835450188 | -0.464973779 | 1.437698847 | -0.137274879 |
| BLADDER_CANCER | -1.074286915 | 0.497647618 | 1.134486899 | -0.557847602 |
| CHRONIC_MYELOID_LEUKEMIA | -0.983945564 | 0.331955072 | 1.250416999 | -0.598426507 |
| ACUTE_MYELOID_LEUKEMIA | -0.353578166 | -0.24189878 | 1.446470614 | -0.850993668 |
| SMALL_CELL_LUNG_CANCER | -0.560471941 | 0.815651021 | 0.869412575 | -1.124591654 |
| NON_SMALL_CELL_LUNG_CANCER | -0.913781211 | 0.118441274 | -0.560112042 | 1.355451978 |
| ASTHMA | 0.103681206 | 0.388841627 | 0.919234835 | -1.411757668 |
| AUTOIMMUNE_THYROID_DISEASE | -0.138450103 | 0.054663904 | 1.261387536 | -1.177601337 |
| ALLOGRAFT_REJECTION | -0.027729161 | 0.681467694 | 0.750429634 | -1.404168167 |
| GRAFT_VERSUS_HOST_DISEASE | -0.144256999 | 0.384136965 | 1.063879579 | -1.303759545 |
| VIRAL_MYOCARDITIS | -0.417244488 | 0.889591754 | 0.744381715 | -1.21672898 |
| MAPK_SIGNALING_PATHWAY | -0.24587582 | -0.122486808 | 1.379316568 | -1.01095394 |
| ERBB_SIGNALING_PATHWAY | 0.037444441 | -1.379668605 | 0.982824771 | 0.359399393 |
| PI3KAKT_SIGNALING_PATHWAY | -0.257816757 | -0.158080147 | 1.395815462 | -0.979918559 |
| HERPES_SIMPLEX_VIRUS_1_INFECTION | -0.202612444 | 0.601807728 | 0.922252886 | -1.321448171 |
| PROTEIN_DIGESTION_AND_ABSORPTION | -0.827646482 | 0.059224558 | 1.388267142 | -0.619845218 |
| STAPHYLOCOCCUS_AUREUS_INFECTION | -0.355348728 | 0.144314438 | 1.295187874 | -1.084153584 |

| | | | | |
|---|---|---|---|---|
| HUMAN_PAPILLOMAVIRUS_INFECTION | -0.064583518 | 0.479861077 | 0.949801196 | -1.365078754 |
