# Supplementary material for: Patient-Derived Tumor Organoids for Guidance of Personalized Drug Therapies in Recurrent Glioblastoma
Source: Int J Mol Sci. 2022 Jun 12;23(12):6572. doi: 10.3390/ijms23126572 (PMC9223608; doi:10.3390/ijms23126572)
Supplement: Supplementary file 1 [file ijms-23-06572-s001.zip › TableS4.pdf]

Table S4. AUCs from a comparative response analysis of calcein-AM- and ATP-based measurements.

	Calcein AM	ATP-based measurement
MK2206	986,6	835,5
Enzalutamide	1063	1071
Flutamide	1095	1034
KU-55933	1134	1067
KU-60019	1095	978,8
AZ32	1135	1108
Berzosertib	941,8	762,2
AZD6738	1014	834
Alisertib	1030	820,3
ENMD-2076	1020	820,3
Navitoclax	892,3	702,8
AZD5153	955,2	733,3
I-BET-762	1009	930,6
Acalabrutinib	1001	950,9
Tasisulam	963,5	871,1
Alvocidib	789,2	566,8
Palbociclib	906	720,5
Ribociclib	948,9	806,6
Abemaciclib	873	743,9
THZ1	756,4	495,6
Prexasertib	921,7	532,2
Rabusertib	971,4	670,4
MK-8776	1038	776
PF-477736	966	714
Selinexor	891,9	497,6
Carboplatin	1152	1024
Oxaliplatin	1111	1058
NU7441	1254	1142
Thalidomide	1157	1078
Gefitinib	1108	1017
Lapatinib	1125	969,4
Afatinib	943	661,1
Zibotentan	1100	915,9
Tazemetostat	1108	921,1
LNT1	1047	866,1
AZD4547	968,1	702,2
RRx-001	929,8	732,4
RO4929097	1055	799,6
Belinostat	986,1	699,7
Entinostat	986,5	642,9
Romidepsin	611,8	49,29
Tasquinimod	1097	1023

Itraconazole	1120	1020
Lonafarnib	1018	832,1
Ispinesib	862,8	543,1
Imatinib	1093	919,1
Masitinib	987,4	824,9
Nutlin-3	1046	854,7
Binimetinib	931,7	758,5
Pimasertib	908,1	679,6
SU11274	1139	986,1
Crizotinib	940,2	659,1
Patupilone	868,8	475,1
Vinorelbine	800,8	352,9
Paclitaxel	906	542
Mirin	1077	897,9
Everolimus	908,3	568,8
Temsirolimus	916,7	584,9
Regorafenib	1063	893,8
APR-246	999,4	913,1
ICG-001	1186	925,8
PHA-665752	1133	817,3
Tivantinib	1153	874,6
Decitabine	1210	1033
Doxorubicin	1045	543,7
BLU-9931	1108	820,6
Resminostat	1184	1008
Tanespimycin	943,7	341,2
Alvespimycin	898,7	176,7
Linsitinib	1202	1065
Selumetinib	1249	880,7
Refametinib	1149	751,3
Trametinib	1119	629,3
Vinblastine	1024	411,1
Paclitaxel	1128	602,8
Rapamycin	1099	669,8
PF-04691502	1071	601,4
Dasatinib	1231	1028
Brivanib	1312	1149
Lenvatinib	1317	1121
